# Supplementary material for: Spatial mapping of DNA synthesis reveals dynamics and geometry of human replication nanostructures
Source: EMBO J. 2025 Oct 7;44(23):7263–94. doi: 10.1038/s44318-025-00574-2 (PMC12669658; doi:10.1038/s44318-025-00574-2)
Supplement: Supplementary file 1 — Appendix [file 44318_2025_574_MOESM1_ESM.pdf]

Appendix for *The EMBO Journal* article “Spatial mapping of DNA synthesis reveals dynamics and geometry of human replication nanostructures” by *Hawgood M., Urién, B. Agostinho A., Thiagarajan P., Giglio G., Yang Y., Zhang X., Quijada G., Fonseca M., Bartek J., Blom H., Lemmens B.*

Table of Contents:

- Appendix Protocol
  - 3D-SPARK Protocol
  - SIM workflow
  - XMARK workflow
  - Volume Calculator leaflet

3D-SPARK Protocol

1. Cover slides

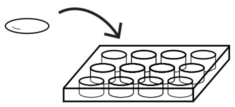

- Sterilise 12 mm glass cover slides with ethanol.
- Place one inside each well of a cell culture plate.
- Allow ethanol to evaporate.

2. Seed cells

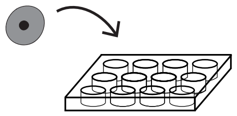

- Seed cells on the coverslips in 2 ml media per well.
  - Adjust density so that cells are 80% confluent when performing DNA labelling.
- Allow cells to adhere and grow overnight.

3. Treatments

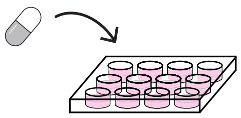

- Perform any desired drug treatments or esiRNA transfections as desired prior to DNA pulse labelling.

4. Nascent DNA labelling

a. Preparation

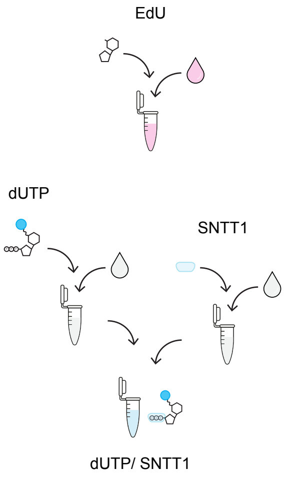

- Prepare a 100 µM EdU solution.
  - Mix 2 ml media with 20 µl EdU stock (10 mM).
  - This is enough for 10 samples, scale up as needed.
- Prepare dUTP (3 µM)/ SNTT1 (6 µM) mixture.
  - See Appendix 1.
  - A. Prepare the dUTP solution.
  - B. Prepare the SNTT1 solution.
  - C. Then combine the dUTP and SNNT1 solutions to create the final transfection mixture and keep on ice.

b. EdU pulse

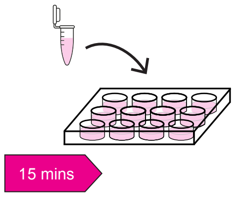

- Begin the 10 µM EdU pulse by adding 200 µl of the 50 µM EdU mixture per well.
- Incubate cells for 15 mins.

c. dUTP pulse

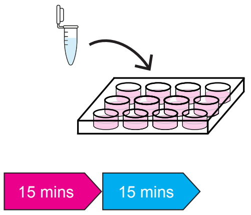

- Pre-heat the transfection mixture at 37°C, for max 3 mins.
- Remove media and EdU.
- Add 530 µl of the dUTP-SNTT1 transfection mixture per sample.
- Incubate cells for 4 mins.
- Remove transfection mixture.
- Add 1 ml warm (37°C) media
- Incubate cells for the remaining 11 mins.

5. Fix cells

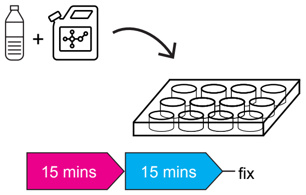

- Remove media and wash twice with PBS.
- Add 1 ml 4% PFA to each well for 7 mins. Remove PFA and add 1 ml ice cold methanol for 2 mins.
- Remove methanol.
- Wash: 3 x PBS.
- Store fixed samples at 4°C.

3D-SPARK Protocol

3D-SIM

Continue to follow the instructions on this page if you wish to perform 3D-SIM. To perform XMARK, please see the next pages.

6. EdU click chemistry

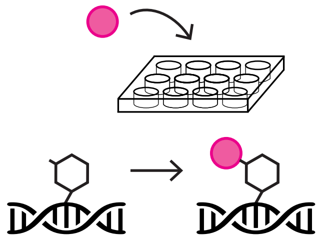

- Prepare EdU click reaction mixture.
  - See Appendix 1.
- Add 550 µl click mix per well.
- Incubate for 1 hour at room temperature, on a rocking table, protected from light.
- Wash: 1 x PBS, 2 x TBST, 1 x PBS.

7. Immunofluorescence

a. Blocking

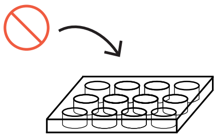

- Block cells in 2% BSA in TBS/T for 60 mins at room temperature, on a rocking table.

b. Primary antibody

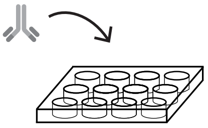

- Prepare primary antibody mixture according to your standard protocol.
- Remove blocking buffer.
- Add 500 µl antibody mixture per well.
- Incubate sample overnight, at 4°C, on a rocking table, protected from light.
- Wash: 3 x PBS.

c. Secondary antibody and DAPI

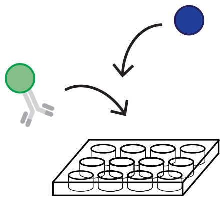

- Prepare secondary antibody mixture according to standard protocol, with 50ng/ml DAPI.
- Add 500 µl antibody-DAPI mixture per well.
- Incubate sample for 1 hour, at room temperature, on a rocking table, protected from light.
- Wash: 3 x PBS.

8. Mount

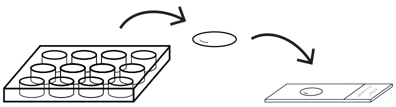

- Carefully remove stained cover slides from the wells of the culture plate.
- Mount on to a glass adhesion slide (cell side down) using ProLong Gold Antifade Mountant.
- Leave overnight to cure.
- Seal and fix coverslips by applying small amounts of nail polish to the edges.

9. Microscopy

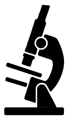

- Perform 3D-SIM.

10. Analysis

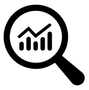

- Analyse 3D-SIM images.
- Please find our analysis instructions in the methods section in the online version of the paper.

3D-SPARK Protocol

XMARK

Continue to follow the instructions on this page if you wish to perform XMARK. To perform 3D-SIM, please see the previous page.

6. Gelation

a. Anchoring

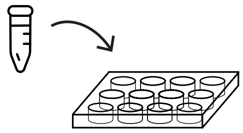

- Mix the anchoring solution.
  - See Appendix 1.
- Add 1 ml anchoring solution per well.
- Incubate for 4 hours, at 37°C, in a humidified chamber, protected from light.
- Safely remove and discard the anchoring solution.
- Wash: 3 x 5 mins PBS.

b. Crosslinking polymerisation

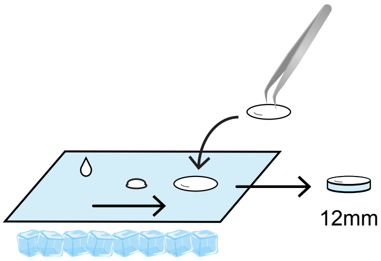

- Pre-chill a 500 µl aliquot of prepared MAP gel solution at 4°C.
  - See Appendix 1.
- Prepare a lid of a cell culture plate lid by wrapping it in parafilm and placing it on ice.
- Mix the crosslinking polymerisation solution:
  - Add 25 µl APS (10%) stock
  - Add 25 µl TEMED (10%) stock
- Immediately after adding TEMED, add 35 µl of the crosslinking polymerisation solution onto the parafilm, creating droplets, 1 per sample.
- Using tweezers, carefully place the coverslips on top of the formed droplet with cells facing down.
- Incubate for 5 mins on ice.
- Place the lid and samples in a humidified chamber and incubate for 1.5 hours at 37°C.

8. Denaturation

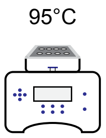

- Add 2 ml denaturation buffer to a 5 ml eppendorf tube
  - See methods section for instruction on how to prepare the denaturation buffer.
- Pre-heat the denaturation buffer to 95°C
  - 70°C for the first 10 minutes, release the pressure, then continue up to 95°C.
- Using tweezers, carefully transfer the hydrogel directly into the 5 ml tubes containing pre-heated denaturation buffer.
- Incubate for 1 hour in a heating block at 95°C, with closed lids.

9. Gel prepearation

a. Gel hydration

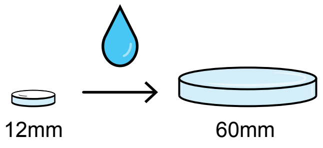

- Transfer the gel to a 10 cm plate.
  - Empty the entire tube, including gel and denaturation buffer.
- Wash and hydrate the hydrogels with excess dH_20 (exchanging dH_20 several times).
  - 3 x quick dH_20 washes
  - 1 x 10 min wash

3D-SPARK Protocol

9. Gel prepearation continued

a. Gel hydration continued

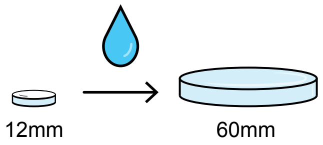

- Change dH_20 again and leave over night to achieve maximum expansion.
- Change dH_20 again once the next day.

b. Gel punching

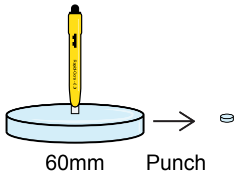

- Remove water and cut 2 sections of the gel using a tissue biopsy punch.
  - In the following steps, you will have 2 pieces of gel per sample to label.
- Transfer the cut portions of the gel to a new 12 well plate for staining.
- Add dH_20 back to the remainder of gel.

10. EdU click chemistry

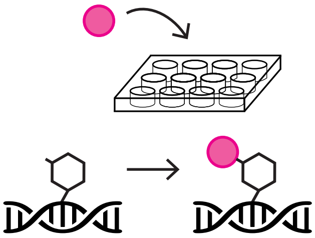

- Prepare EdU click reaction mixture.
  - See Appendix 1.
- Add 500 µl click mix per well.
- Incubate for 1.5 hours, at 37°C, in a humidified chamber, protected from light.
- Wash gels: 3 x PBS.

11. DAPI

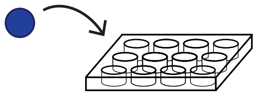

- Prepare 50 ng/ml DAPI mixture in 1 x PBS.
- Add 500 µl DAPI mix per well.
- Incubate for 1.5 hours, at 37°C, in a humidified chamber, protected from light.
- Wash gels: 3 x PBS.

12. Gel rehydration

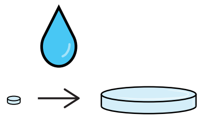

- Transfer the gels to a new 6 well plate.
- Cover gels in dH_20.
- Change dH_20 once.
- Leave overnight at 4°C, protected from light.
- Change dH_20 again once the next day.

13. Mount gels

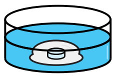

- Prepare Poly-L-Lysine coated imaging dishes.
  - Add 1 ml Poly-L-Lysine to an imagine dish.
  - Leave for 20 mins.
  - 3x dH_20 washes.
  - Allow to airdry.
- Find orientation of gels with cells face down using a microscope.
- Prepare 1% agarose.
- Add 1 ml 1% agarose to fix the gel to the imaging dish and allow to set.
- Add dH_20 on top.

14. Microscopy

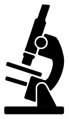

- Perform confocal microscopy on expanded samples.

15. Analysis

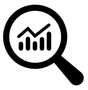

- Analyse expansion microscopy images.
- Please find our analysis instructions in the methods section in the online version of the paper.

3D-SPARK Protocol

Volume Calculators

Follow the instructions on this page to calculate the volumes needed for each mixture.

SNNT-dUTP Transfection Mixture		For 1 sample
A	Tricine buffer	296.4 µl
	dUTP (6 µM)	3.6 µl
B	Tricine buffer	296.4µl
	SNTT1 (6 µM)	3.6 µl
C	Mixture A (dUTP)	280 µl
	Mixture B (SNTT1)	280 µl

3D-SIM

EdU Click Mix	For 1 sample
dH_20	236.67 µl
1x PBS	299.67 µl
1 M Tris	30 µl
Cu_SO_4	2.67 µl
Azide dye	0.33 µl
1 M Ascorbic acid*	30 µl

*	Important to mix ascorbic acid last.

**	Prepare MAP gel solution according to description in the methods section.

***	Add TEMED last and only when ready to proceed.

XMARK

Anchoring	For 1 sample
40% acrylamide stock	1 ml
37% formaldehyde stock	140 µl
10x PBS	130 µl
dH_20	60 µl

MAP gel solution	For up to 15 samples
MAP gel solution**	500 µl
APS (10%)	25 µl
TEMED (10%)***	25 µl

EdU Click Mix	For 1 sample
dH_20	592 µl
1M Tris	75 µl
Cu_SO_4	6.75 µl
Azide dye	1.5 µl
1 M Ascorbic acid*	75 µl
