# Supplementary figures and images for: Spatial mapping of DNA synthesis reveals dynamics and geometry of human replication nanostructures
Source: EMBO J. 2025 Oct 7;44(23):7263–94. doi: 10.1038/s44318-025-00574-2 (PMC12669658; doi:10.1038/s44318-025-00574-2)

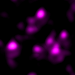

Supplement: Supplementary file 4 — Source data Fig. 1 [file 44318_2025_574_MOESM4_ESM.zip › Figure 1/1G/3_2min_cy5UTP_Image 64_event.png]

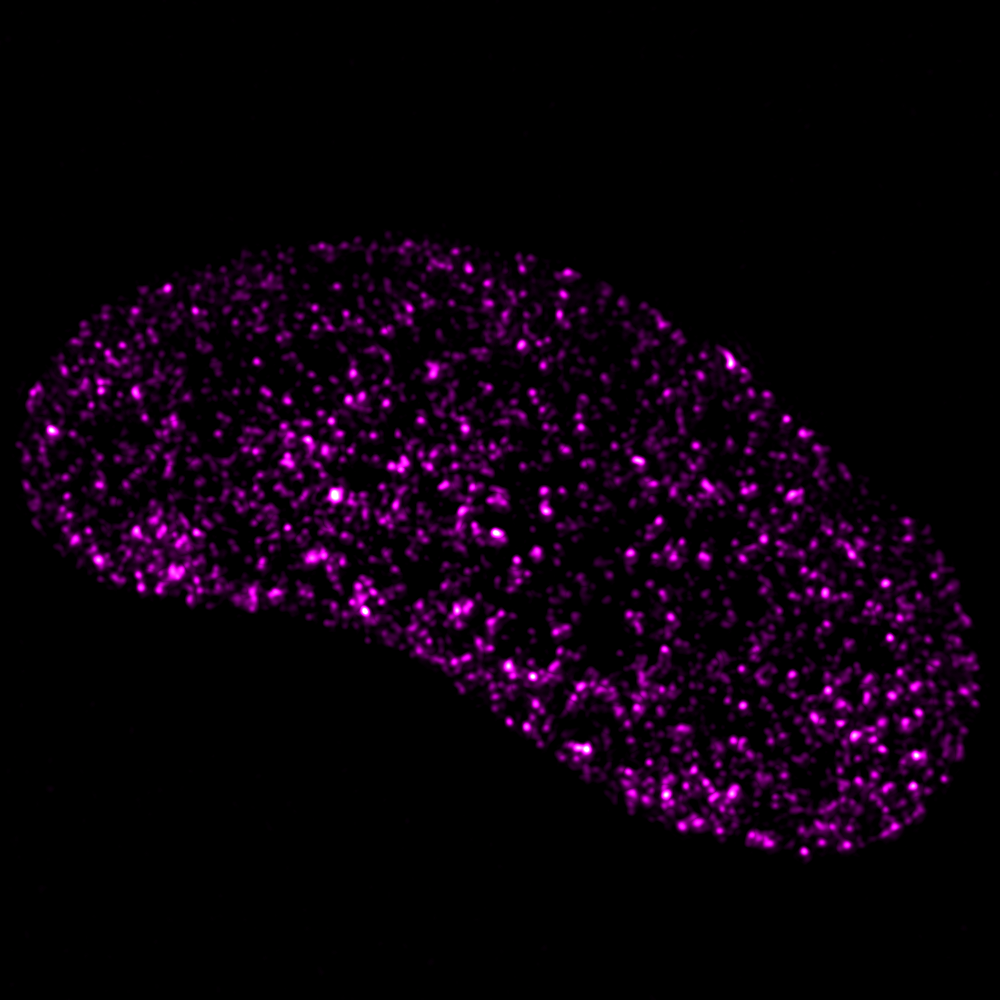

Supplement: Supplementary file 4 — Source data Fig. 1 [file 44318_2025_574_MOESM4_ESM.zip › Figure 1/1G/3_2min_cy5UTP_Image 64.png]

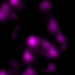

Supplement: Supplementary file 4 — Source data Fig. 1 [file 44318_2025_574_MOESM4_ESM.zip › Figure 1/1G/3_2min_cy5UTP_Image 70_event.png]

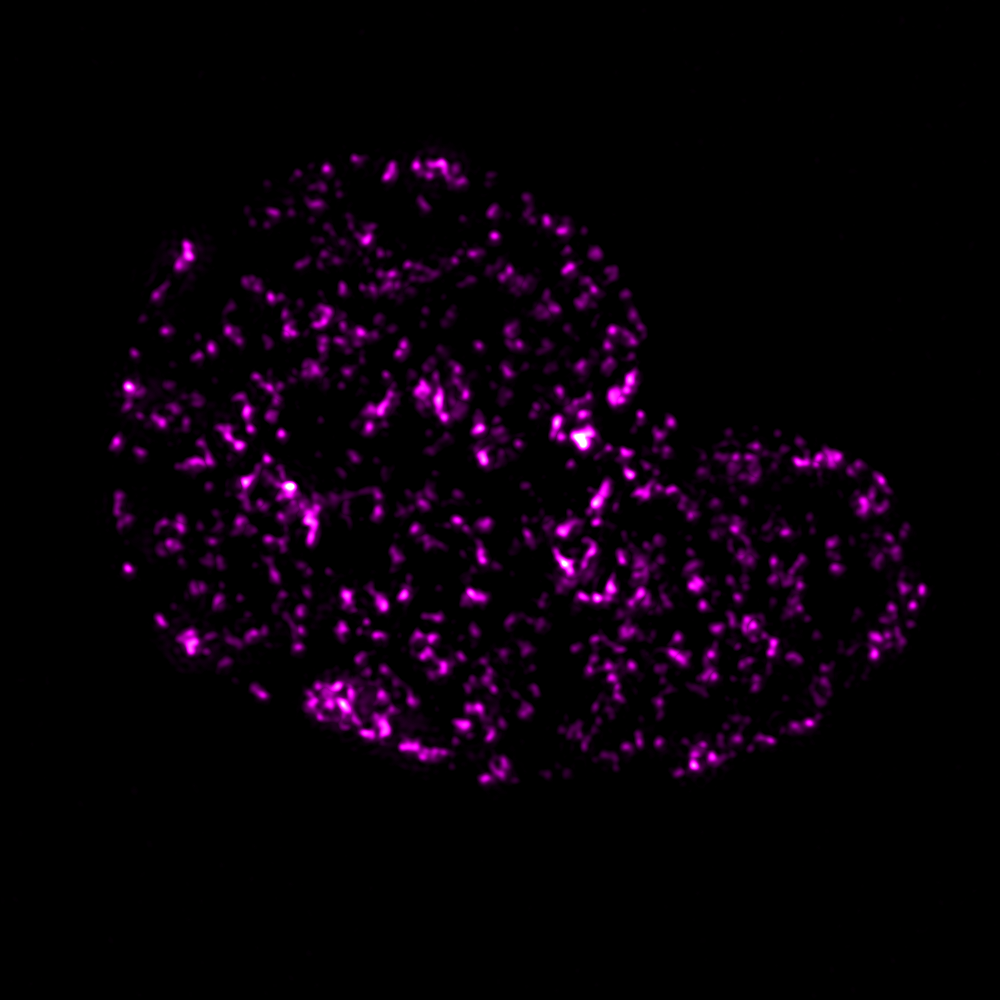

Supplement: Supplementary file 4 — Source data Fig. 1 [file 44318_2025_574_MOESM4_ESM.zip › Figure 1/1G/3_2min_cy5UTP_Image 70.png]

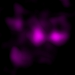

Supplement: Supplementary file 4 — Source data Fig. 1 [file 44318_2025_574_MOESM4_ESM.zip › Figure 1/1G/5minEDU_Image 73_event.png]

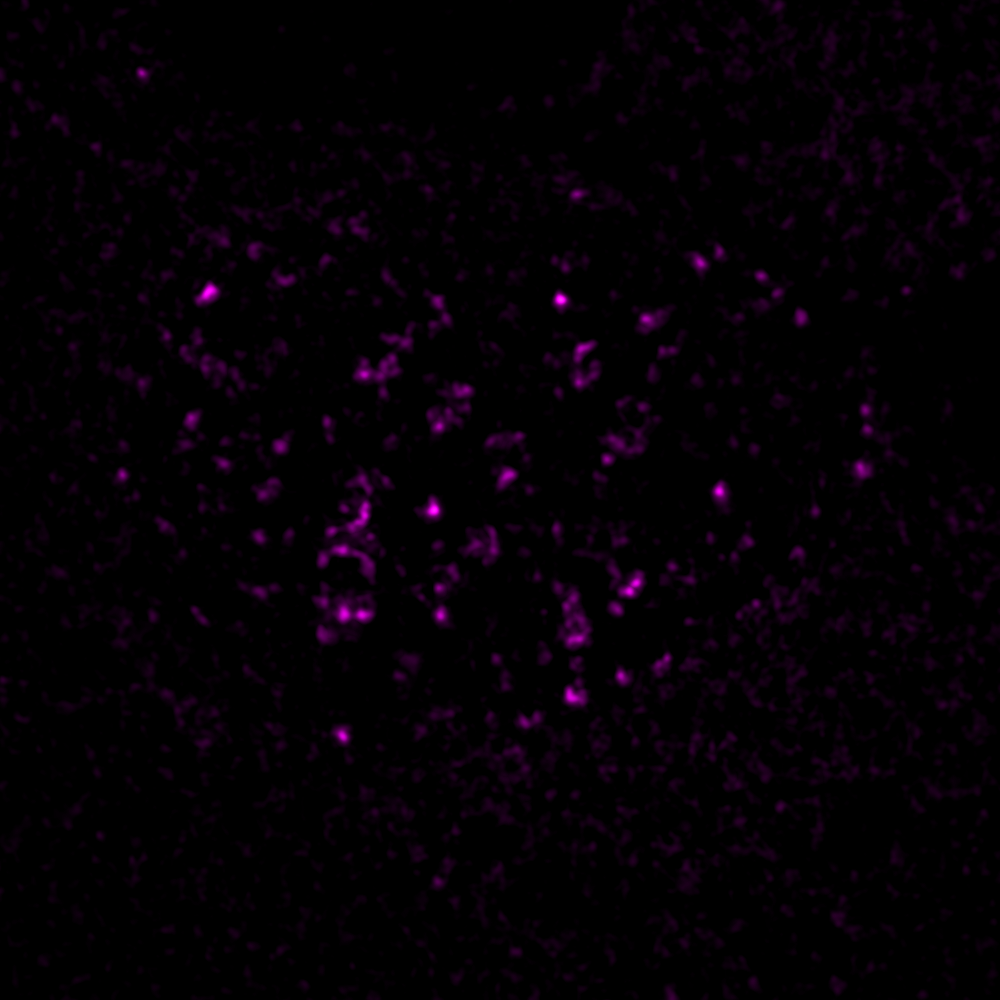

Supplement: Supplementary file 4 — Source data Fig. 1 [file 44318_2025_574_MOESM4_ESM.zip › Figure 1/1G/5minEDU_Image 73.png]

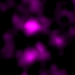

Supplement: Supplementary file 4 — Source data Fig. 1 [file 44318_2025_574_MOESM4_ESM.zip › Figure 1/1G/5minEDU_Image 78_event.png]

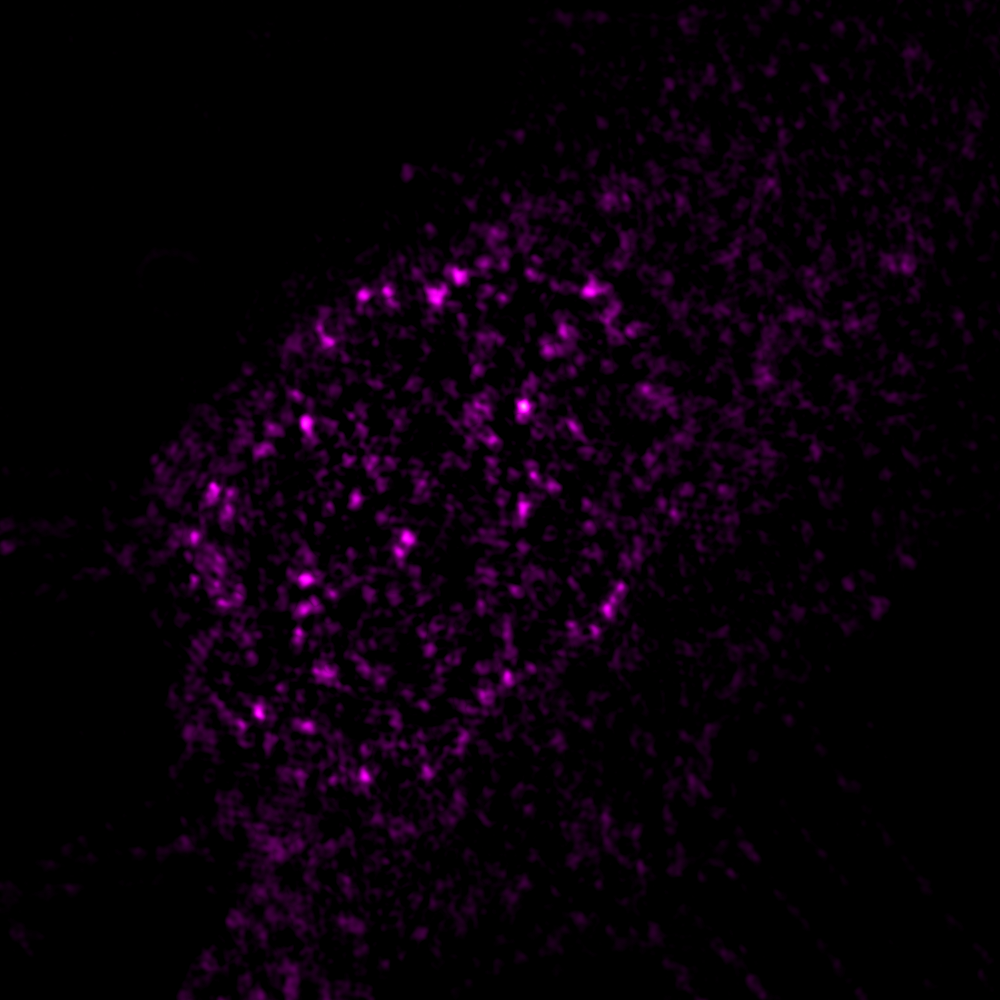

Supplement: Supplementary file 4 — Source data Fig. 1 [file 44318_2025_574_MOESM4_ESM.zip › Figure 1/1G/5minEDU_Image 78.png]

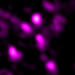

Supplement: Supplementary file 4 — Source data Fig. 1 [file 44318_2025_574_MOESM4_ESM.zip › Figure 1/1G/6_9min_cy5UTP_Image 23_event.png]

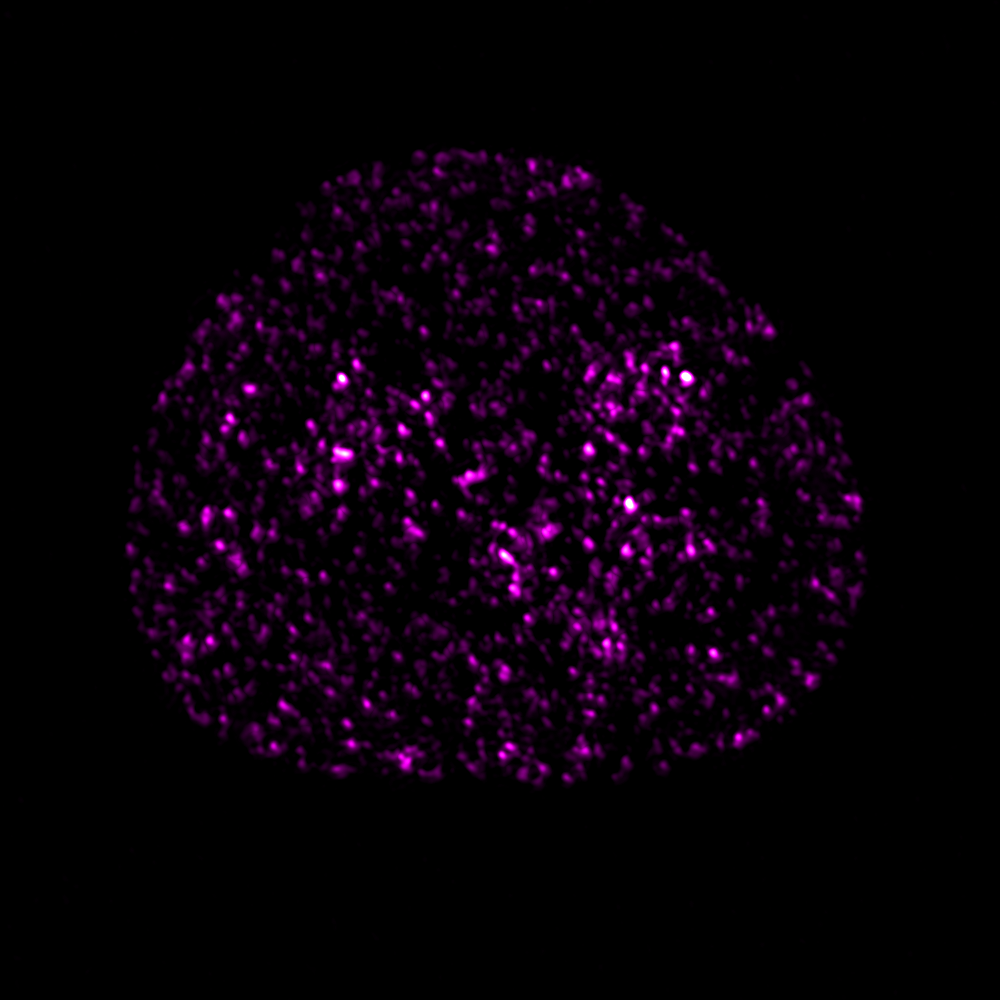

Supplement: Supplementary file 4 — Source data Fig. 1 [file 44318_2025_574_MOESM4_ESM.zip › Figure 1/1G/6_9min_cy5UTP_Image 23.png]

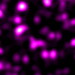

Supplement: Supplementary file 4 — Source data Fig. 1 [file 44318_2025_574_MOESM4_ESM.zip › Figure 1/1G/6_9min_cy5UTP_Image 29_event.png]

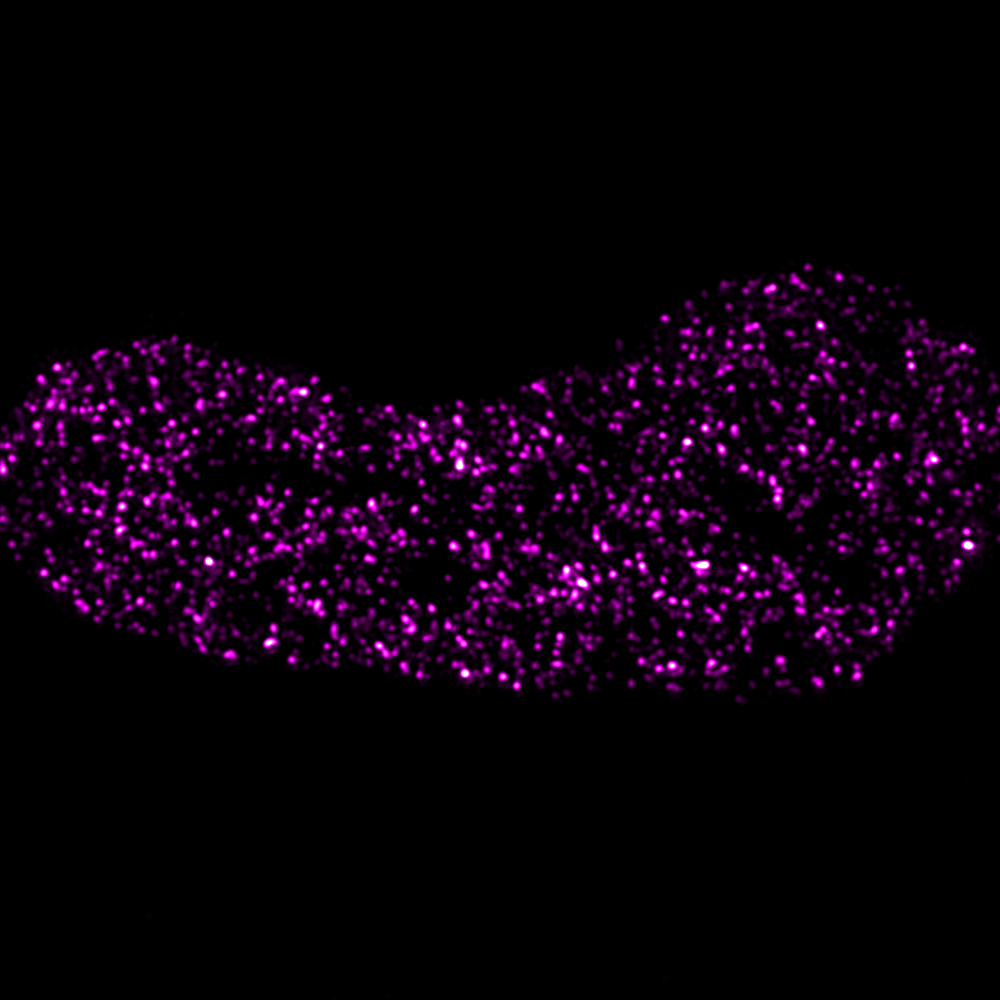

Supplement: Supplementary file 4 — Source data Fig. 1 [file 44318_2025_574_MOESM4_ESM.zip › Figure 1/1G/6_9min_cy5UTP_Image 29.png]

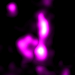

Supplement: Supplementary file 4 — Source data Fig. 1 [file 44318_2025_574_MOESM4_ESM.zip › Figure 1/1G/15minEDU_Image 43_event.png]

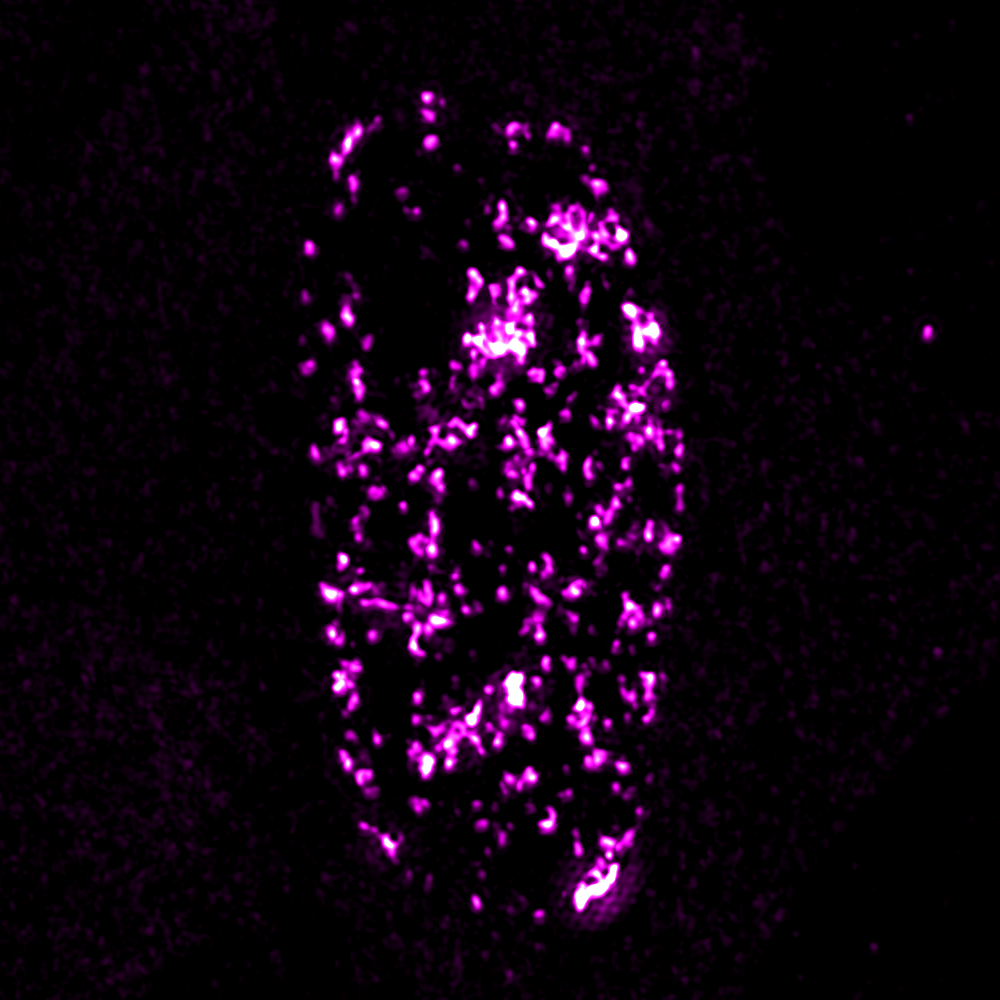

Supplement: Supplementary file 4 — Source data Fig. 1 [file 44318_2025_574_MOESM4_ESM.zip › Figure 1/1G/15minEDU_Image 43.png]

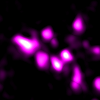

Supplement: Supplementary file 4 — Source data Fig. 1 [file 44318_2025_574_MOESM4_ESM.zip › Figure 1/1G/15minEDU_Image 49_event.png]

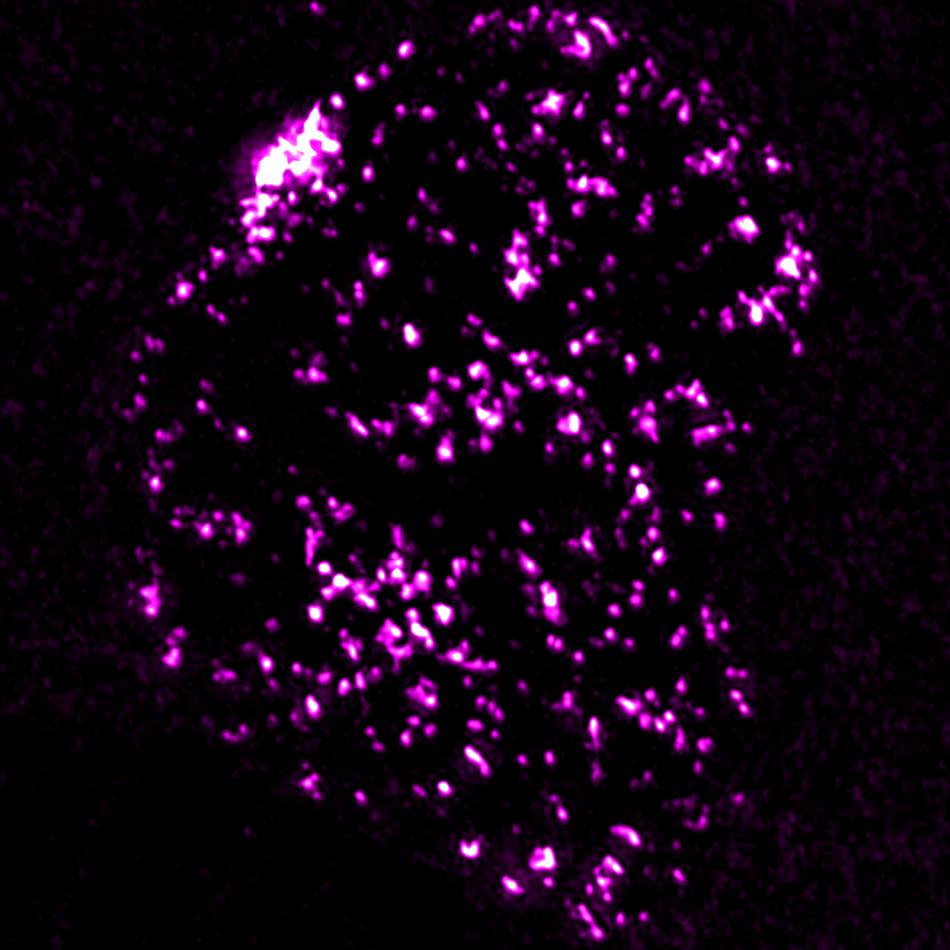

Supplement: Supplementary file 4 — Source data Fig. 1 [file 44318_2025_574_MOESM4_ESM.zip › Figure 1/1G/15minEDU_Image 49.png]

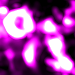

Supplement: Supplementary file 4 — Source data Fig. 1 [file 44318_2025_574_MOESM4_ESM.zip › Figure 1/1G/30minEDU_Image 6_event.png]

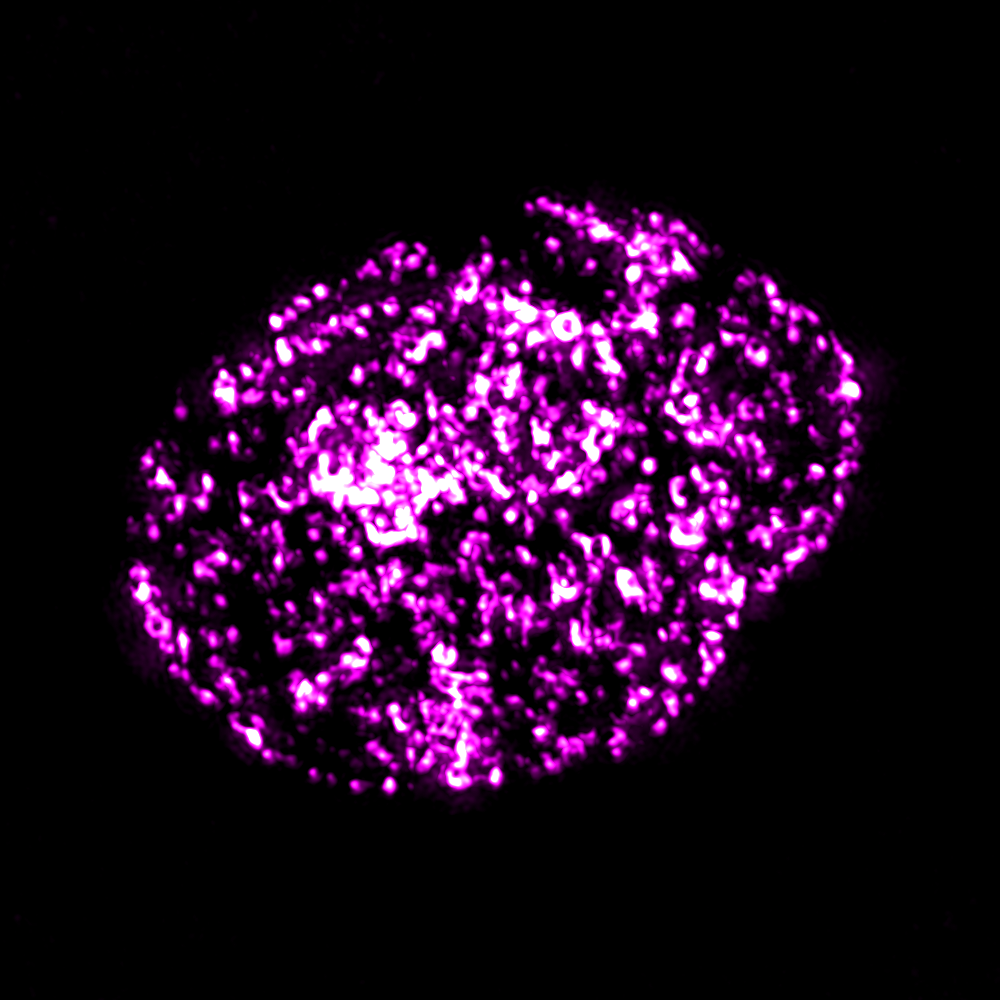

Supplement: Supplementary file 4 — Source data Fig. 1 [file 44318_2025_574_MOESM4_ESM.zip › Figure 1/1G/30minEDU_Image 6.png]

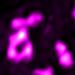

Supplement: Supplementary file 4 — Source data Fig. 1 [file 44318_2025_574_MOESM4_ESM.zip › Figure 1/1G/30minEDU_Image 14_event.png]

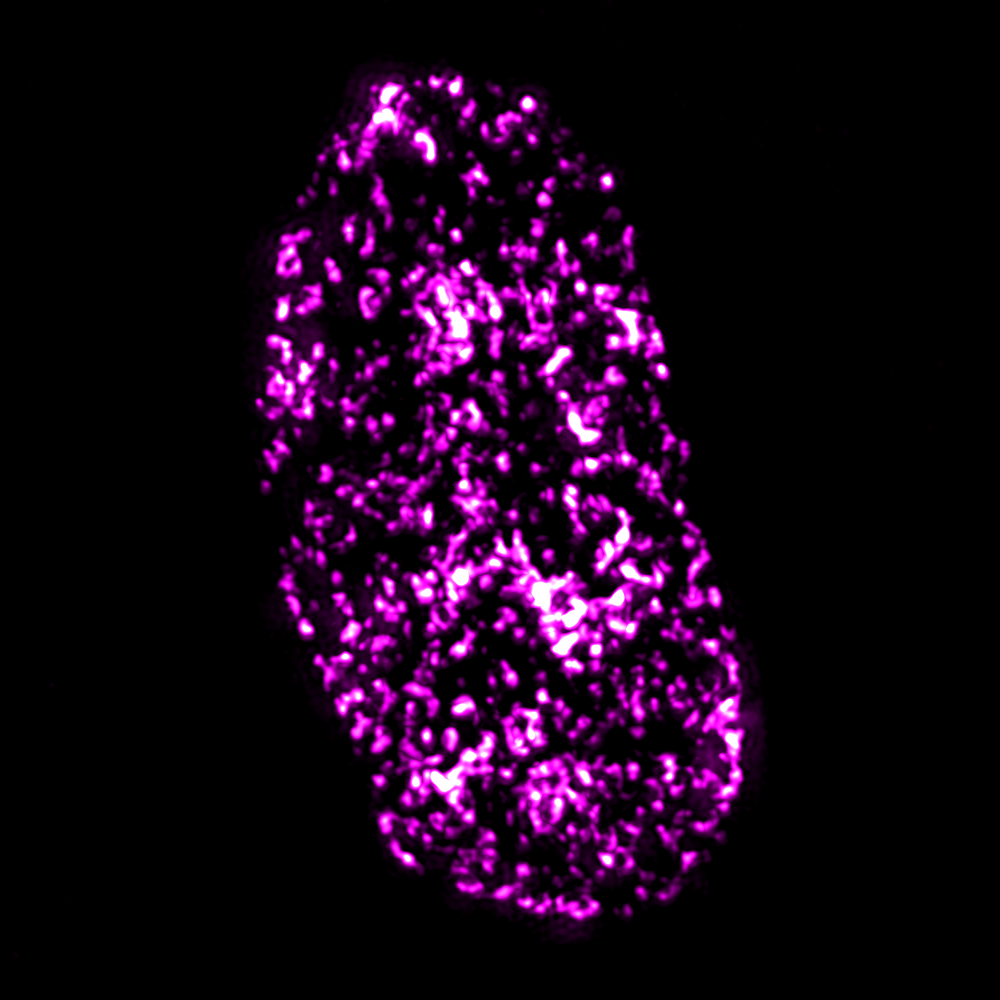

Supplement: Supplementary file 4 — Source data Fig. 1 [file 44318_2025_574_MOESM4_ESM.zip › Figure 1/1G/30minEDU_Image 14.png]

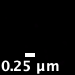

Supplement: Supplementary file 4 — Source data Fig. 1 [file 44318_2025_574_MOESM4_ESM.zip › Figure 1/1G/scale_bar_event.png]

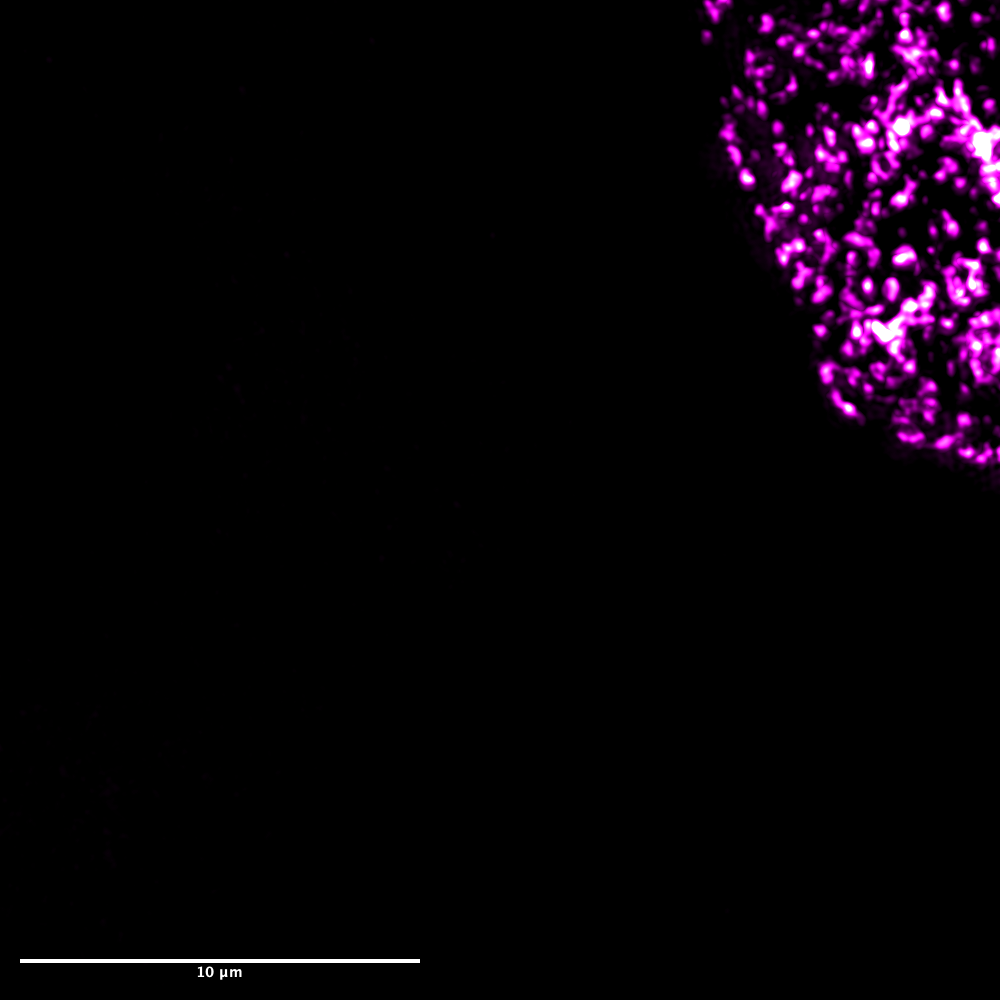

Supplement: Supplementary file 4 — Source data Fig. 1 [file 44318_2025_574_MOESM4_ESM.zip › Figure 1/1G/scale_bar_nuclei.png]

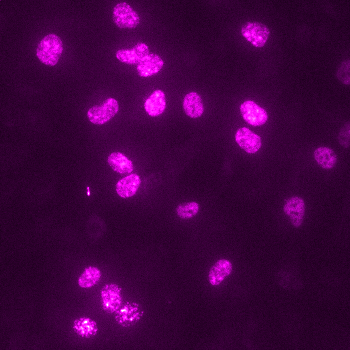

Supplement: Supplementary file 4 — Source data Fig. 1 [file 44318_2025_574_MOESM4_ESM.zip › Figure 1/1B/WellC05_ChannelDAPI,Cy5_1_Seq0013_XY10_Magenta Hot_EdU.png]

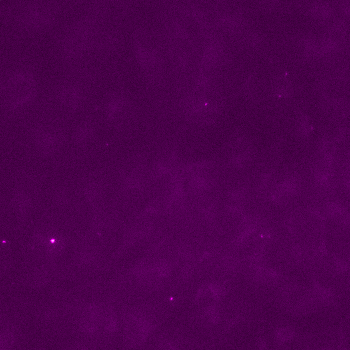

Supplement: Supplementary file 4 — Source data Fig. 1 [file 44318_2025_574_MOESM4_ESM.zip › Figure 1/1B/WellC10_ChannelDAPI,Cy5_1_Seq0018_XY01_Magenta Hot_EdU.png]

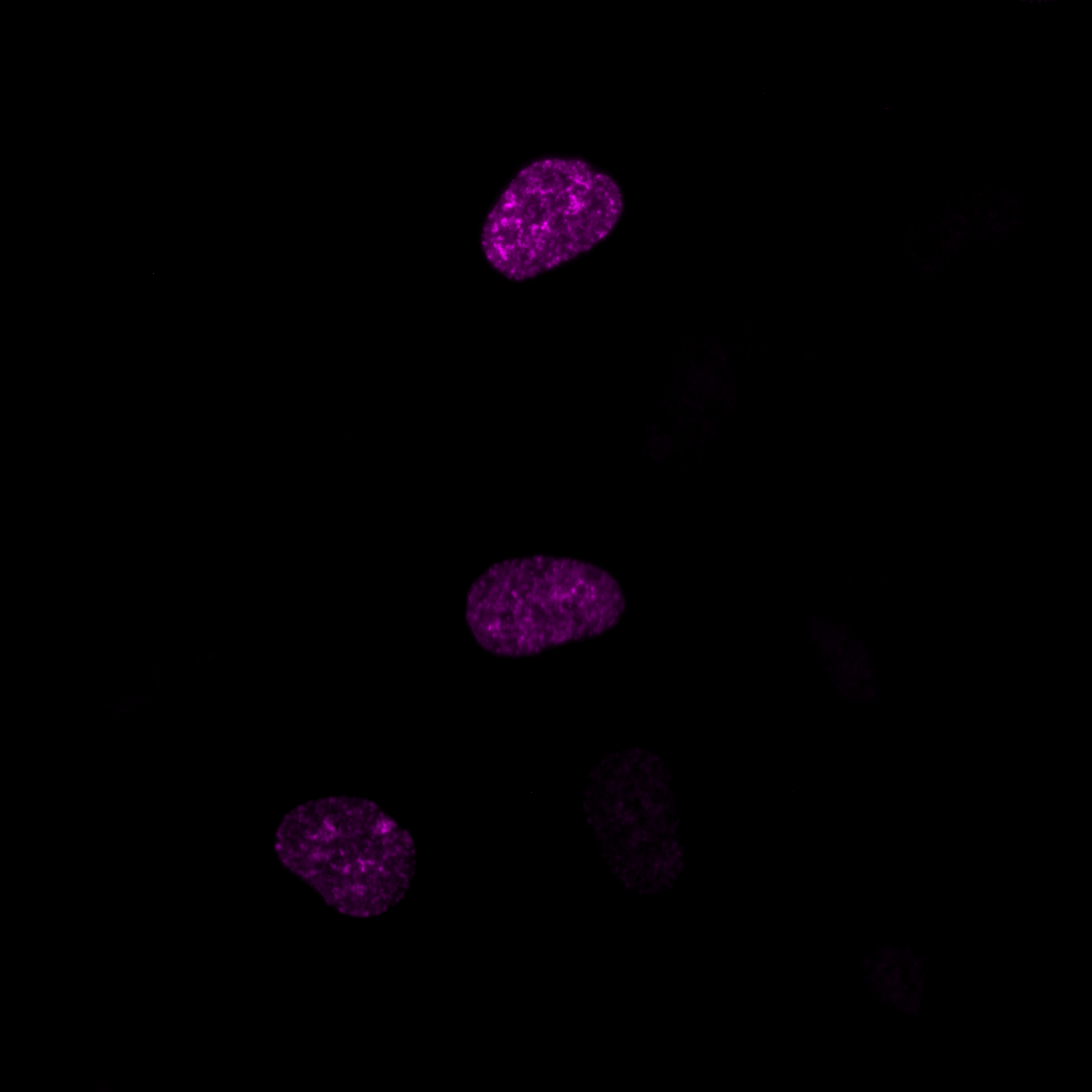

Supplement: Supplementary file 4 — Source data Fig. 1 [file 44318_2025_574_MOESM4_ESM.zip › Figure 1/1E/Cy5-dUTP_3+2min_2_ND Acquisition_Magenta Hot.png]

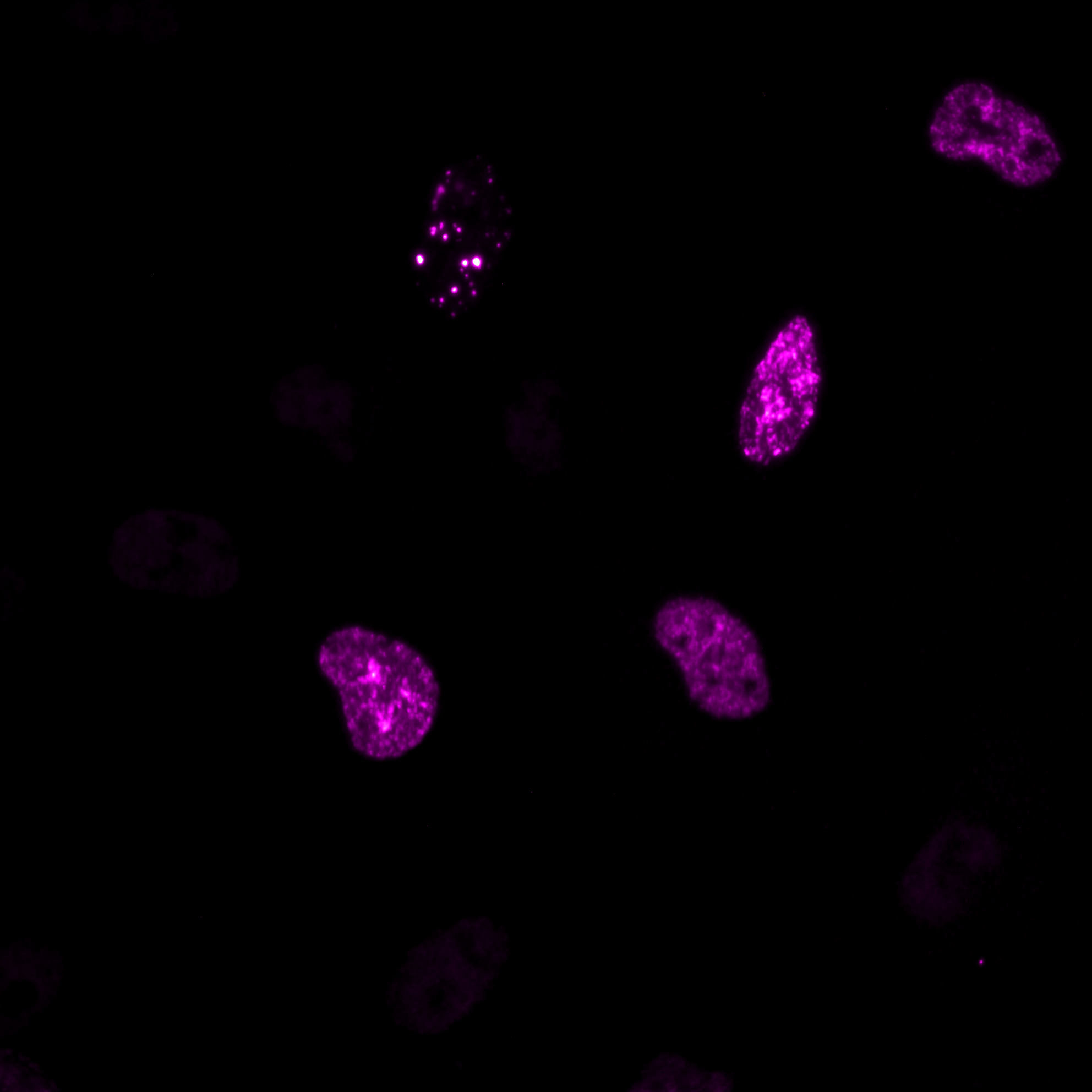

Supplement: Supplementary file 4 — Source data Fig. 1 [file 44318_2025_574_MOESM4_ESM.zip › Figure 1/1E/Cy5-dUTP_6+9min_2_ND Acquisition_Magenta Hot.png]

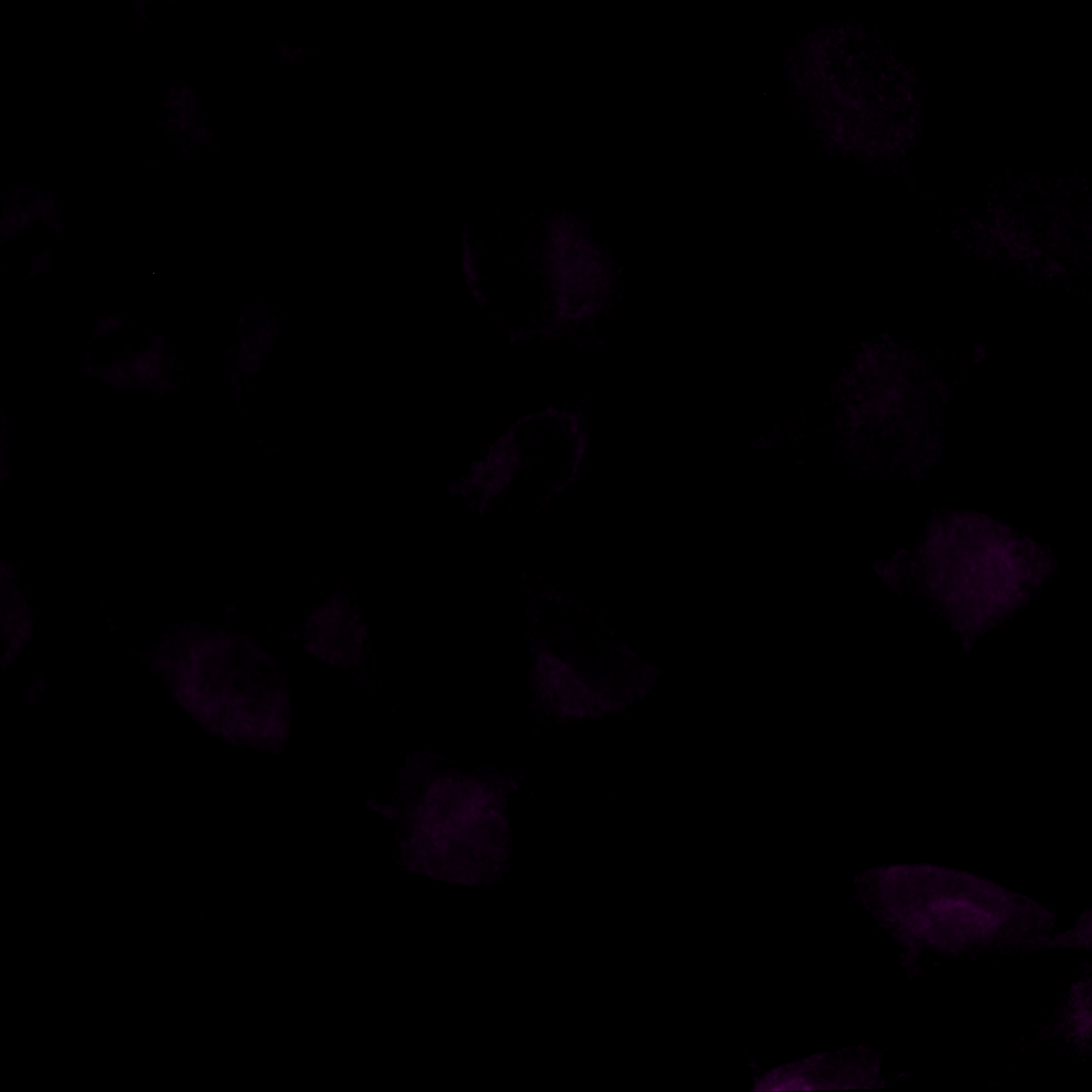

Supplement: Supplementary file 4 — Source data Fig. 1 [file 44318_2025_574_MOESM4_ESM.zip › Figure 1/1E/EdU_5min_1_ND Acquisition_Magenta Hot.png]

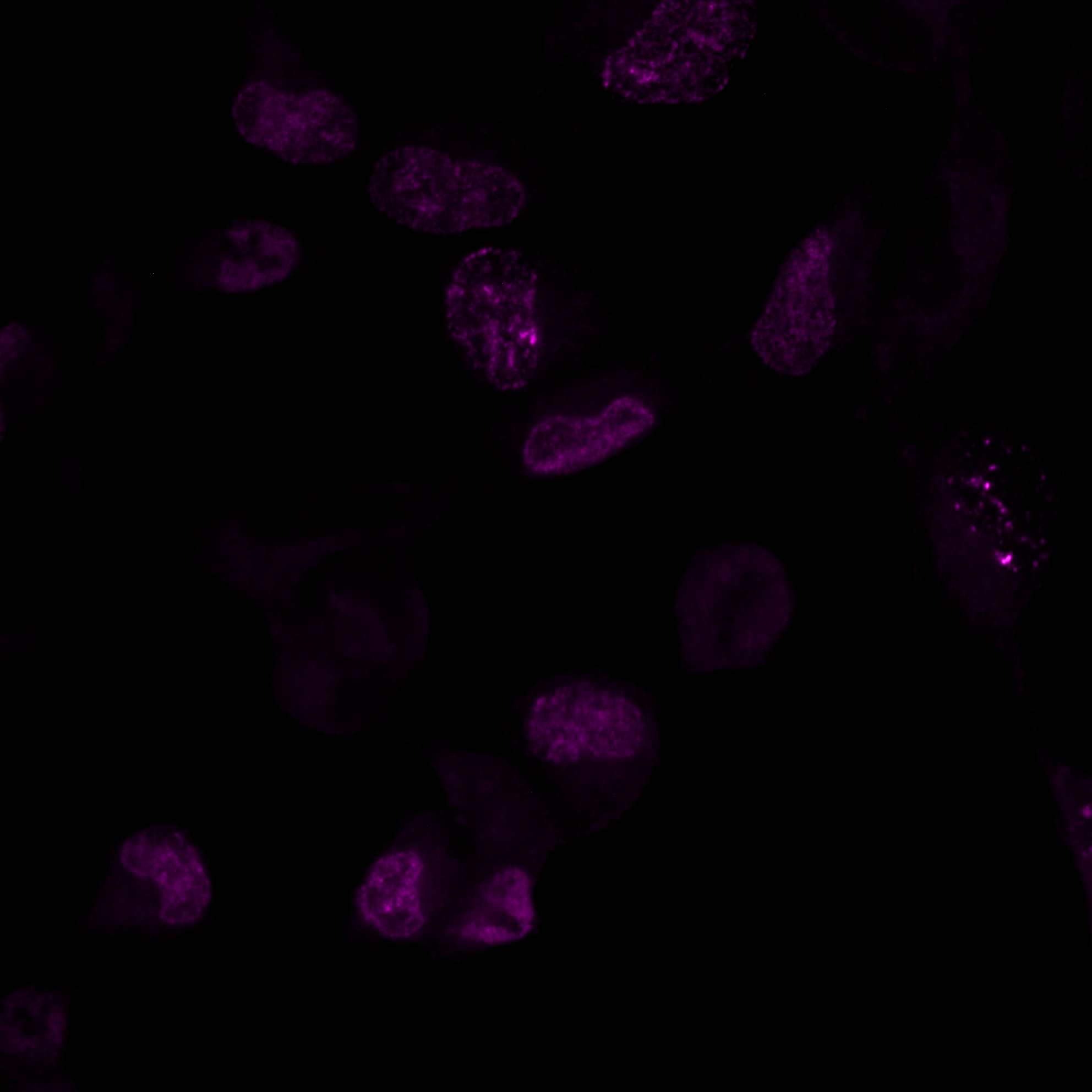

Supplement: Supplementary file 4 — Source data Fig. 1 [file 44318_2025_574_MOESM4_ESM.zip › Figure 1/1E/EdU_15min_2_ND Acquisition_Magenta Hot.png]

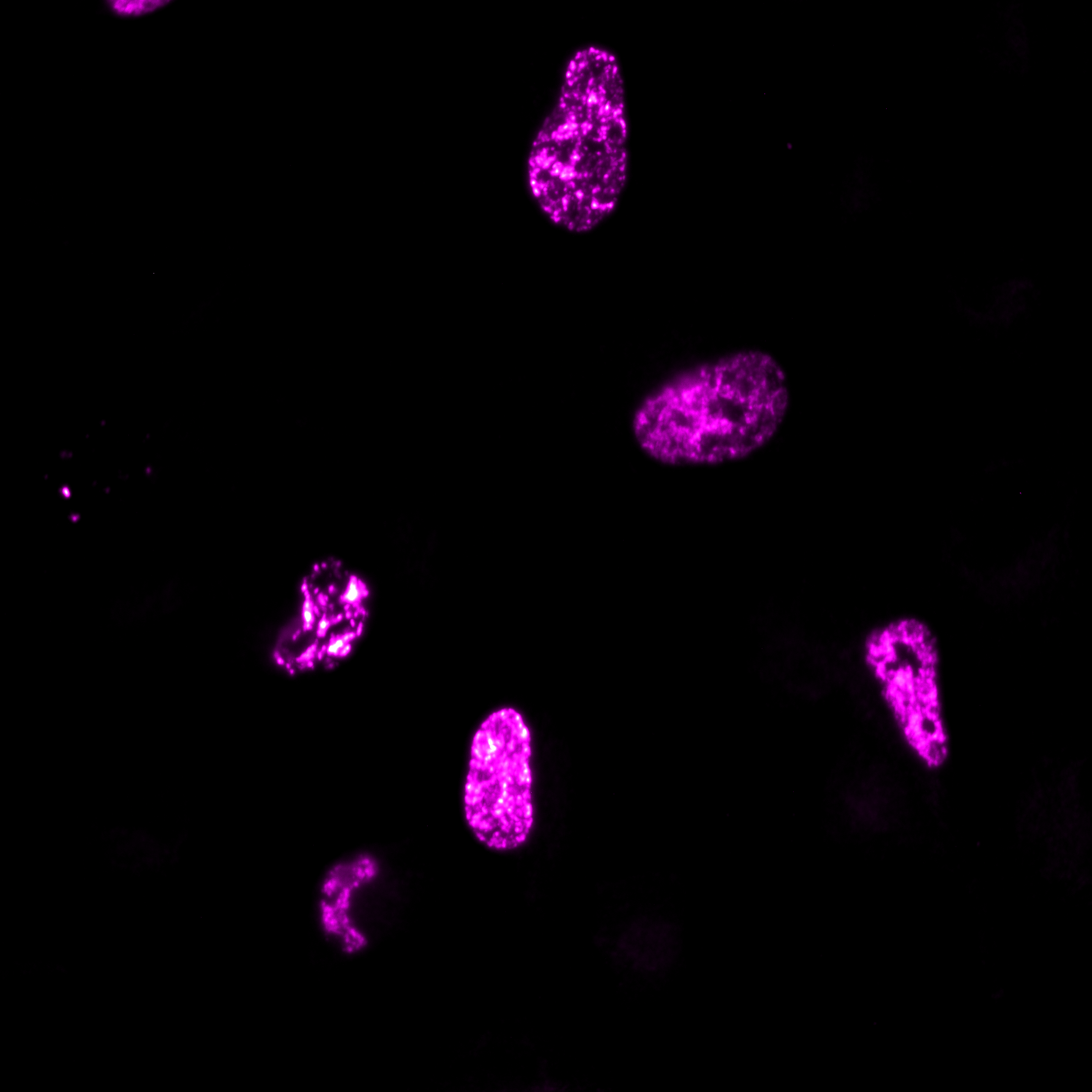

Supplement: Supplementary file 4 — Source data Fig. 1 [file 44318_2025_574_MOESM4_ESM.zip › Figure 1/1E/EdU_30min_2_ND Acquisition_Magenta Hot.png]

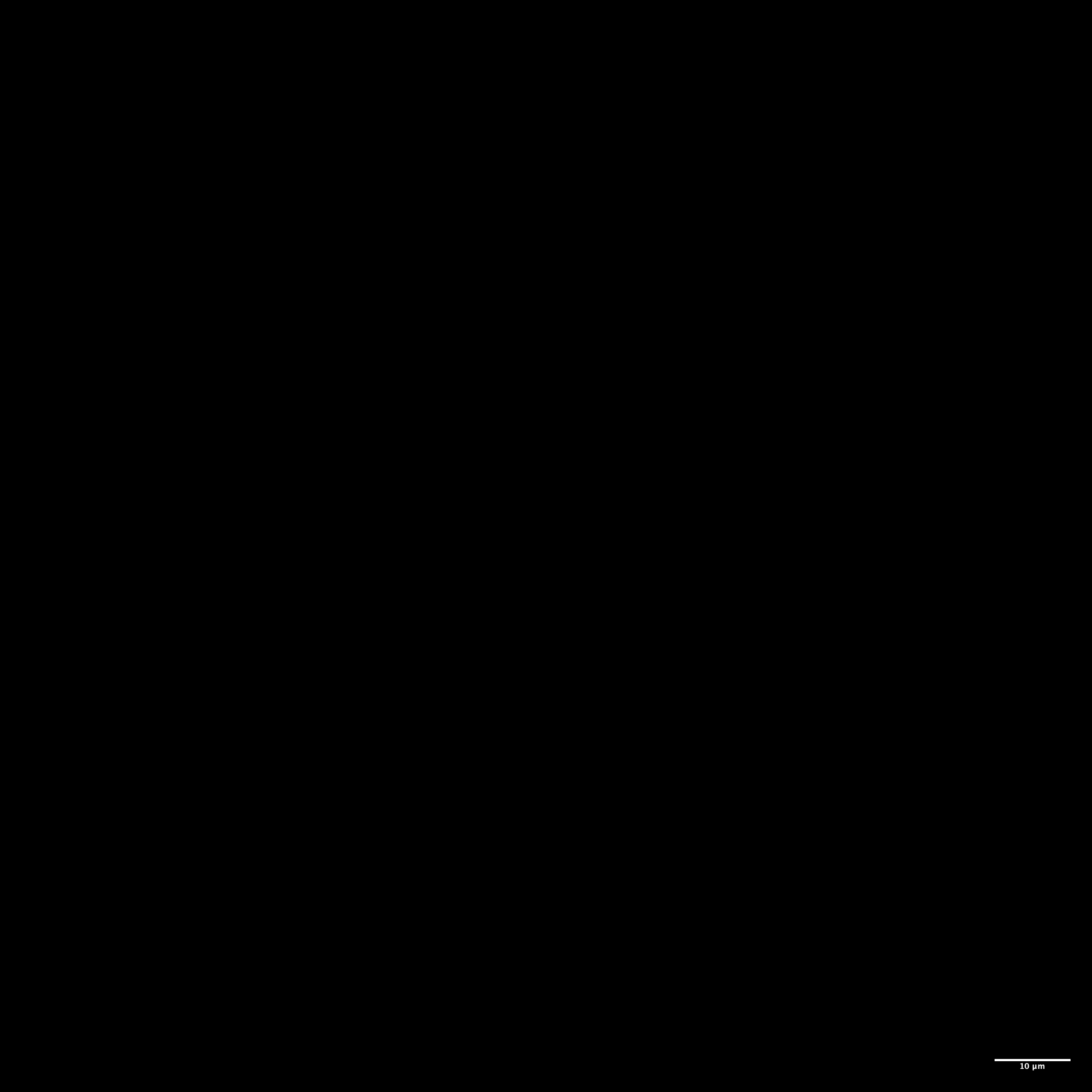

Supplement: Supplementary file 4 — Source data Fig. 1 [file 44318_2025_574_MOESM4_ESM.zip › Figure 1/1E/scale_bar.png]

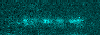

Supplement: Supplementary file 5 — Source data Fig. 2 [file 44318_2025_574_MOESM5_ESM.zip › Figure 2/2A/Fibre_cy3.png]

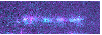

Supplement: Supplementary file 5 — Source data Fig. 2 [file 44318_2025_574_MOESM5_ESM.zip › Figure 2/2A/Fibre_merge.png]

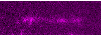

Supplement: Supplementary file 5 — Source data Fig. 2 [file 44318_2025_574_MOESM5_ESM.zip › Figure 2/2A/Fibre_cy5.png]

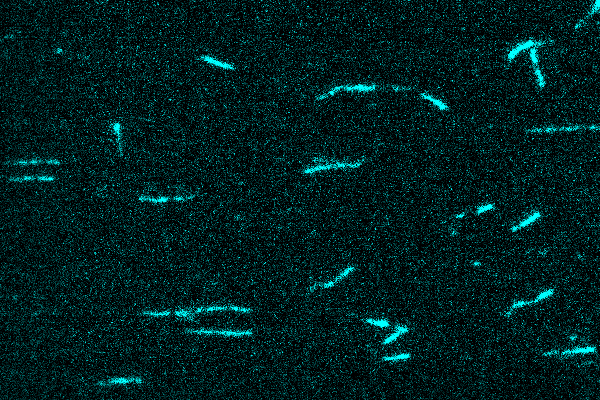

Supplement: Supplementary file 5 — Source data Fig. 2 [file 44318_2025_574_MOESM5_ESM.zip › Figure 2/2A/Fibers_large_Cy3.png]

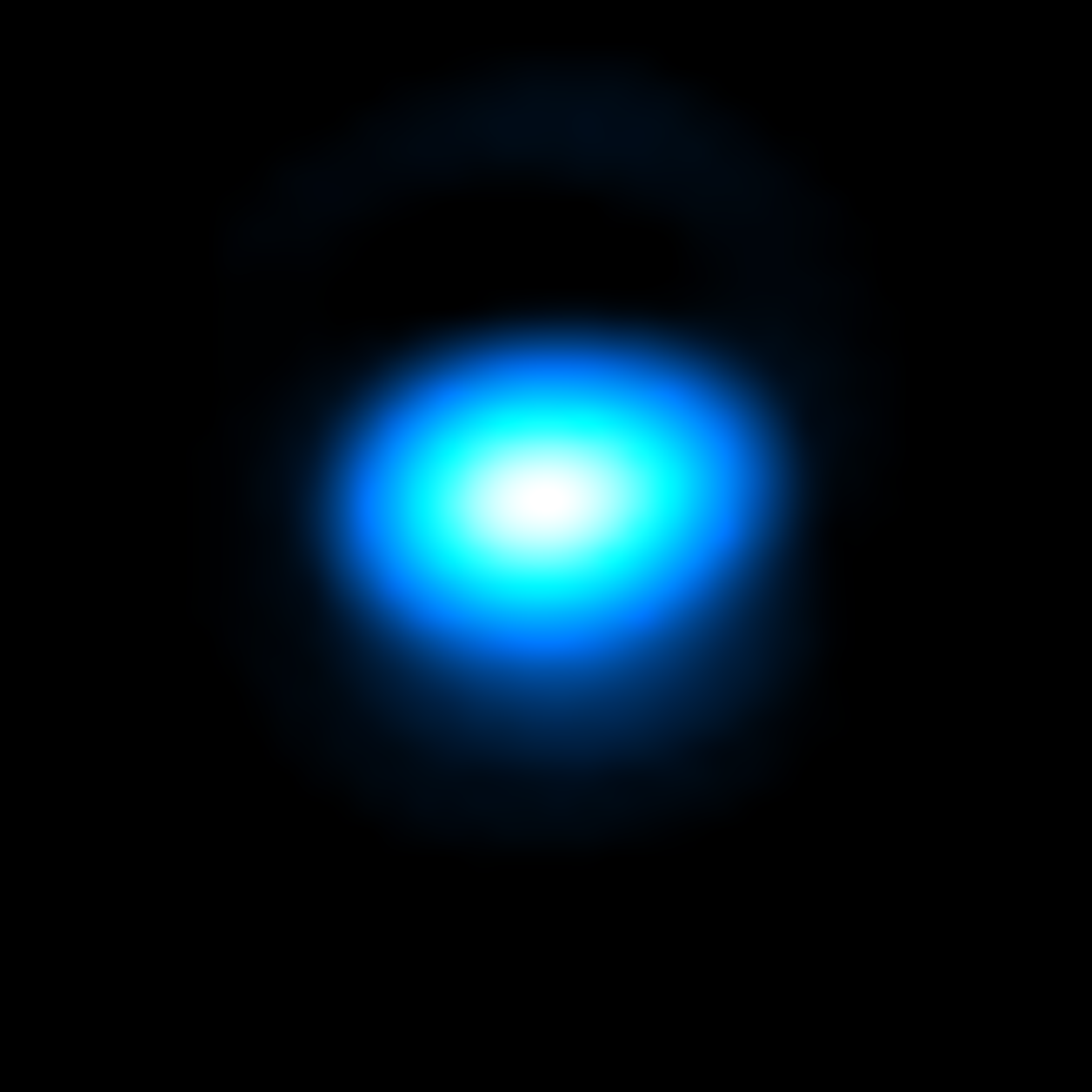

Supplement: Supplementary file 6 — Source data Fig. 3 [file 44318_2025_574_MOESM6_ESM.zip › Figure 3/3C/insets/Fig3c_MAX_inset1_Cy3.png]

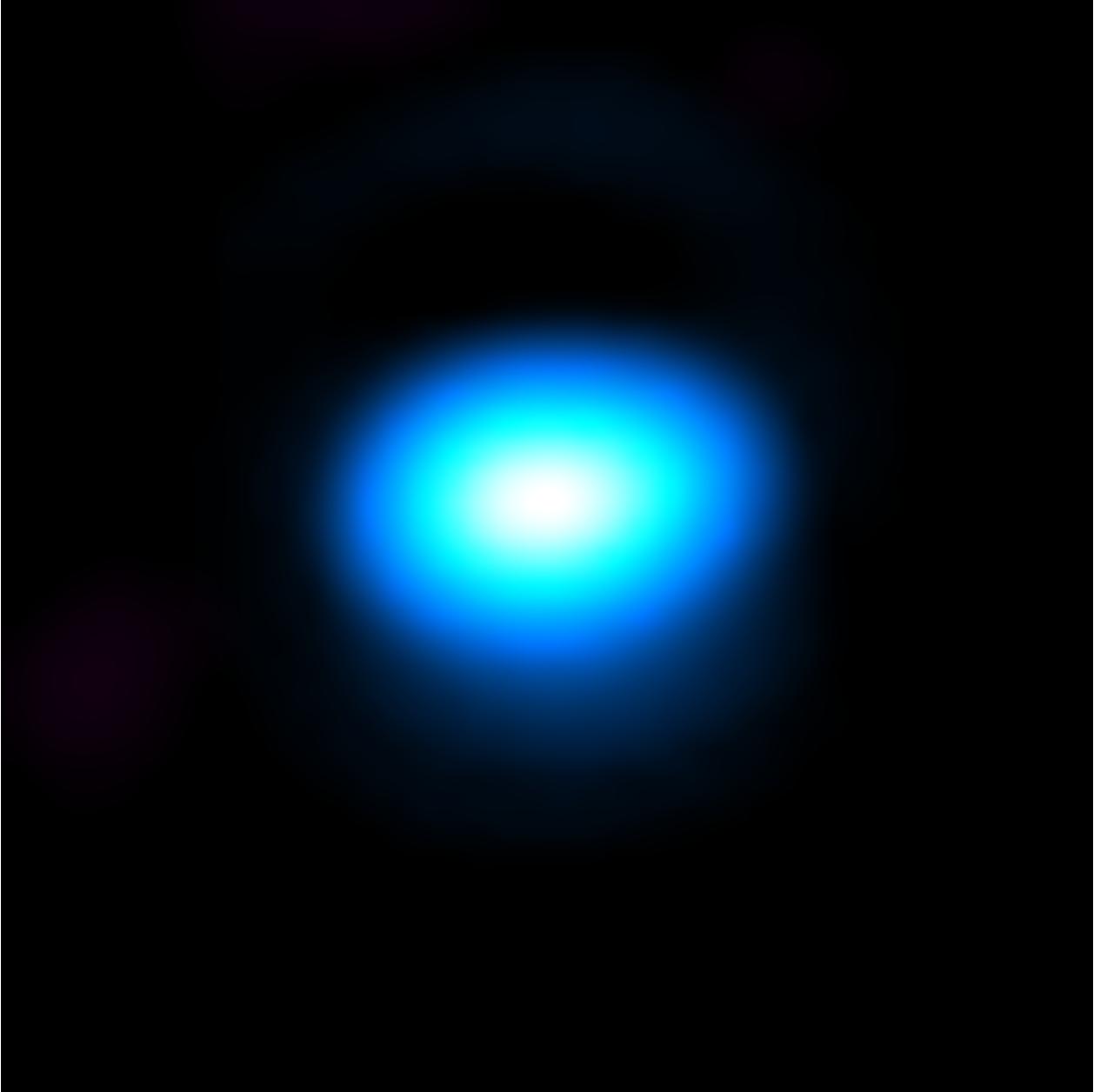

Supplement: Supplementary file 6 — Source data Fig. 3 [file 44318_2025_574_MOESM6_ESM.zip › Figure 3/3C/insets/Fig3c_MAX_inset1_EdU_Cy3.png]

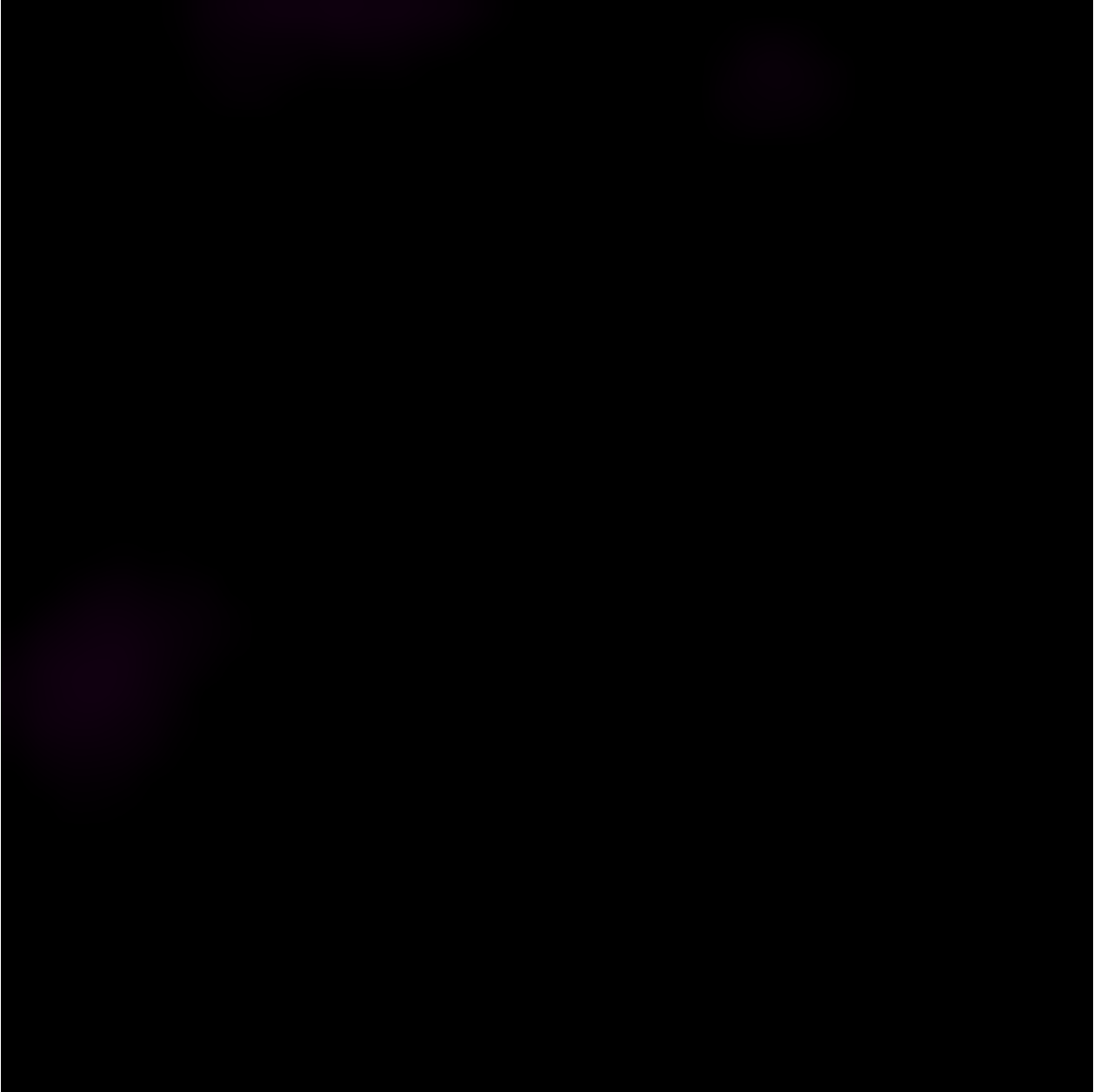

Supplement: Supplementary file 6 — Source data Fig. 3 [file 44318_2025_574_MOESM6_ESM.zip › Figure 3/3C/insets/Fig3c_MAX_inset1_EdU.png]

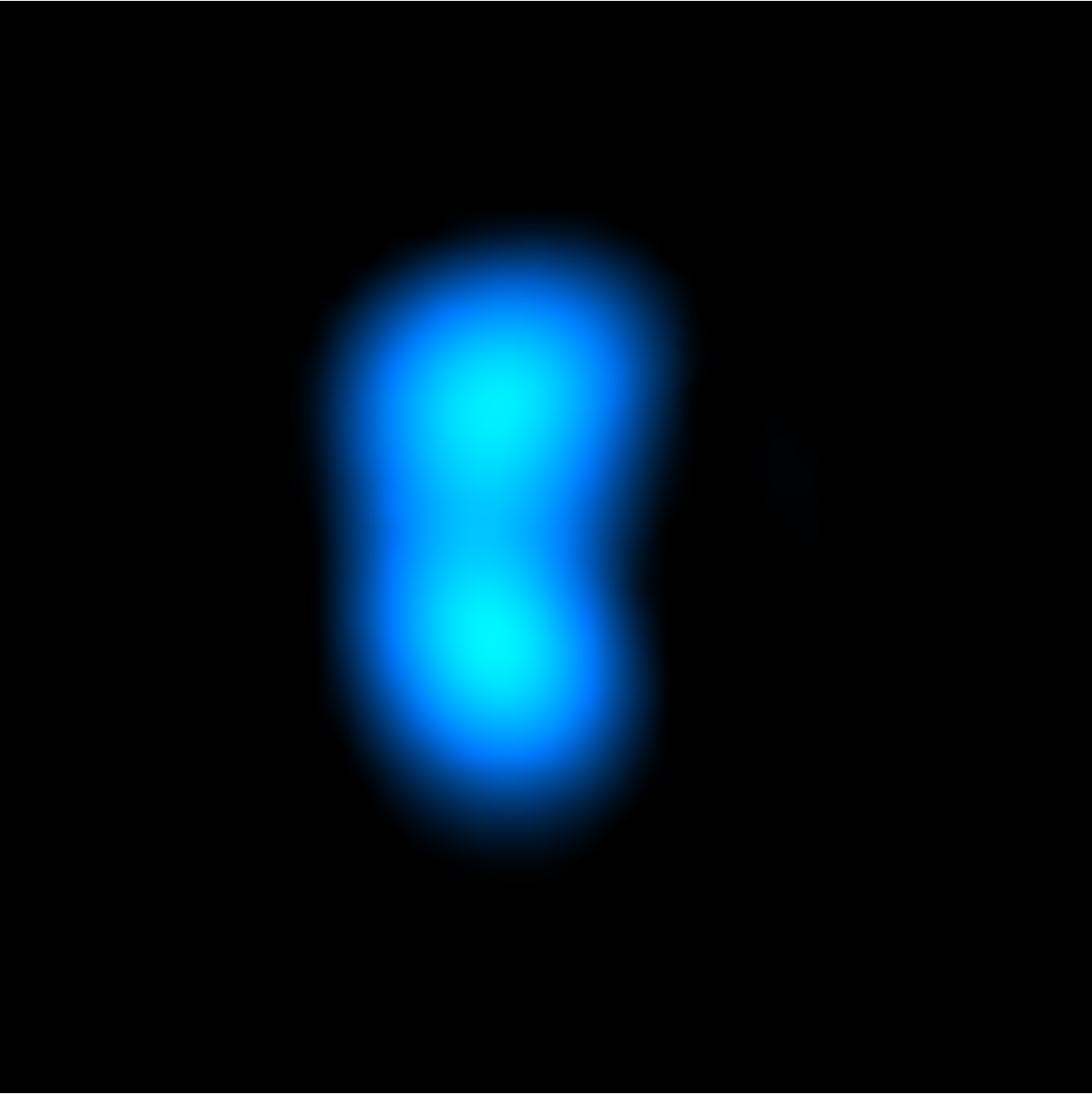

Supplement: Supplementary file 6 — Source data Fig. 3 [file 44318_2025_574_MOESM6_ESM.zip › Figure 3/3C/insets/Fig3c_MAX_inset2_Cy3.png]

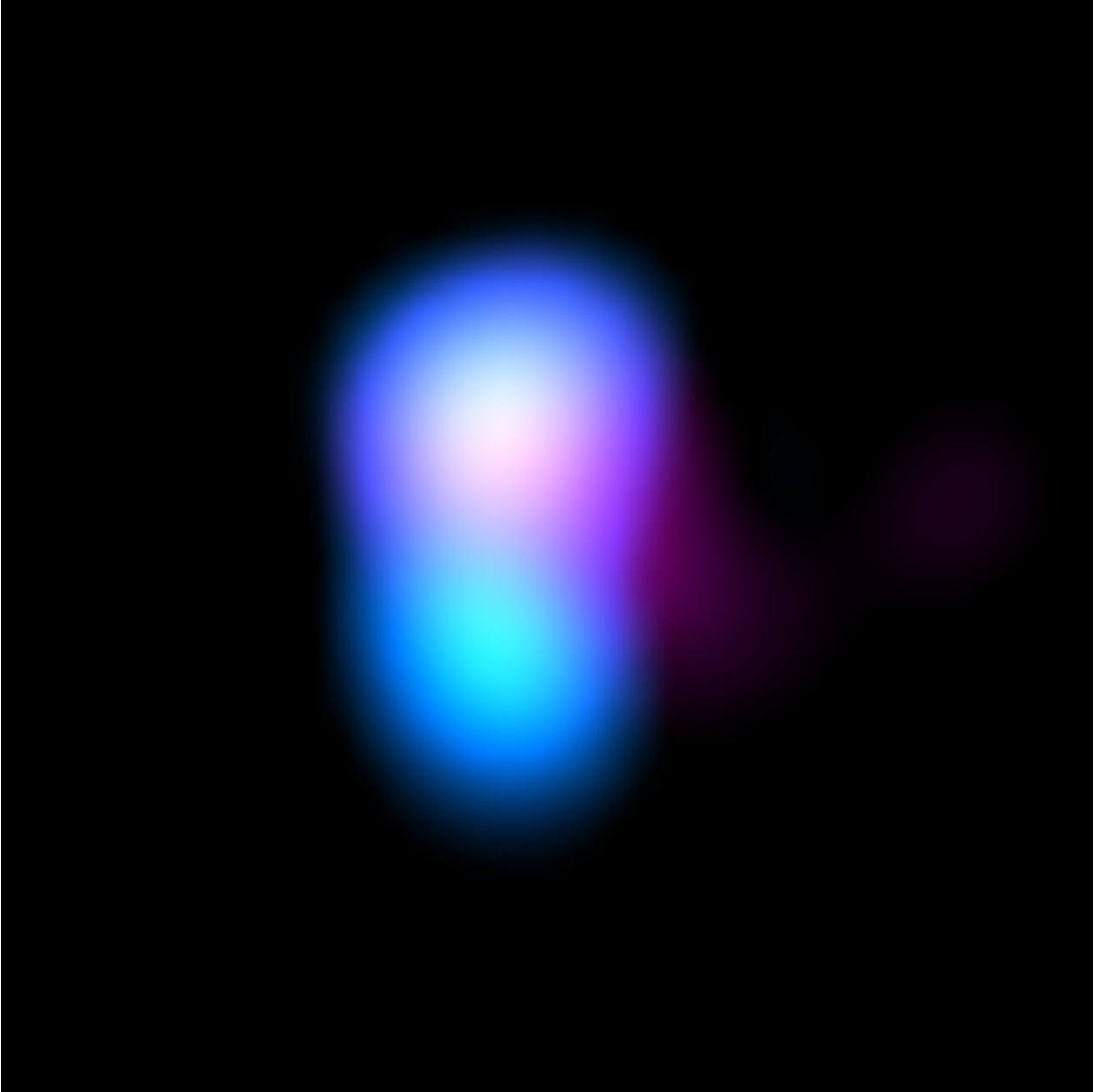

Supplement: Supplementary file 6 — Source data Fig. 3 [file 44318_2025_574_MOESM6_ESM.zip › Figure 3/3C/insets/Fig3c_MAX_inset2_EdU_Cy3.png]

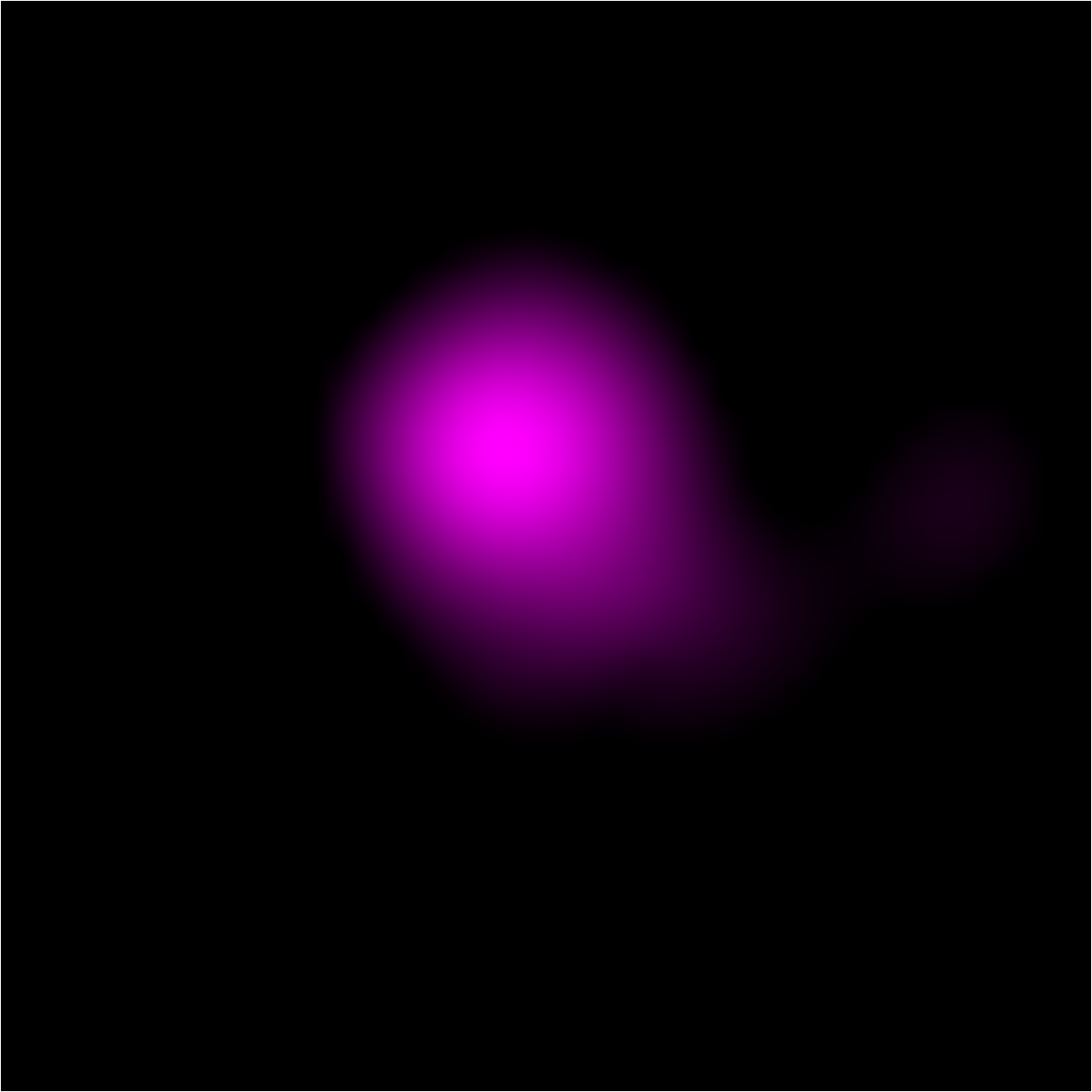

Supplement: Supplementary file 6 — Source data Fig. 3 [file 44318_2025_574_MOESM6_ESM.zip › Figure 3/3C/insets/Fig3c_MAX_inset2_EdU.png]

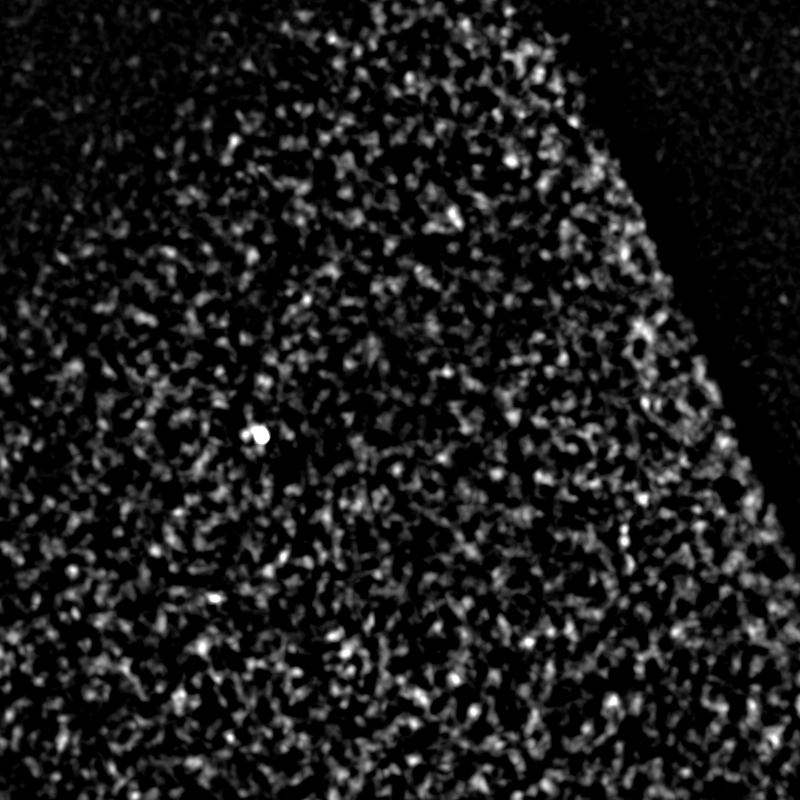

Supplement: Supplementary file 6 — Source data Fig. 3 [file 44318_2025_574_MOESM6_ESM.zip › Figure 3/3C/insets/Fig3c_MAX_cell.tif]

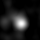

Supplement: Supplementary file 6 — Source data Fig. 3 [file 44318_2025_574_MOESM6_ESM.zip › Figure 3/3C/insets/Fig3c_MAX_inset1.tif]

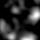

Supplement: Supplementary file 6 — Source data Fig. 3 [file 44318_2025_574_MOESM6_ESM.zip › Figure 3/3C/insets/Fig3c_MAX_inset2.tif]

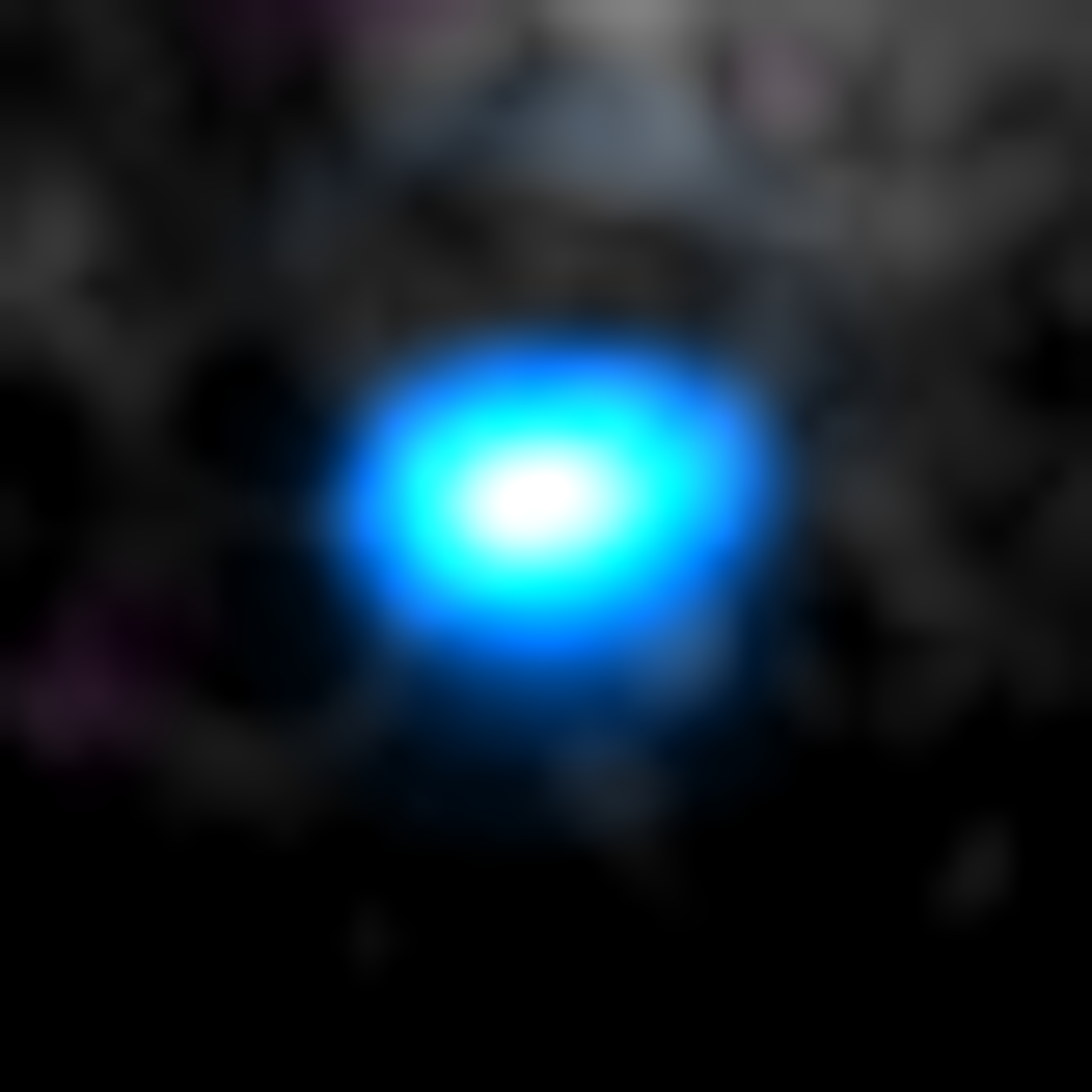

Supplement: Supplementary file 6 — Source data Fig. 3 [file 44318_2025_574_MOESM6_ESM.zip › Figure 3/3C/insets/Fig3c_MAX_inset1_EdU_cy3_DAPI.png]

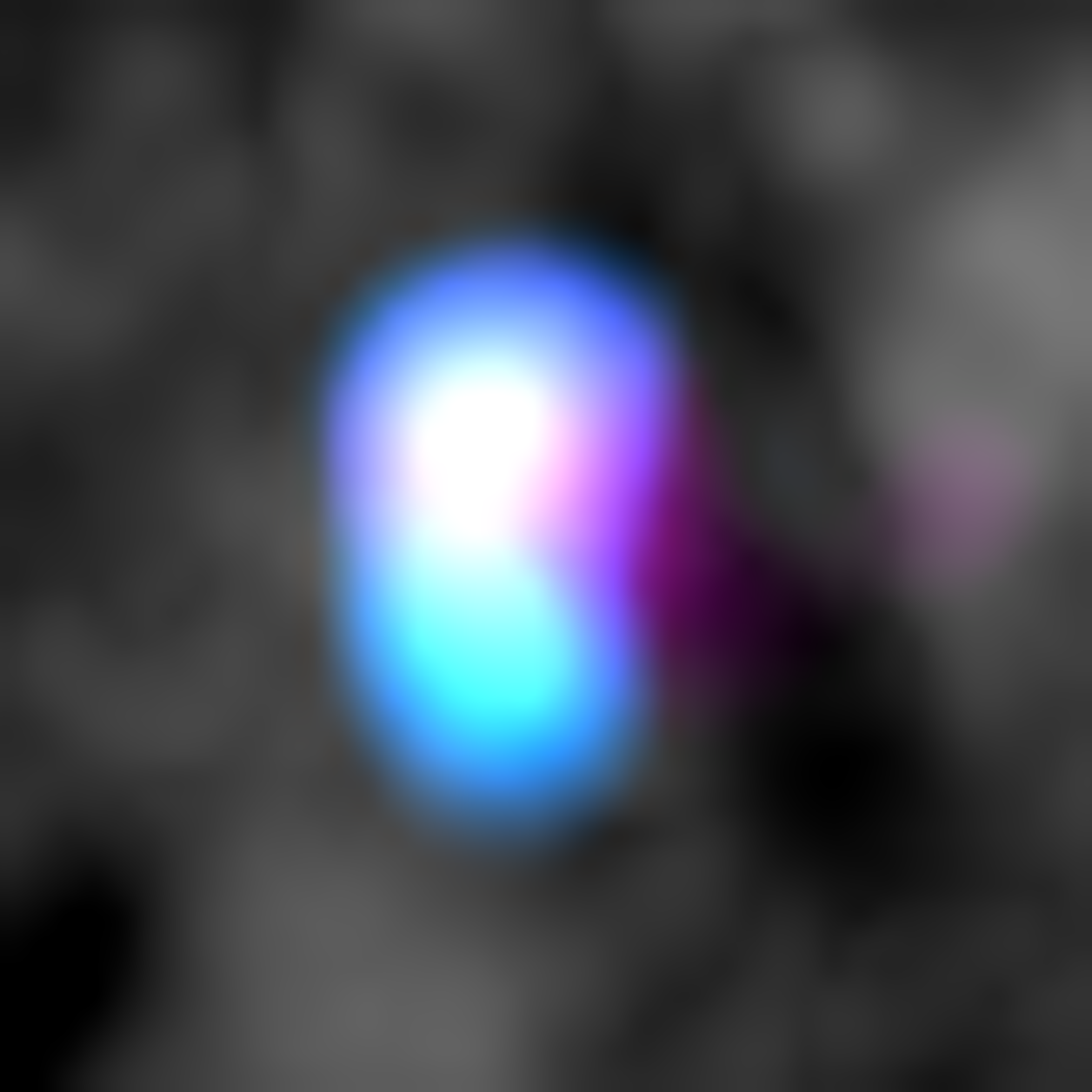

Supplement: Supplementary file 6 — Source data Fig. 3 [file 44318_2025_574_MOESM6_ESM.zip › Figure 3/3C/insets/Fig3c_MAX_inset2_EdU_Cy3_DAPI.png]

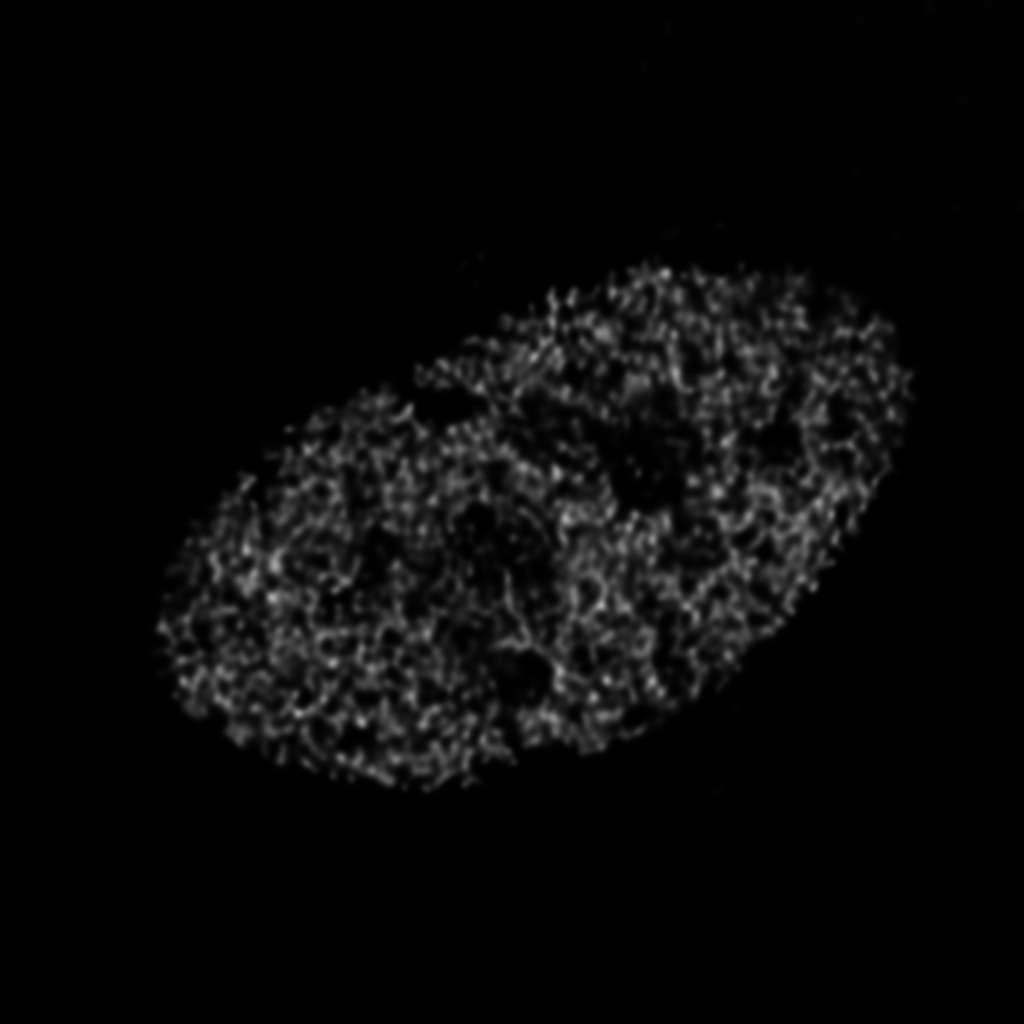

Supplement: Supplementary file 7 — Source data Fig. 4 [file 44318_2025_574_MOESM7_ESM.zip › Figure 4/4A/Control/Image_3.png]

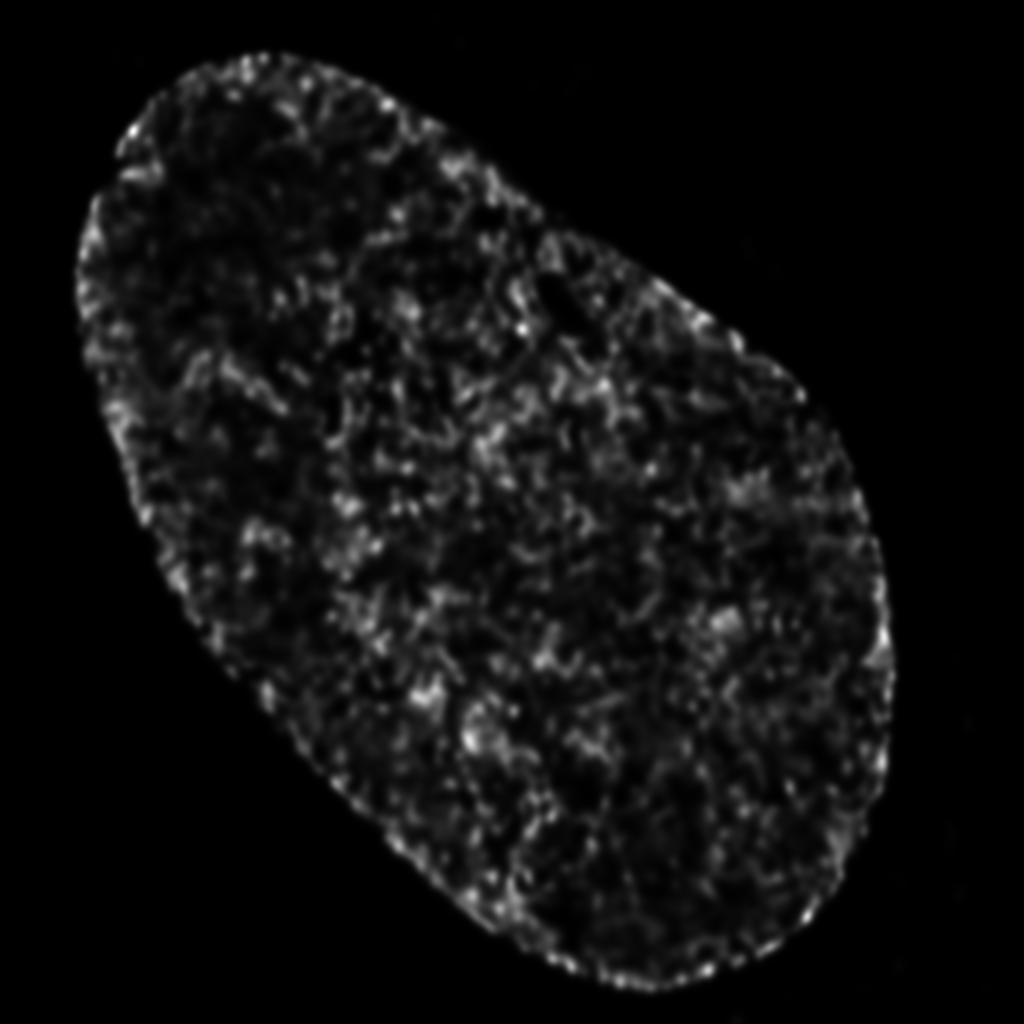

Supplement: Supplementary file 7 — Source data Fig. 4 [file 44318_2025_574_MOESM7_ESM.zip › Figure 4/4A/Control/Image_14.png]

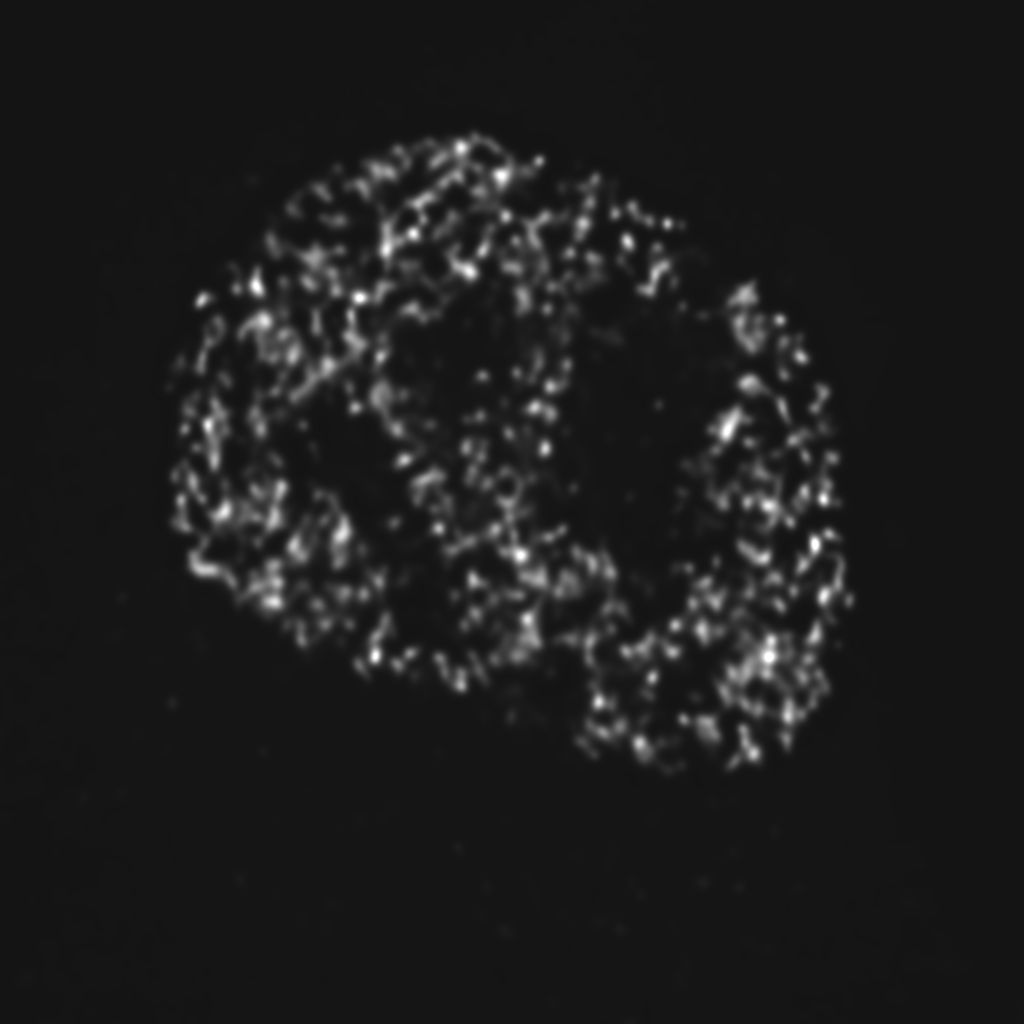

Supplement: Supplementary file 7 — Source data Fig. 4 [file 44318_2025_574_MOESM7_ESM.zip › Figure 4/4A/Control/Image_15.png]

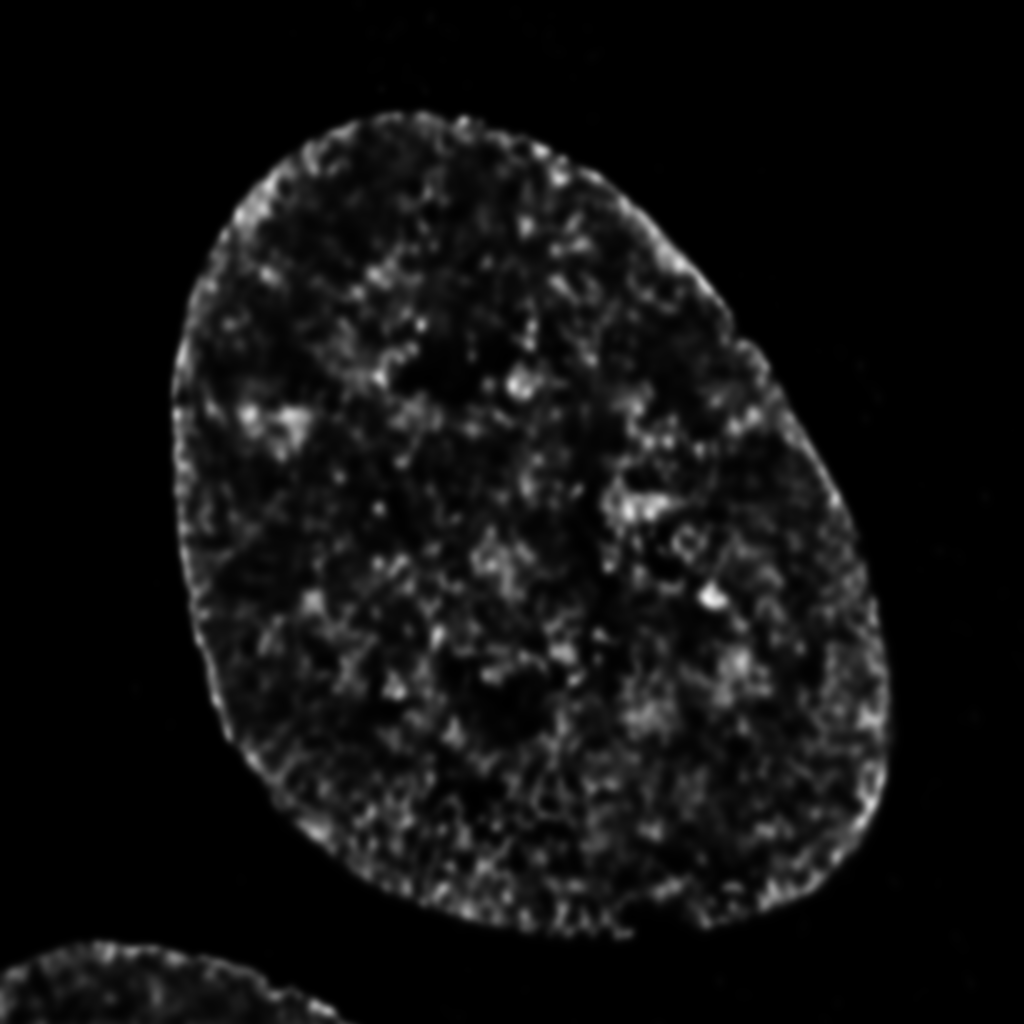

Supplement: Supplementary file 7 — Source data Fig. 4 [file 44318_2025_574_MOESM7_ESM.zip › Figure 4/4A/Control/Image_16.png]

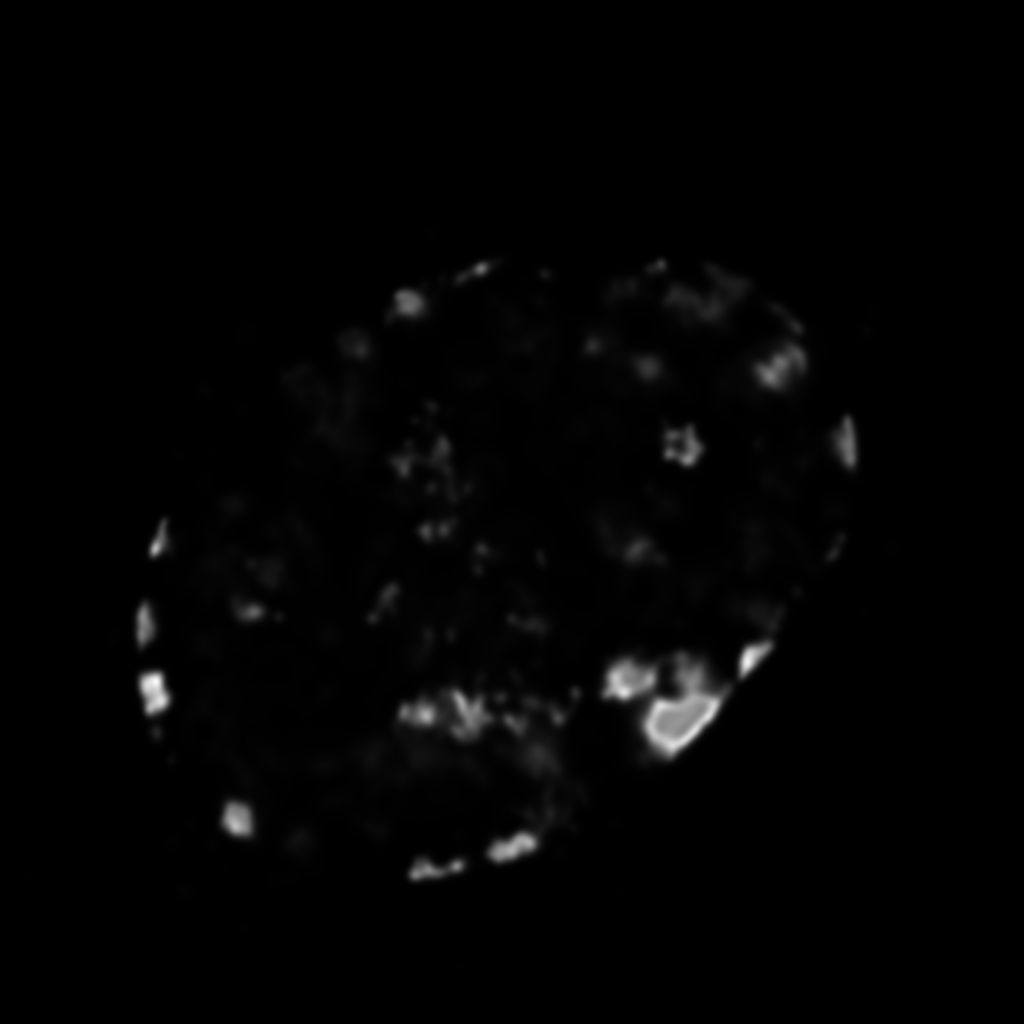

Supplement: Supplementary file 7 — Source data Fig. 4 [file 44318_2025_574_MOESM7_ESM.zip › Figure 4/4A/Control/Image_23.png]

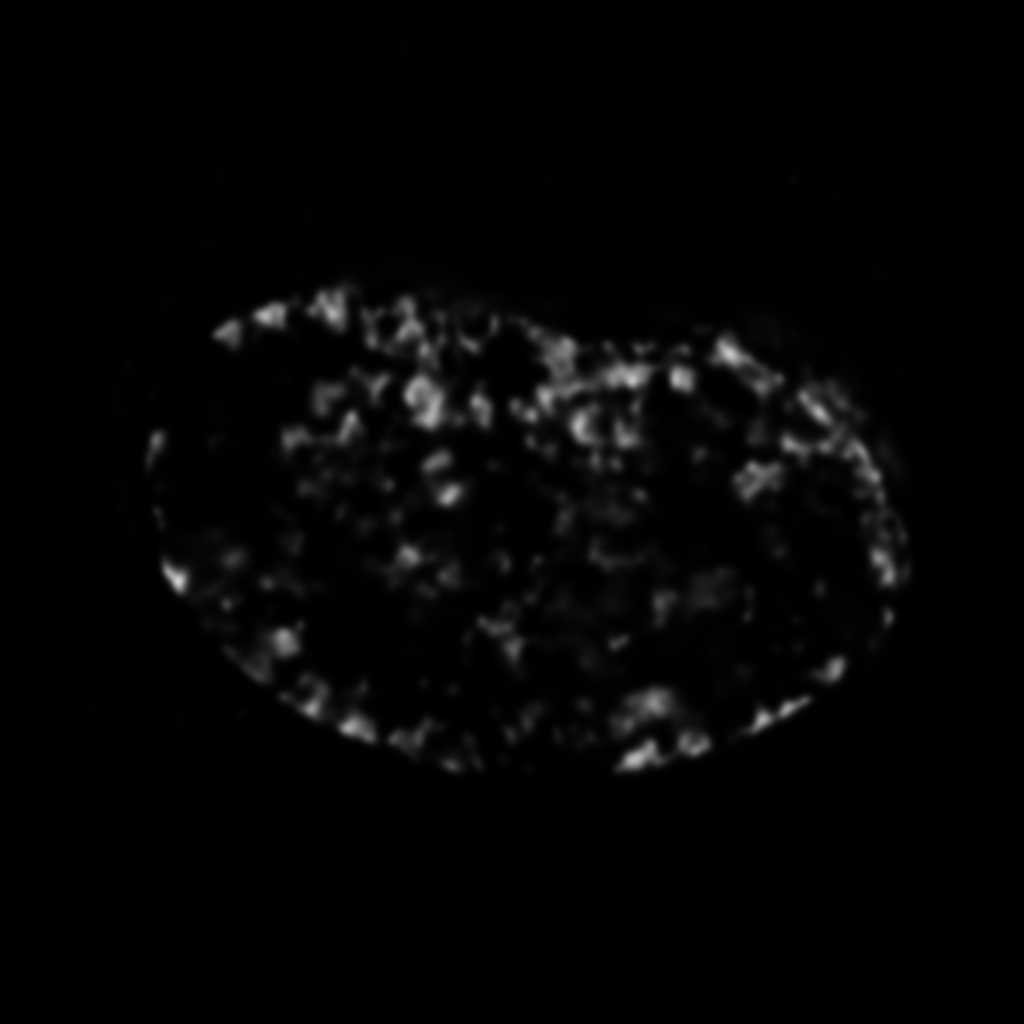

Supplement: Supplementary file 7 — Source data Fig. 4 [file 44318_2025_574_MOESM7_ESM.zip › Figure 4/4A/Control/Image_24.png]

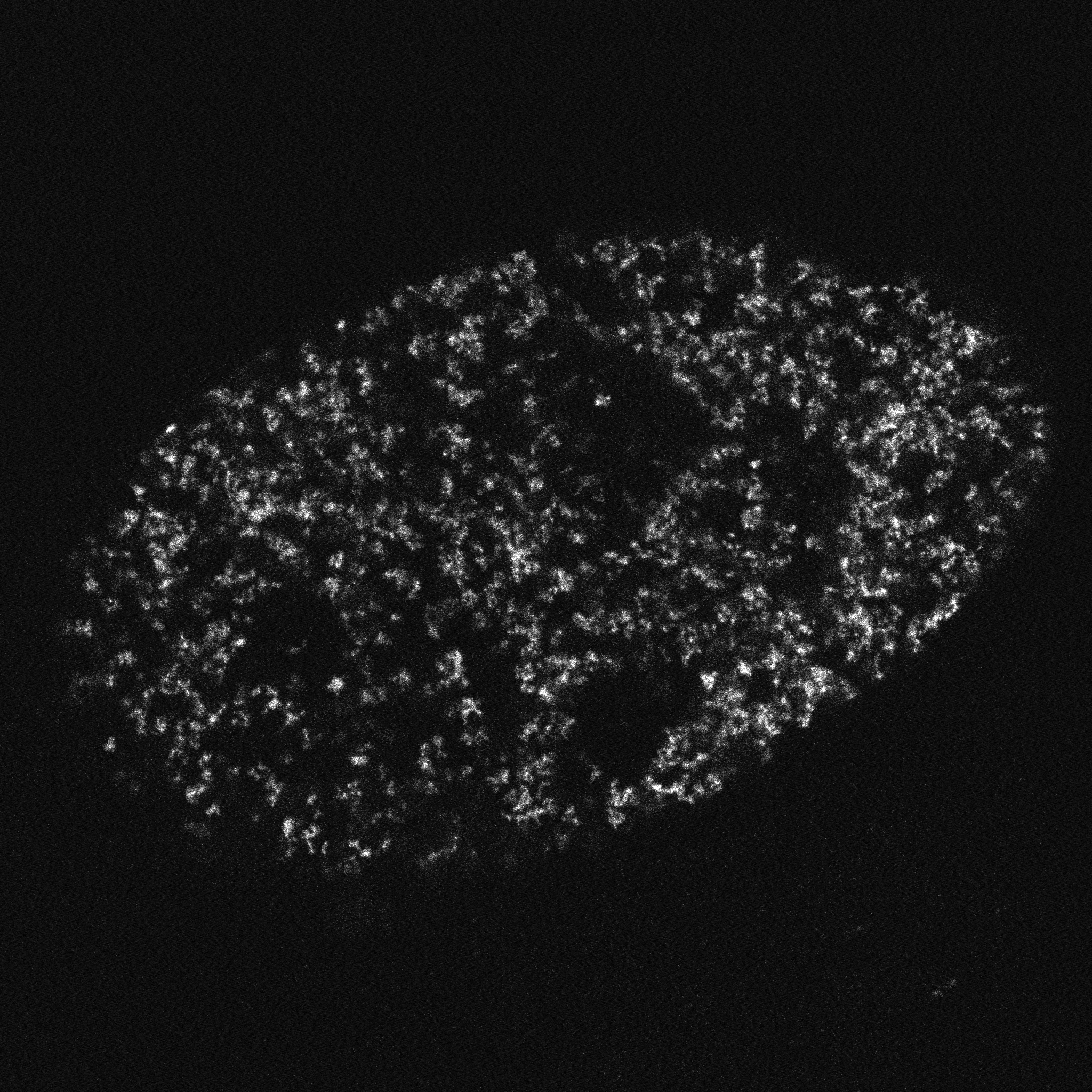

Supplement: Supplementary file 7 — Source data Fig. 4 [file 44318_2025_574_MOESM7_ESM.zip › Figure 4/4A/ExM/Image 16.tif]

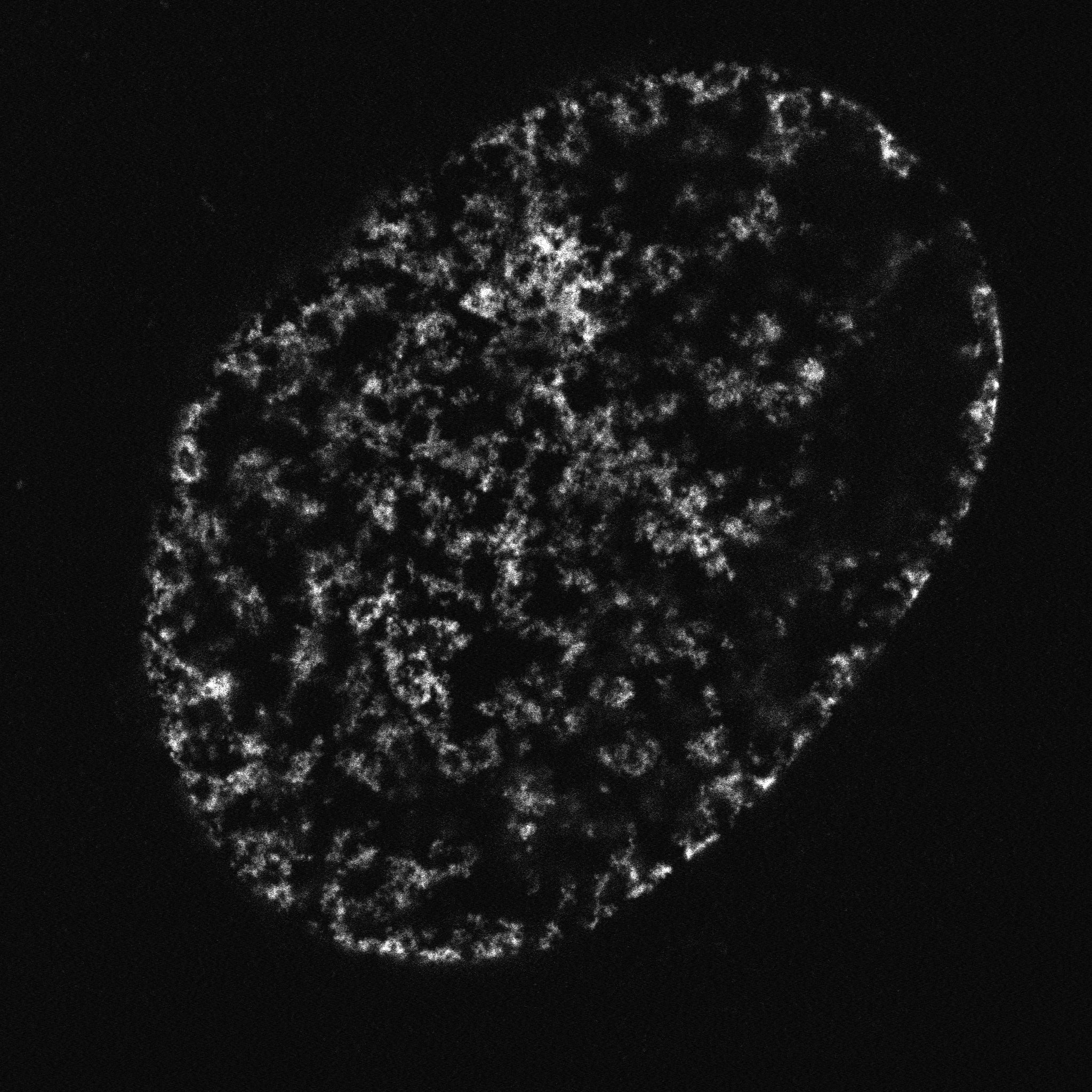

Supplement: Supplementary file 7 — Source data Fig. 4 [file 44318_2025_574_MOESM7_ESM.zip › Figure 4/4A/ExM/Image 22.tif]

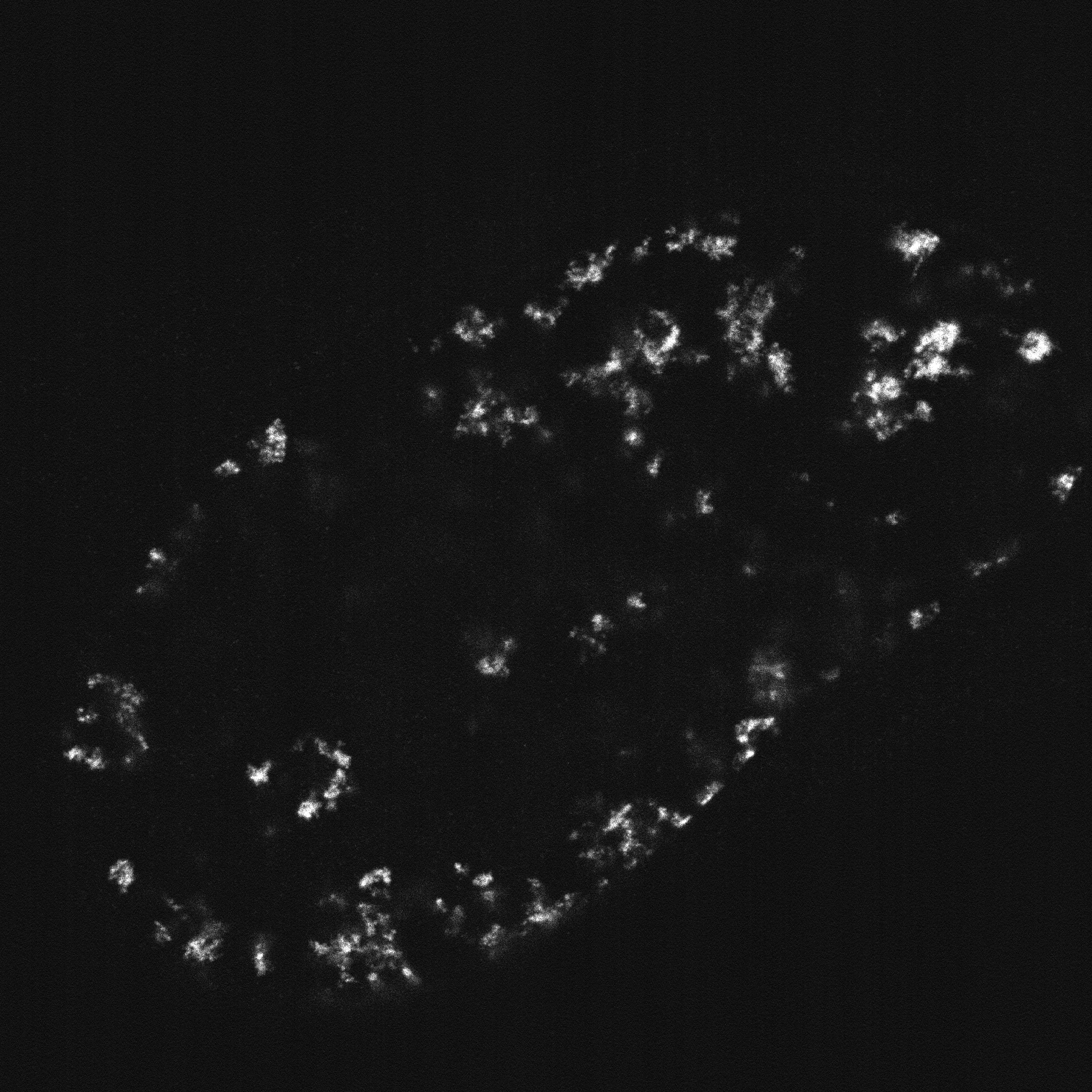

Supplement: Supplementary file 7 — Source data Fig. 4 [file 44318_2025_574_MOESM7_ESM.zip › Figure 4/4A/ExM/Image 25.tif]

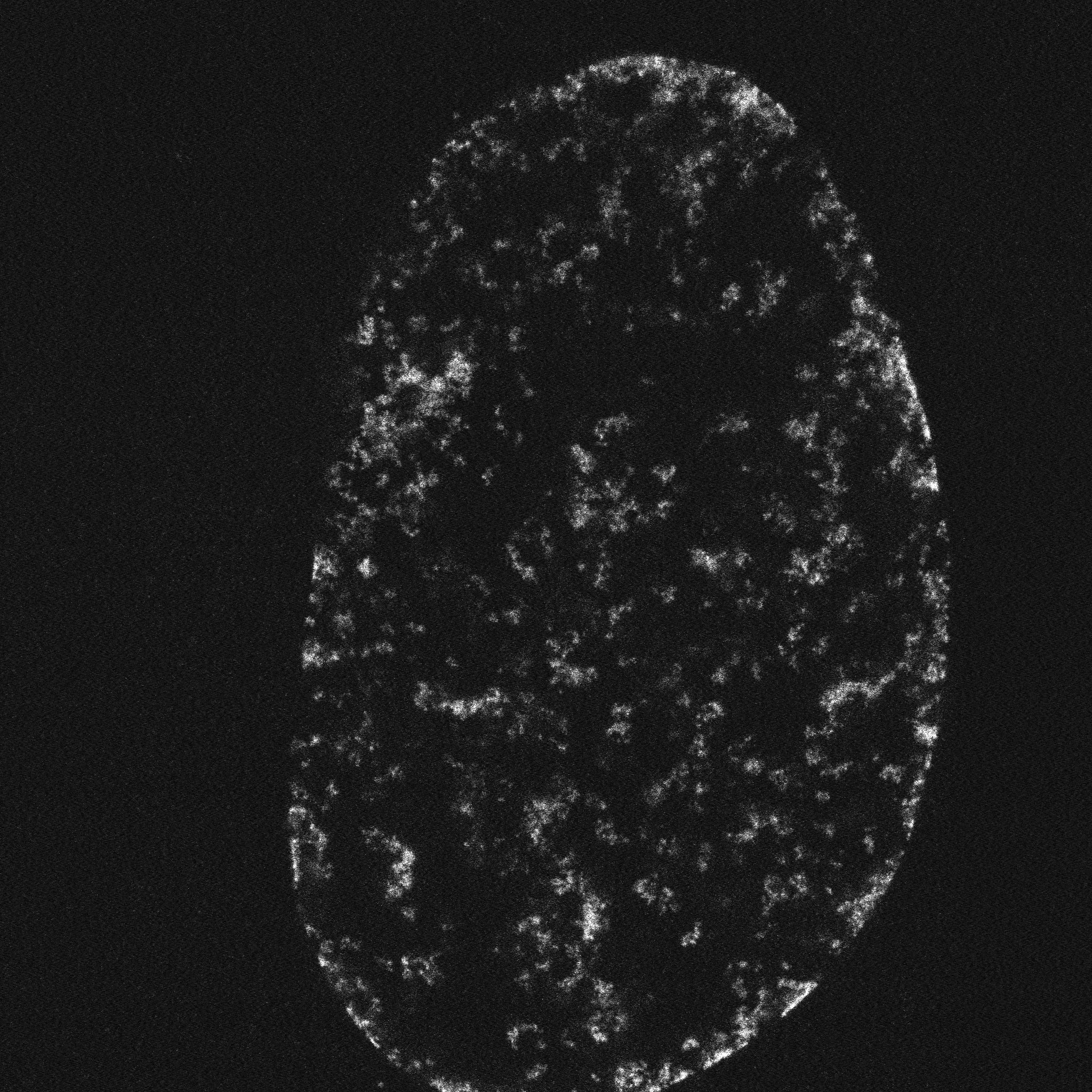

Supplement: Supplementary file 7 — Source data Fig. 4 [file 44318_2025_574_MOESM7_ESM.zip › Figure 4/4A/ExM/Image 27.tif]

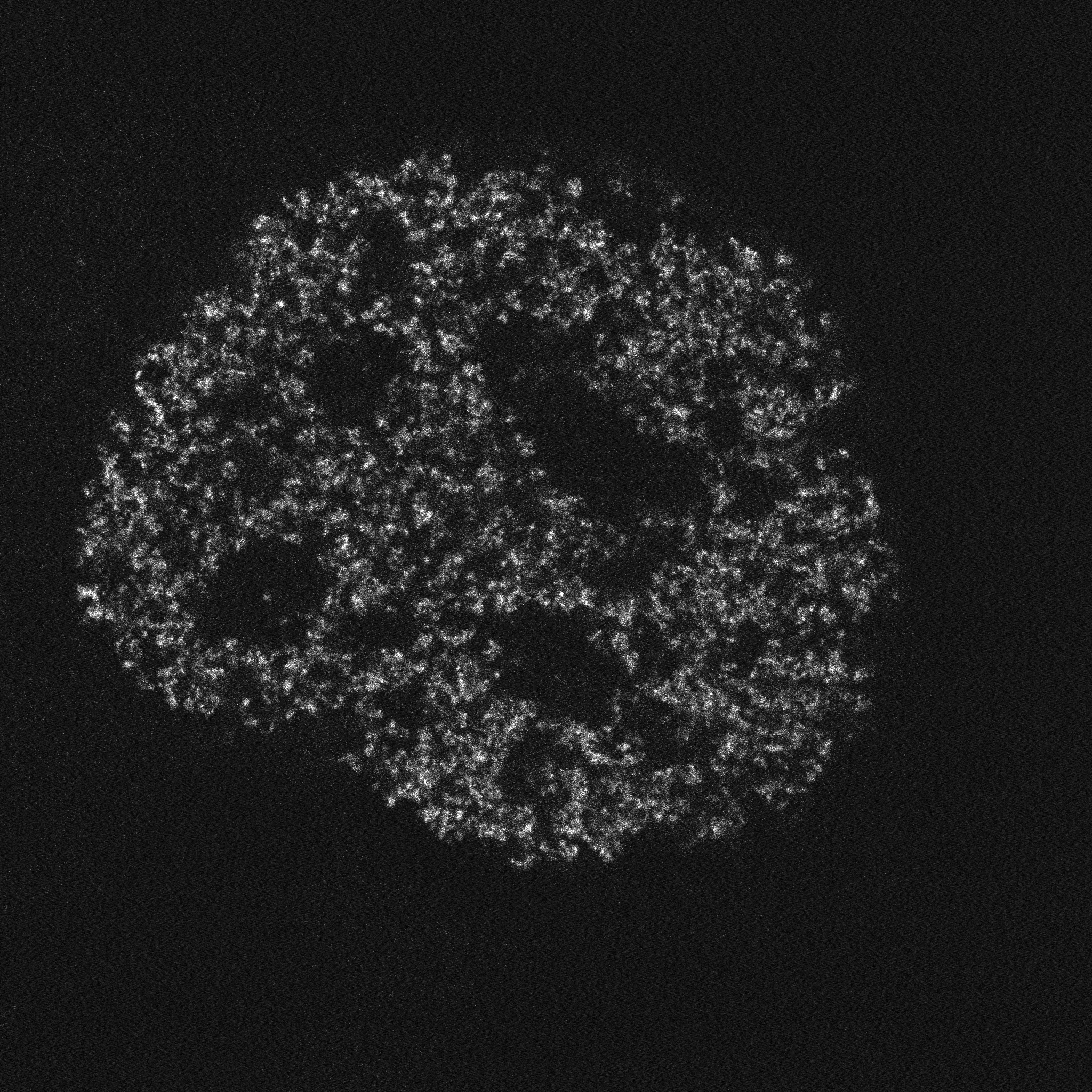

Supplement: Supplementary file 7 — Source data Fig. 4 [file 44318_2025_574_MOESM7_ESM.zip › Figure 4/4A/ExM/Image 28.tif]

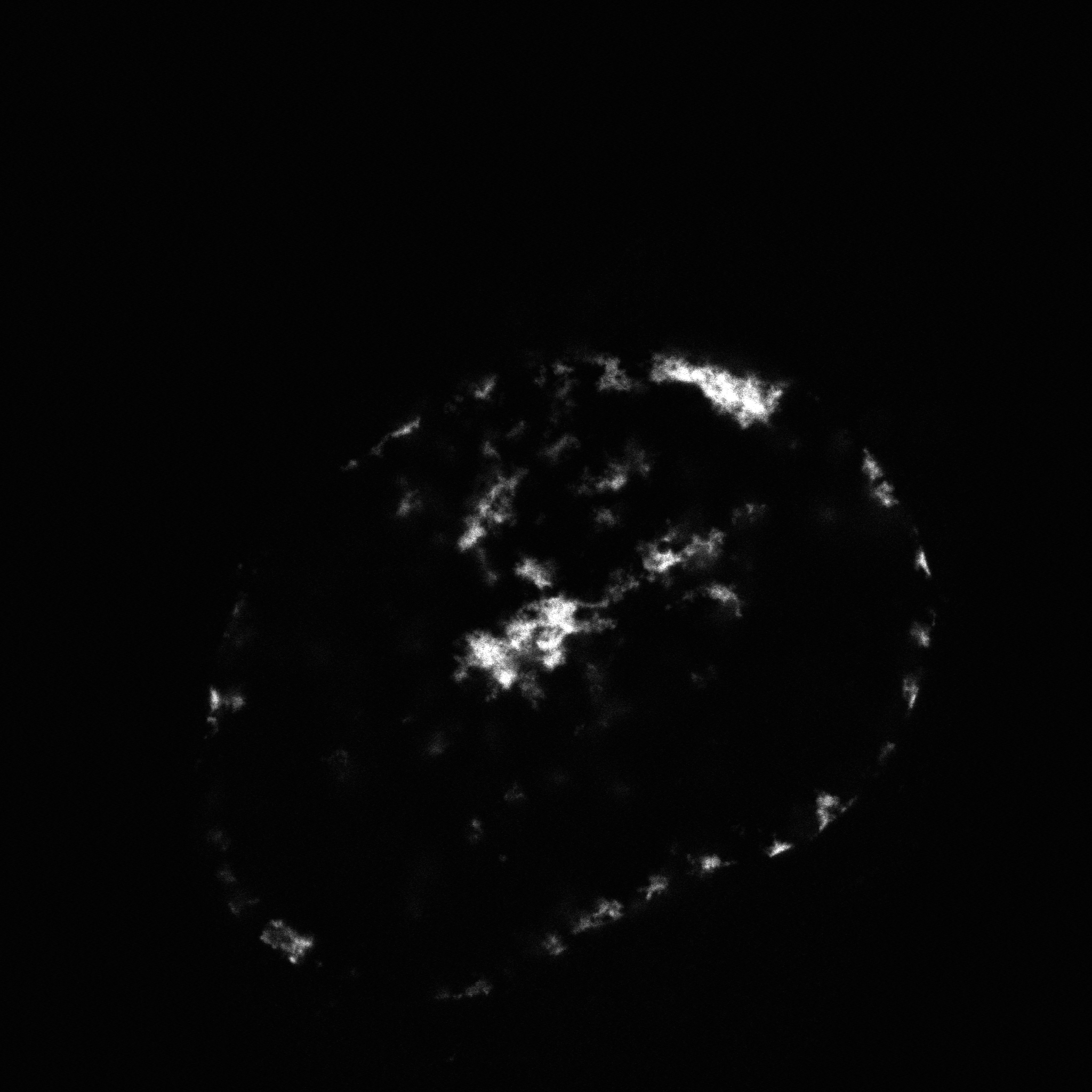

Supplement: Supplementary file 7 — Source data Fig. 4 [file 44318_2025_574_MOESM7_ESM.zip › Figure 4/4A/ExM/Image 38.tif]

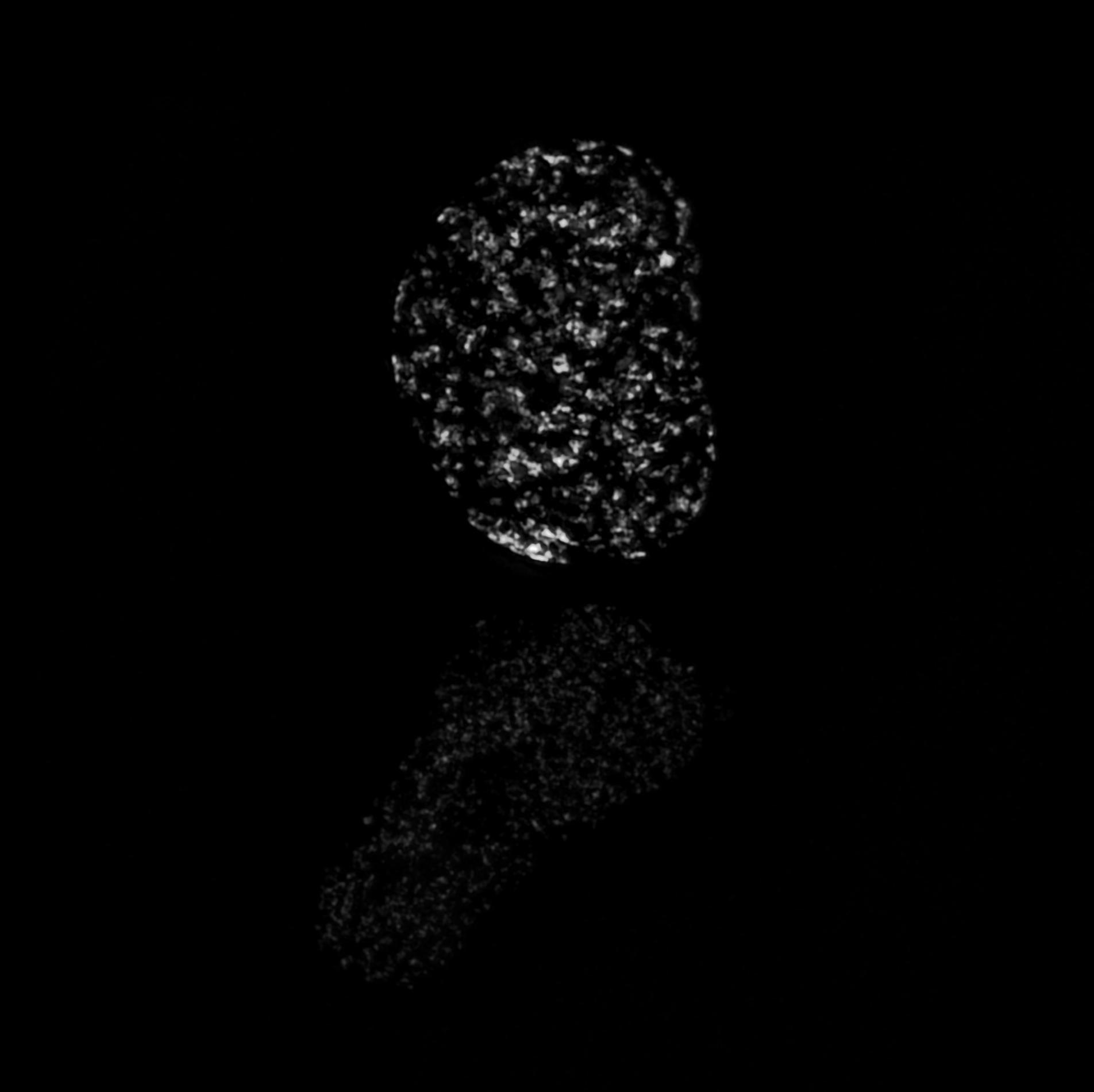

Supplement: Supplementary file 8 — Source data Fig. 5 [file 44318_2025_574_MOESM8_ESM.zip › Figure 5/5A/MAX_mid_Image 87.tif]

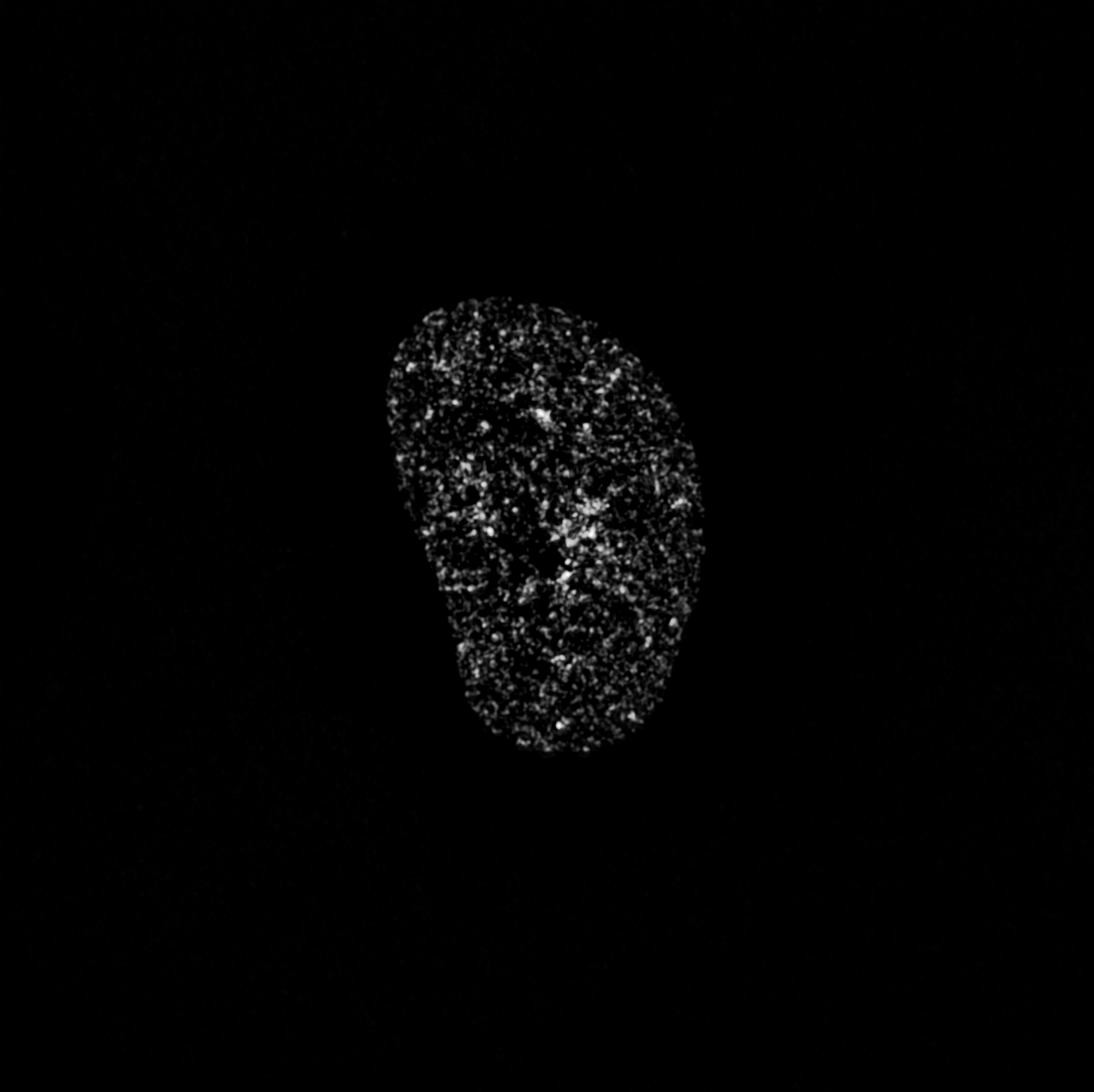

Supplement: Supplementary file 8 — Source data Fig. 5 [file 44318_2025_574_MOESM8_ESM.zip › Figure 5/5A/MAX_early_Image 89.tif]

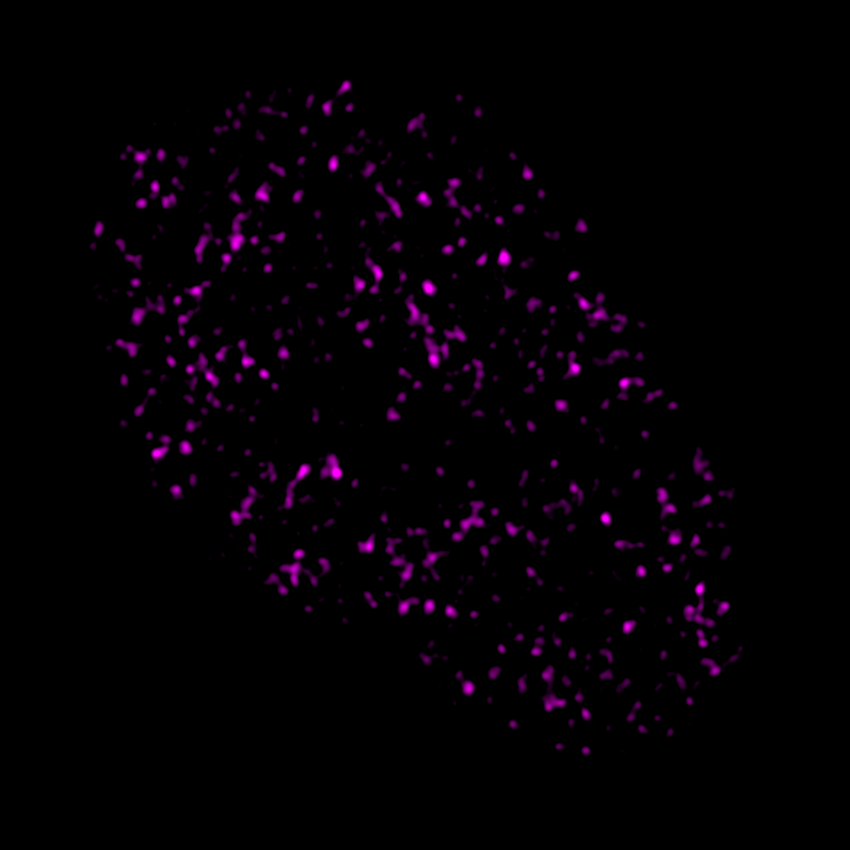

Supplement: Supplementary file 8 — Source data Fig. 5 [file 44318_2025_574_MOESM8_ESM.zip › Figure 5/5F/EdU_nucleus4.png]

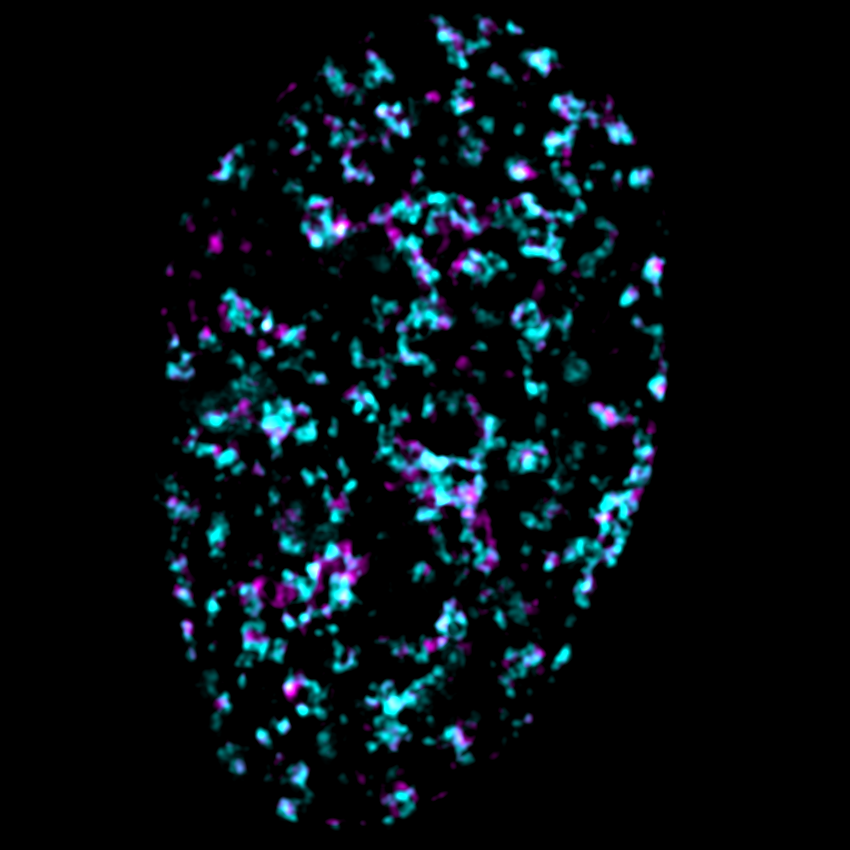

Supplement: Supplementary file 8 — Source data Fig. 5 [file 44318_2025_574_MOESM8_ESM.zip › Figure 5/5F/EdU_dUTP_nucleus5.png]

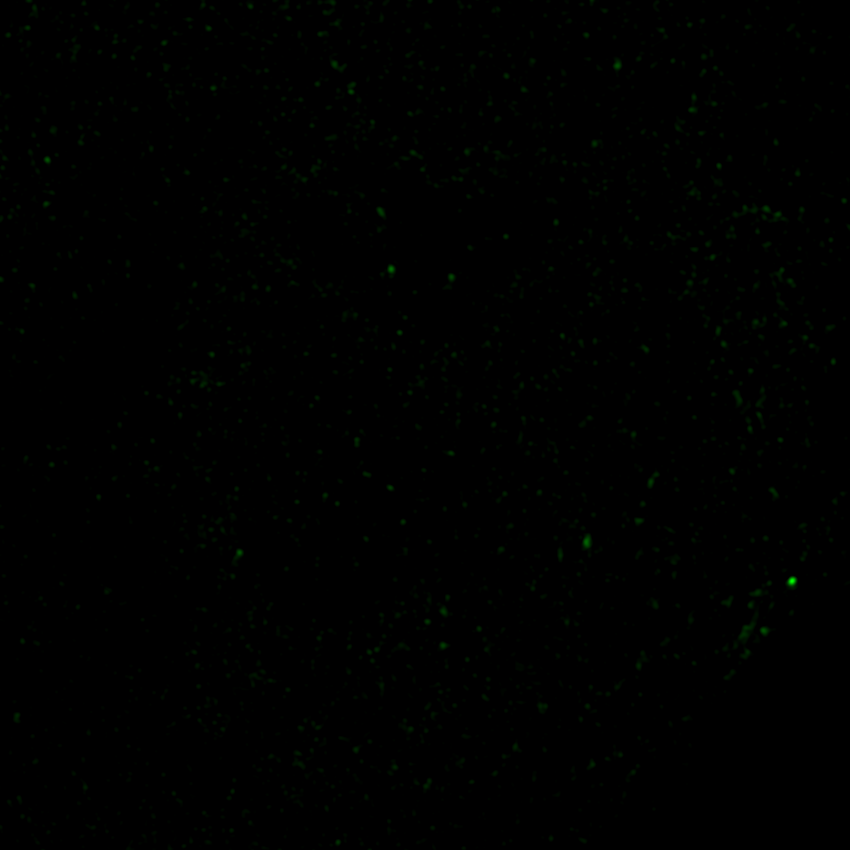

Supplement: Supplementary file 8 — Source data Fig. 5 [file 44318_2025_574_MOESM8_ESM.zip › Figure 5/5F/pRPA_nucleus5.png]

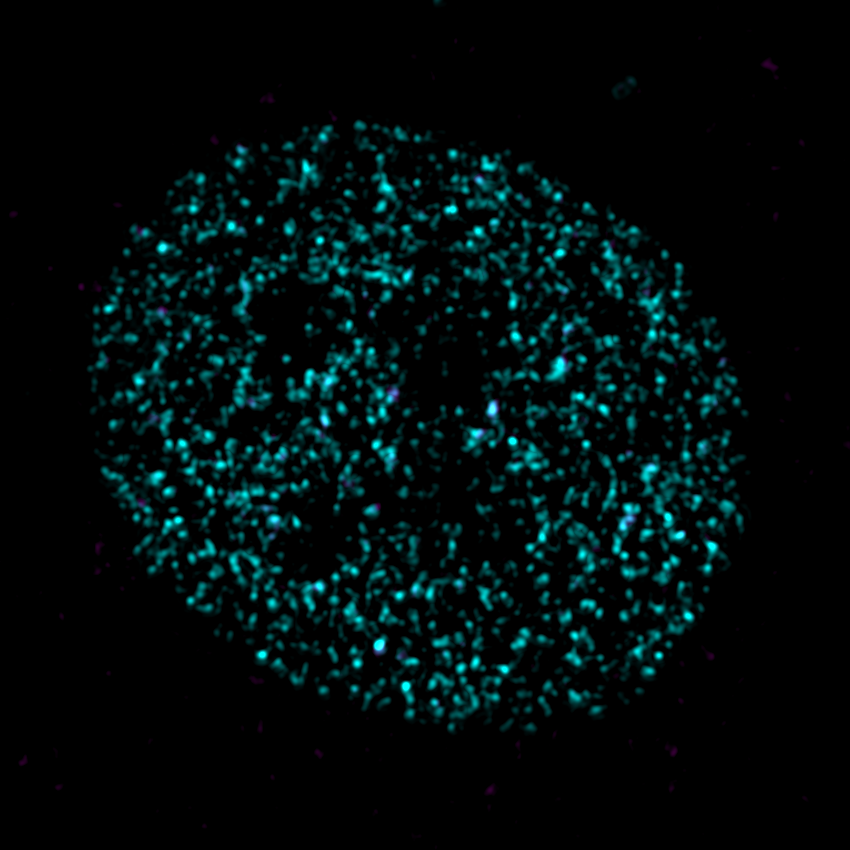

Supplement: Supplementary file 8 — Source data Fig. 5 [file 44318_2025_574_MOESM8_ESM.zip › Figure 5/5F/EdU_dUTP_HU_nucleus6.png]

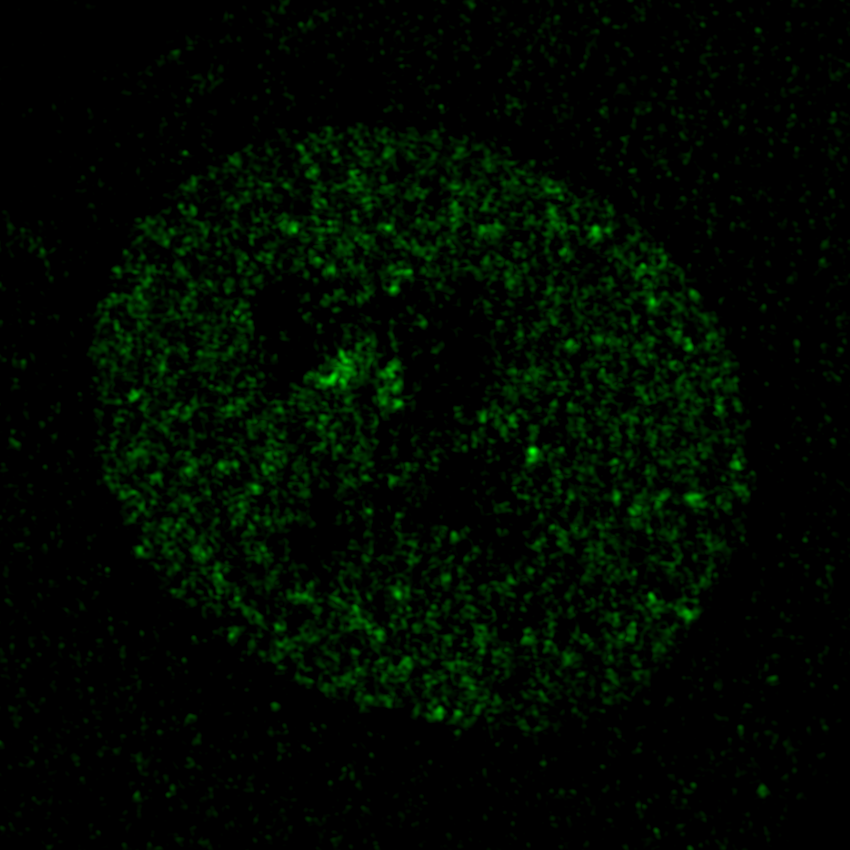

Supplement: Supplementary file 8 — Source data Fig. 5 [file 44318_2025_574_MOESM8_ESM.zip › Figure 5/5F/pRPA_nucleus6.png]

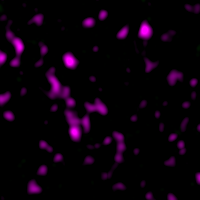

Supplement: Supplementary file 8 — Source data Fig. 5 [file 44318_2025_574_MOESM8_ESM.zip › Figure 5/5F/crop_merge_nucleus4.png]

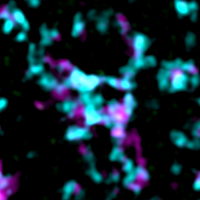

Supplement: Supplementary file 8 — Source data Fig. 5 [file 44318_2025_574_MOESM8_ESM.zip › Figure 5/5F/crop_merge_nucleus5.png]

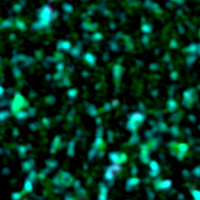

Supplement: Supplementary file 8 — Source data Fig. 5 [file 44318_2025_574_MOESM8_ESM.zip › Figure 5/5F/crop_merge_nucleus6.png]

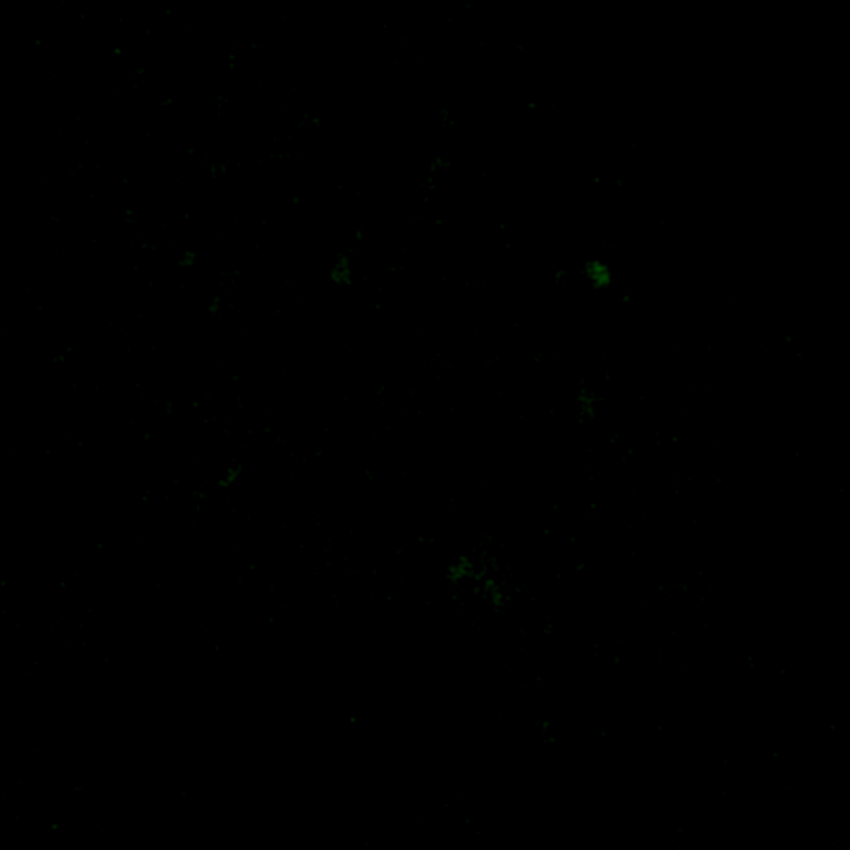

Supplement: Supplementary file 8 — Source data Fig. 5 [file 44318_2025_574_MOESM8_ESM.zip › Figure 5/5F/pRPA_nucleus4.png]

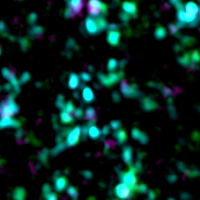

Supplement: Supplementary file 8 — Source data Fig. 5 [file 44318_2025_574_MOESM8_ESM.zip › Figure 5/5E/crop_merge_nucleus3.png]

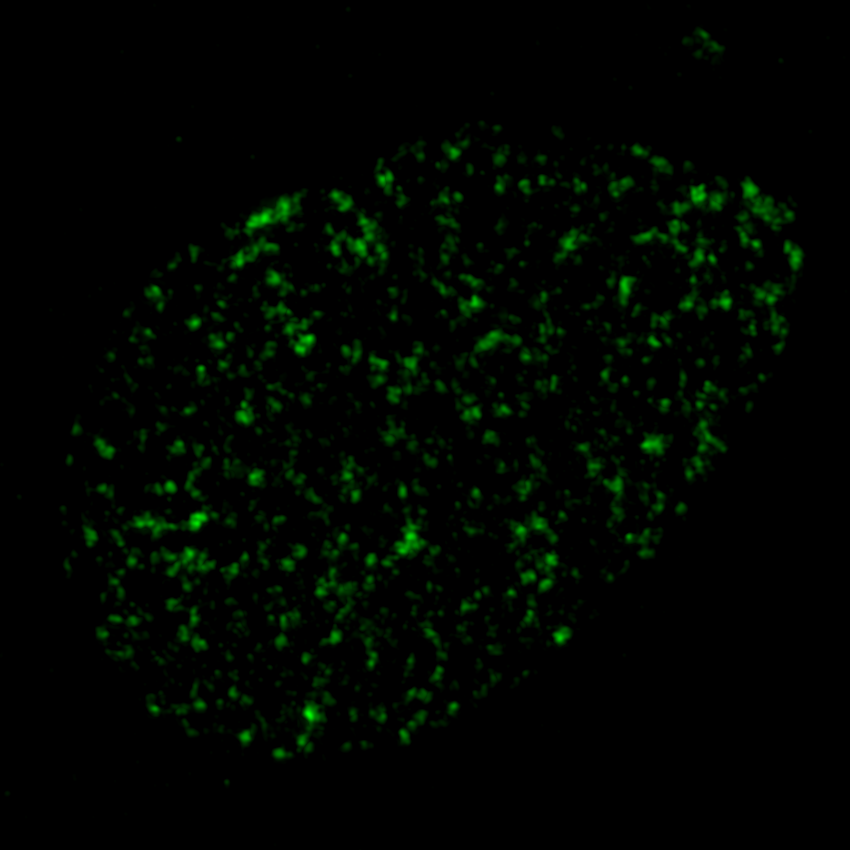

Supplement: Supplementary file 8 — Source data Fig. 5 [file 44318_2025_574_MOESM8_ESM.zip › Figure 5/5E/H2AX_nucleus3.png]

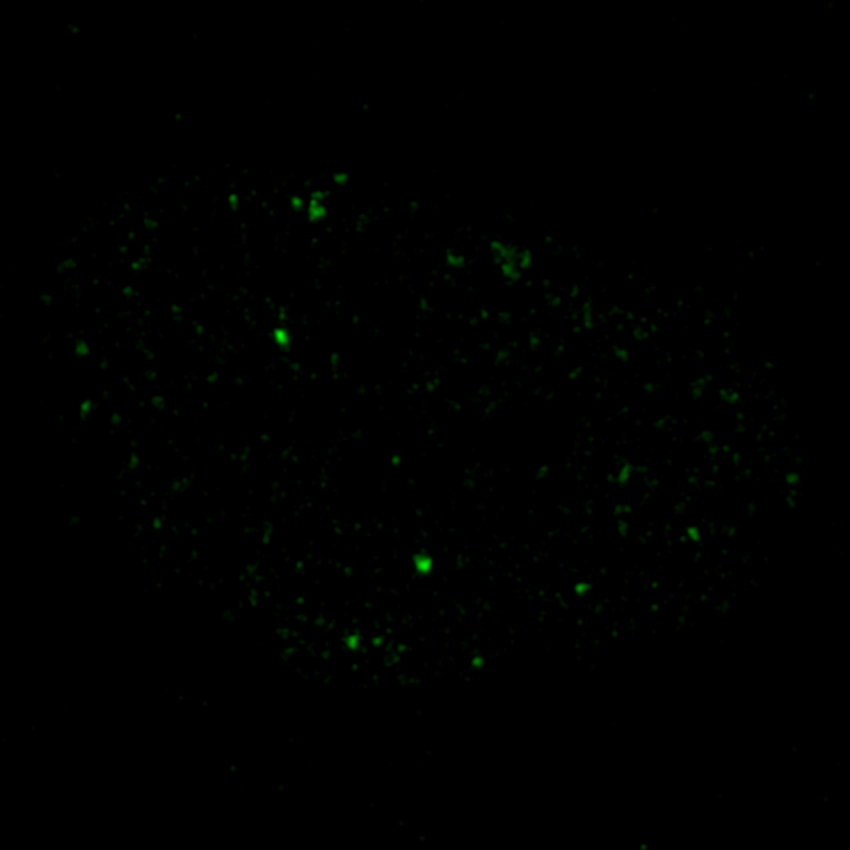

Supplement: Supplementary file 8 — Source data Fig. 5 [file 44318_2025_574_MOESM8_ESM.zip › Figure 5/5E/H2AX_nucleus2.png]

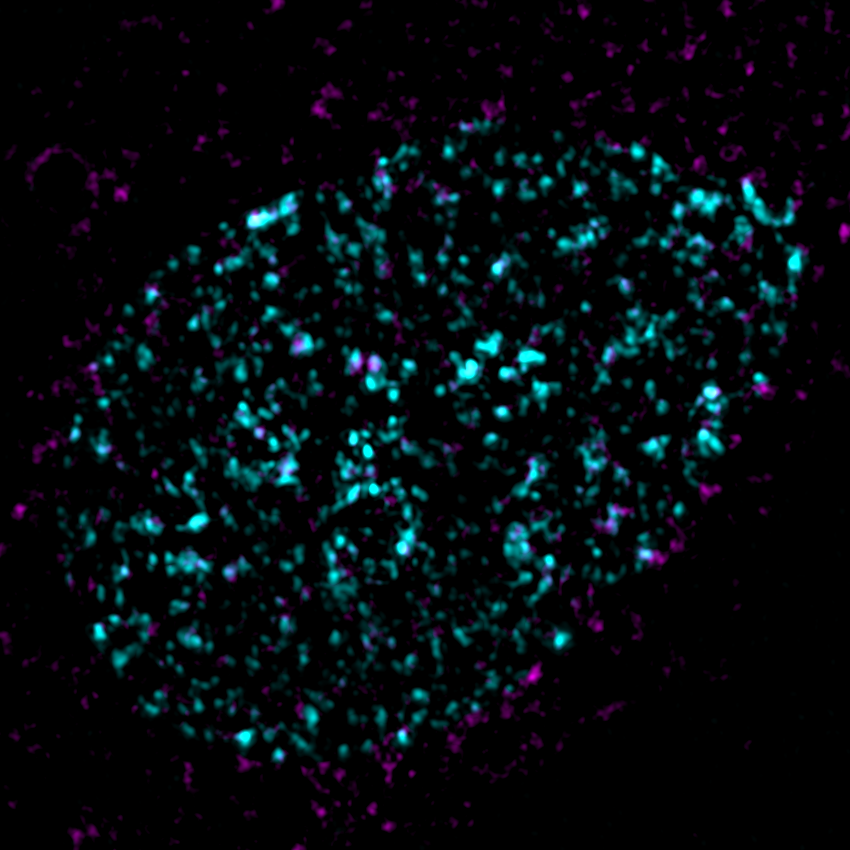

Supplement: Supplementary file 8 — Source data Fig. 5 [file 44318_2025_574_MOESM8_ESM.zip › Figure 5/5E/EdU_dUTP_HU_nucleus3.png]

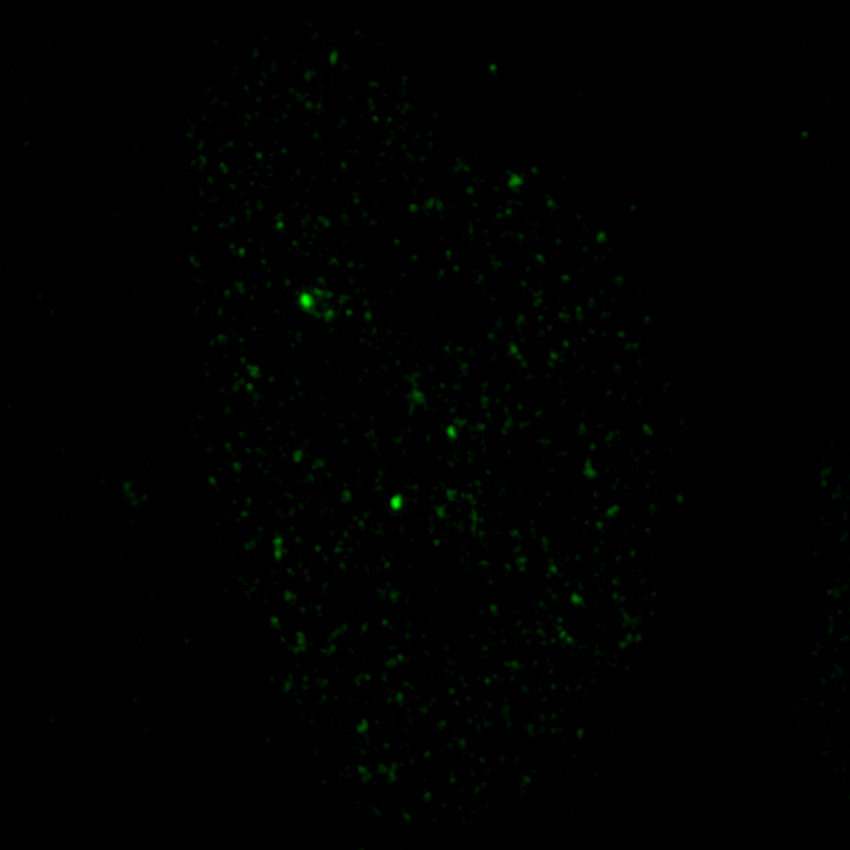

Supplement: Supplementary file 8 — Source data Fig. 5 [file 44318_2025_574_MOESM8_ESM.zip › Figure 5/5E/H2AX_nucleus1.png]

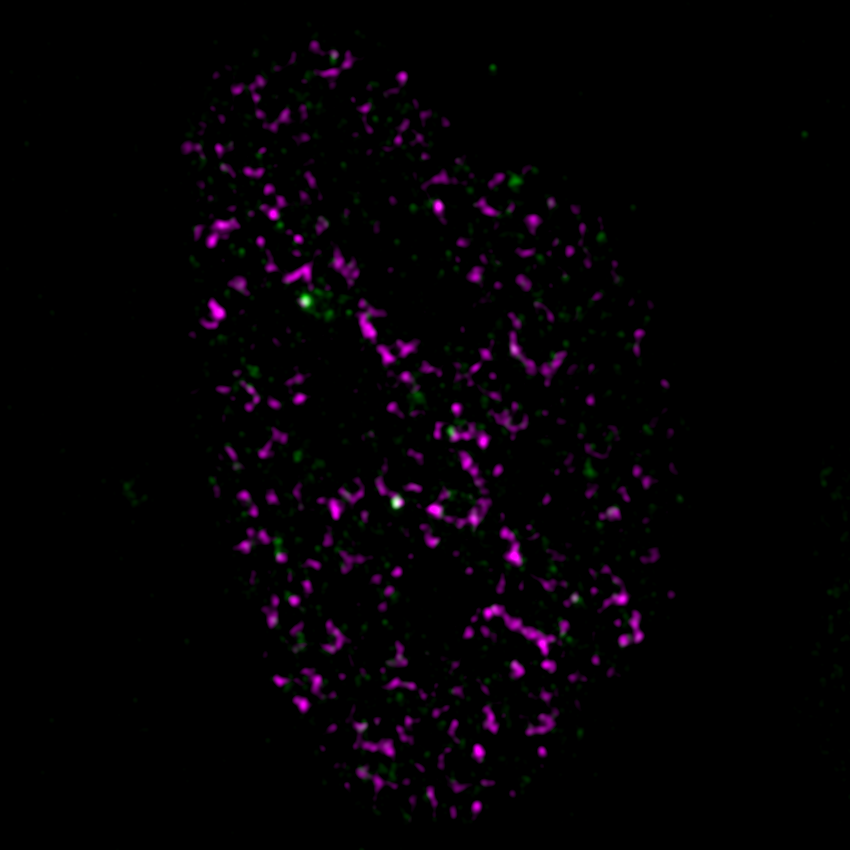

Supplement: Supplementary file 8 — Source data Fig. 5 [file 44318_2025_574_MOESM8_ESM.zip › Figure 5/5E/EdU_nucleus1.png]

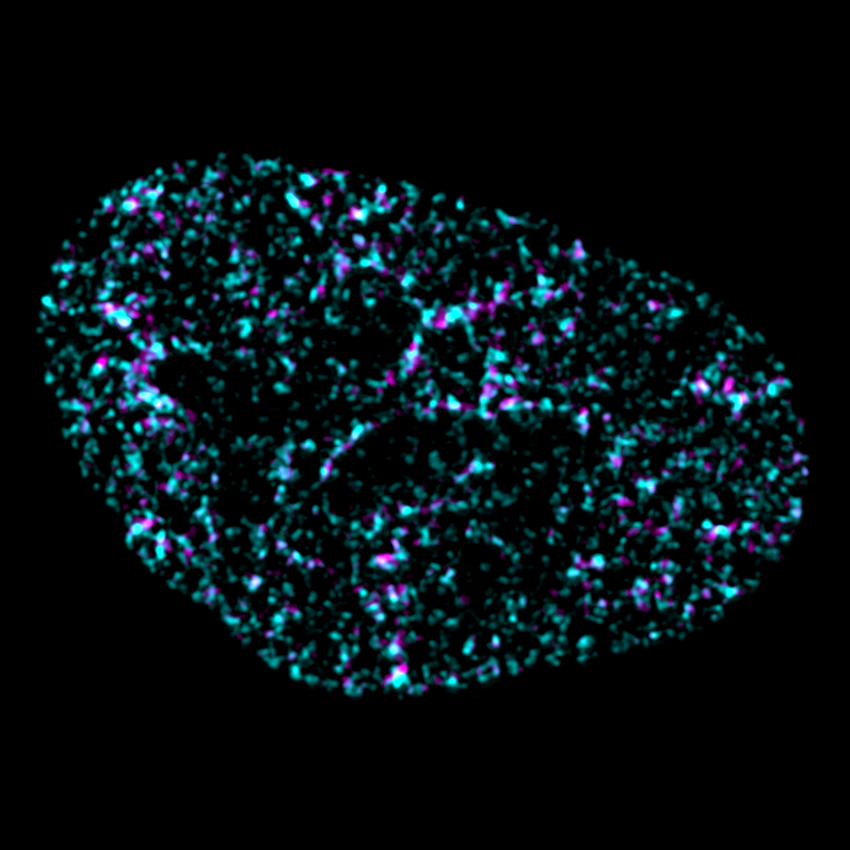

Supplement: Supplementary file 8 — Source data Fig. 5 [file 44318_2025_574_MOESM8_ESM.zip › Figure 5/5E/EdU_dUTP_nucleus2.png]

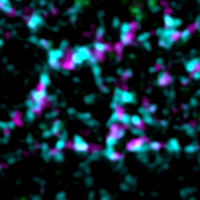

Supplement: Supplementary file 8 — Source data Fig. 5 [file 44318_2025_574_MOESM8_ESM.zip › Figure 5/5E/crop_merge_nucleus2.png]

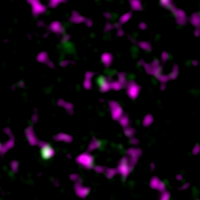

Supplement: Supplementary file 8 — Source data Fig. 5 [file 44318_2025_574_MOESM8_ESM.zip › Figure 5/5E/crop_merge_nucleus1.png]

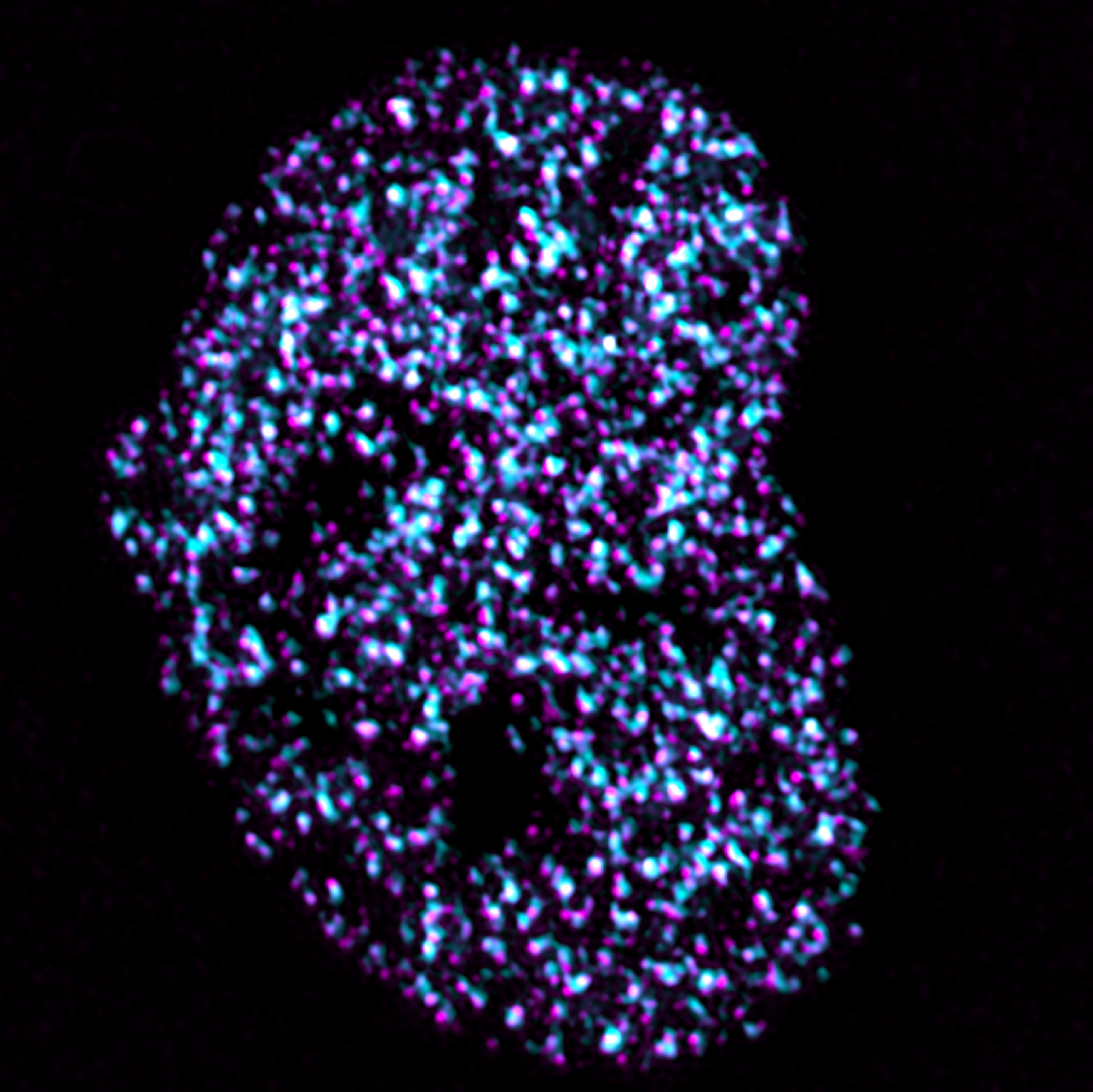

Supplement: Supplementary file 10 — Source data Fig. 7 [file 44318_2025_574_MOESM10_ESM.zip › Figure 7/7B/4h/Image 95_max.png]

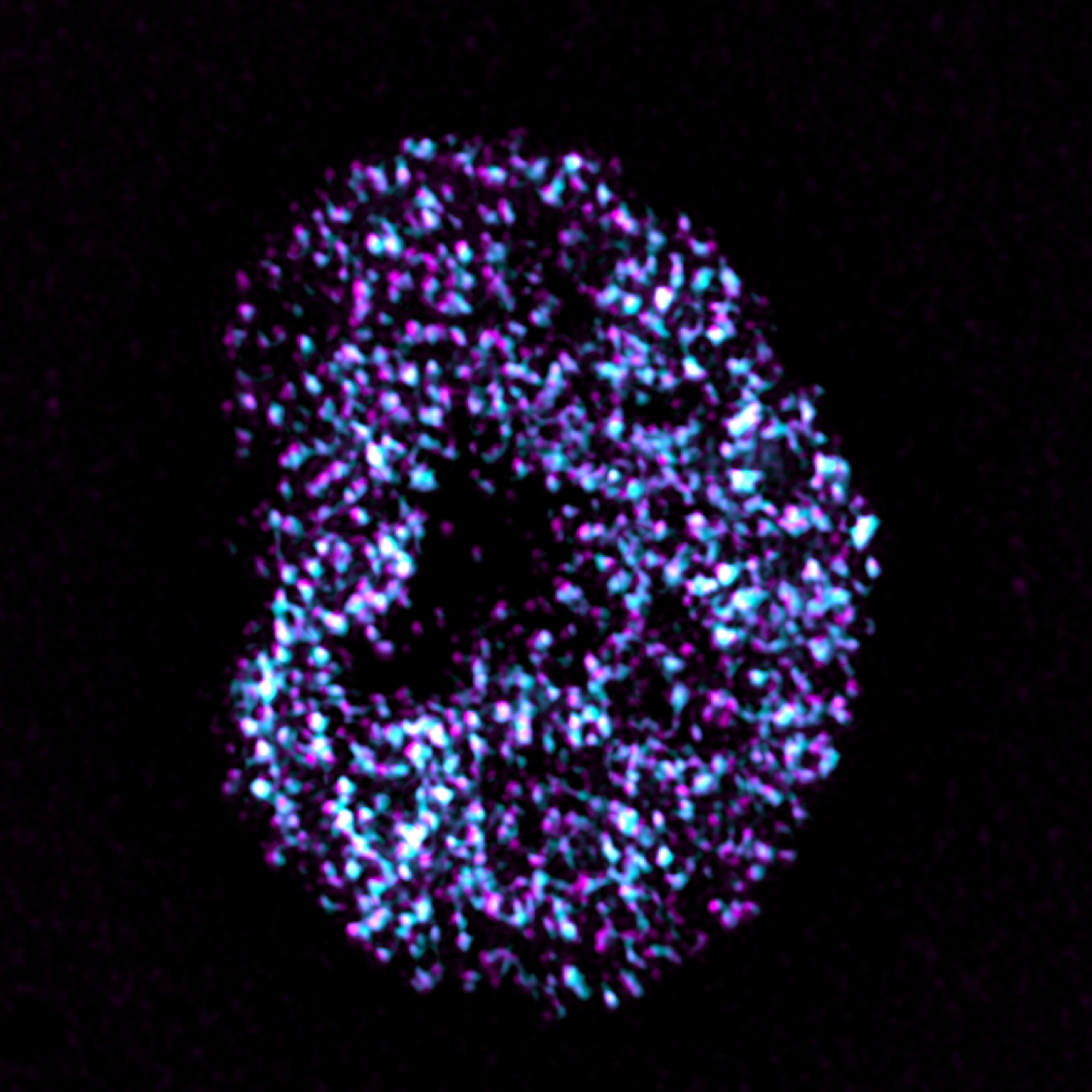

Supplement: Supplementary file 10 — Source data Fig. 7 [file 44318_2025_574_MOESM10_ESM.zip › Figure 7/7B/4h/Image 101_max.png]

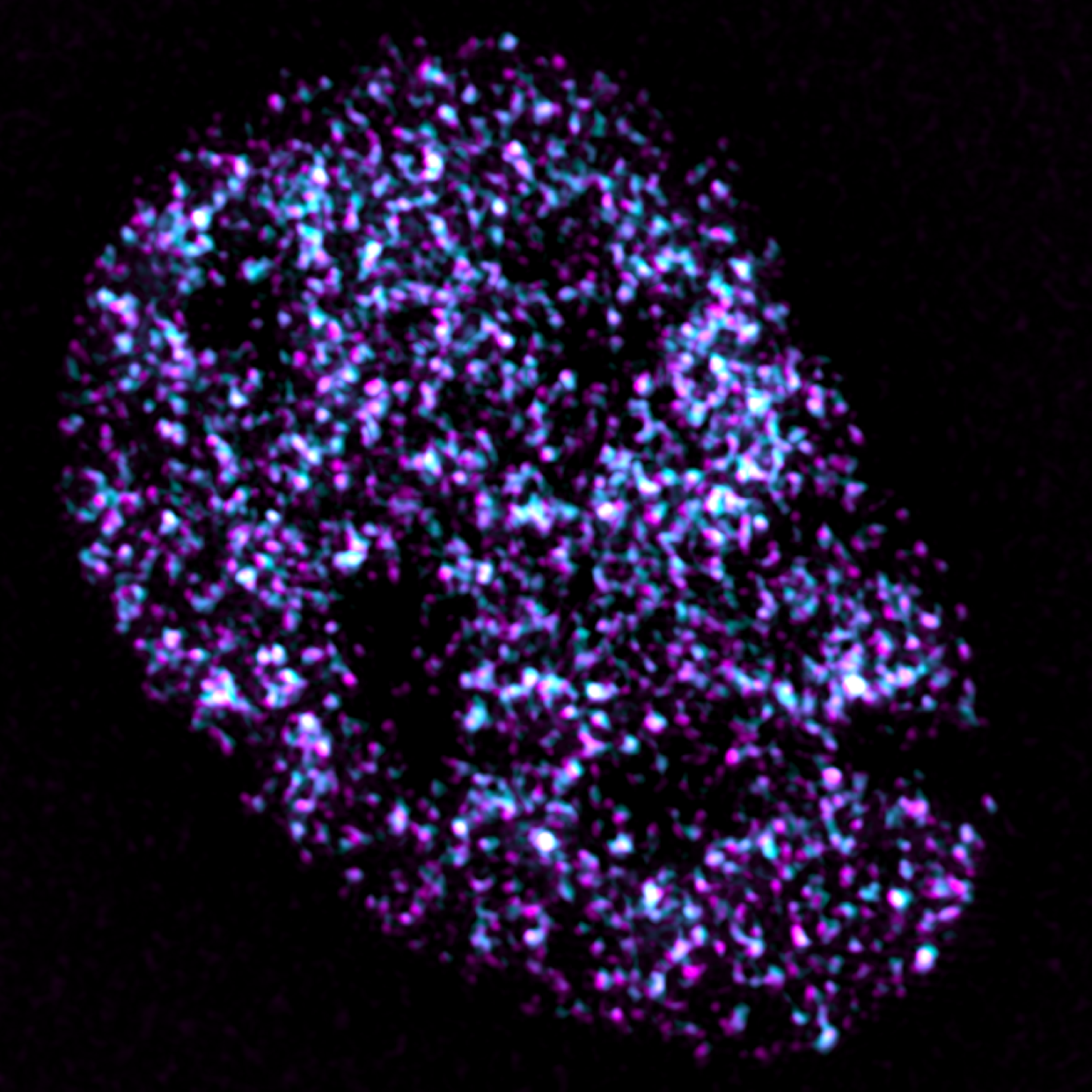

Supplement: Supplementary file 10 — Source data Fig. 7 [file 44318_2025_574_MOESM10_ESM.zip › Figure 7/7B/4h/Image 101A_max.png]

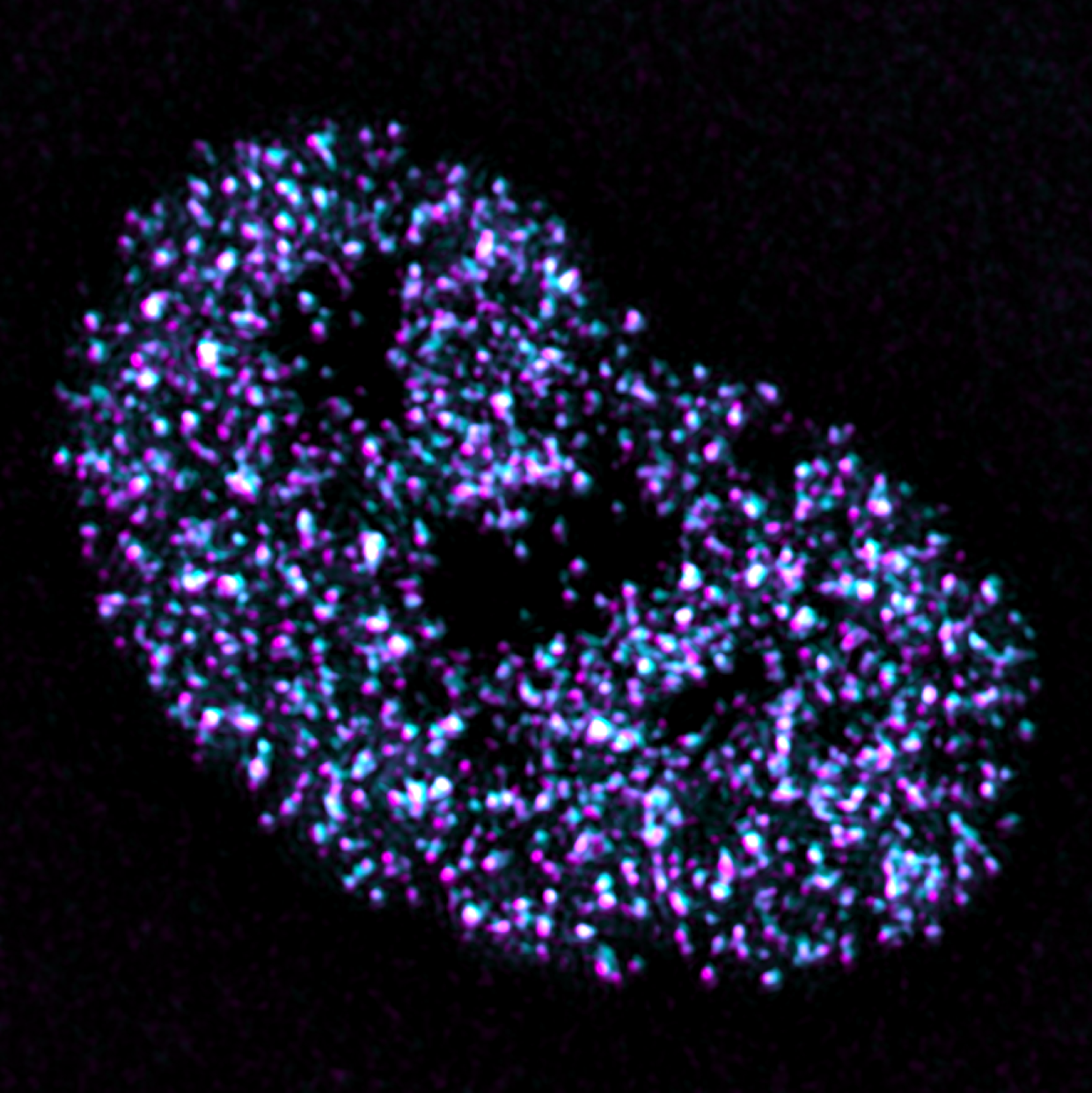

Supplement: Supplementary file 10 — Source data Fig. 7 [file 44318_2025_574_MOESM10_ESM.zip › Figure 7/7B/4h/Image 113_max.png]

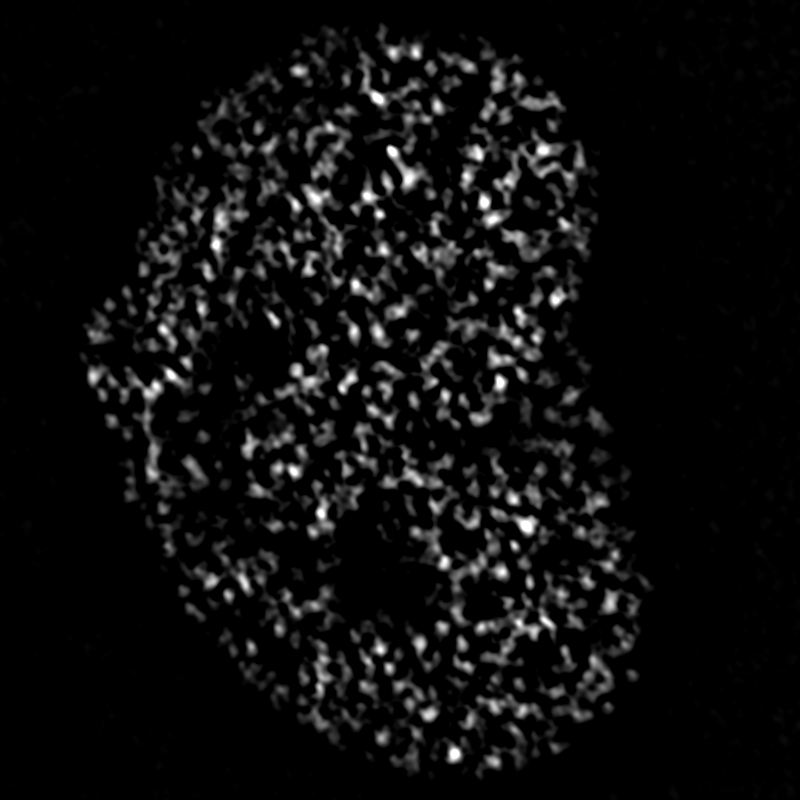

Supplement: Supplementary file 10 — Source data Fig. 7 [file 44318_2025_574_MOESM10_ESM.zip › Figure 7/7B/4h/Image 95_single_slice.tif]

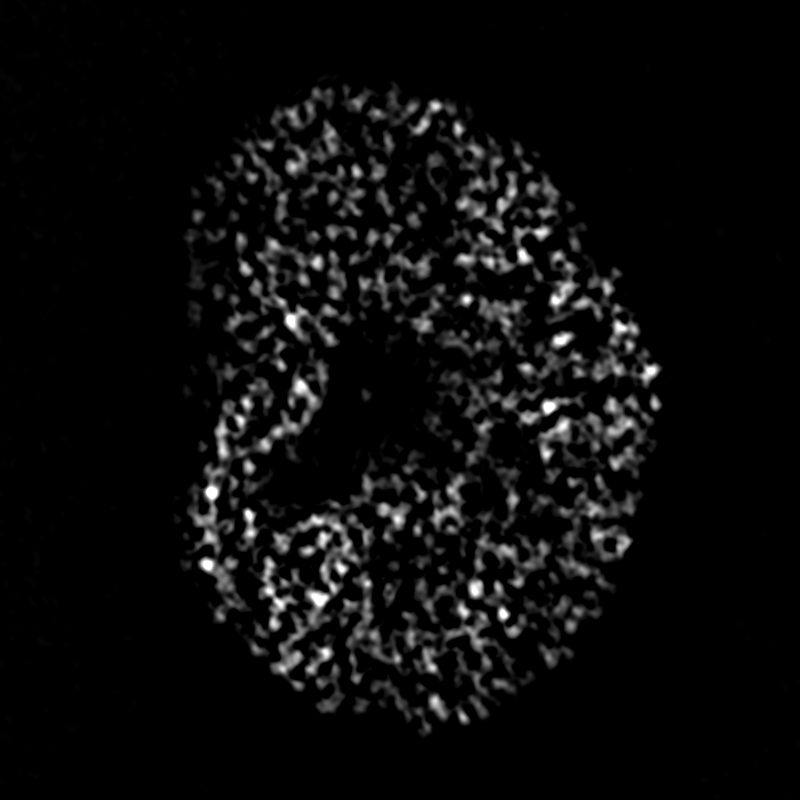

Supplement: Supplementary file 10 — Source data Fig. 7 [file 44318_2025_574_MOESM10_ESM.zip › Figure 7/7B/4h/Image 101_single_slice.tif]

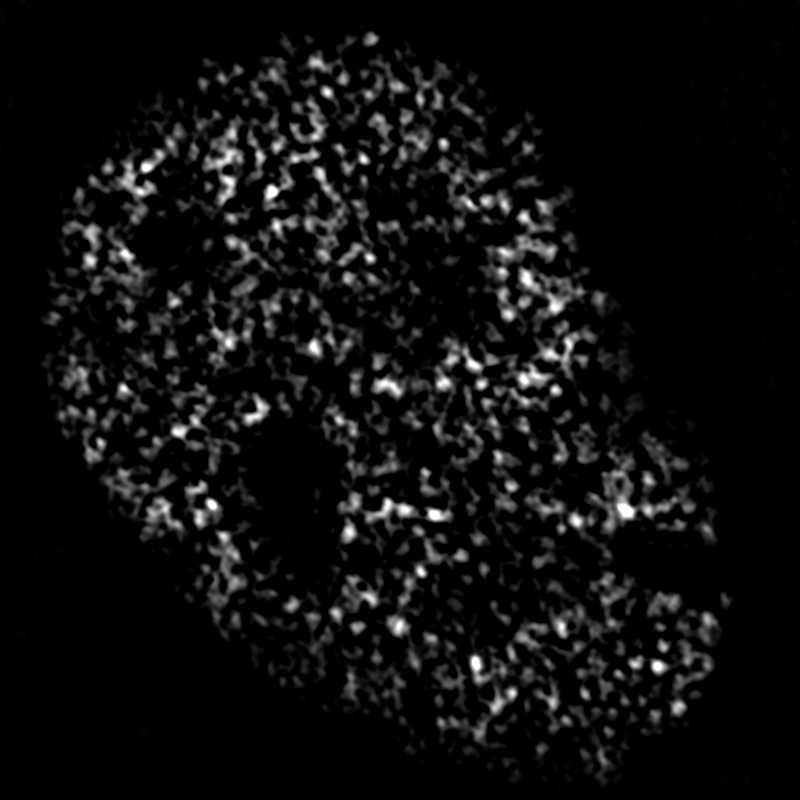

Supplement: Supplementary file 10 — Source data Fig. 7 [file 44318_2025_574_MOESM10_ESM.zip › Figure 7/7B/4h/Image 101a_single_slice.tif]

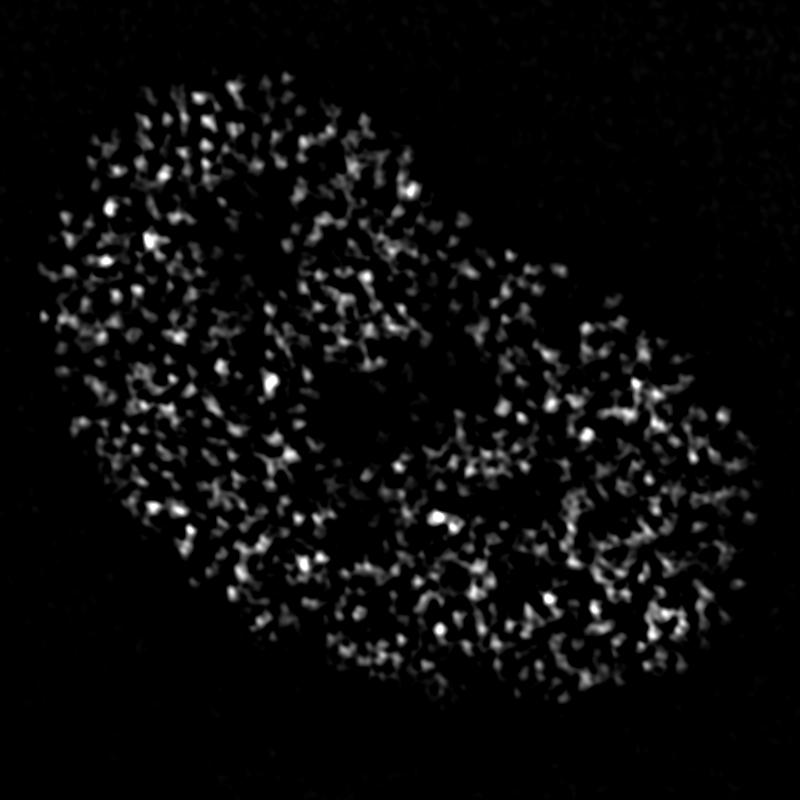

Supplement: Supplementary file 10 — Source data Fig. 7 [file 44318_2025_574_MOESM10_ESM.zip › Figure 7/7B/4h/Image 113_single_slice.tif]

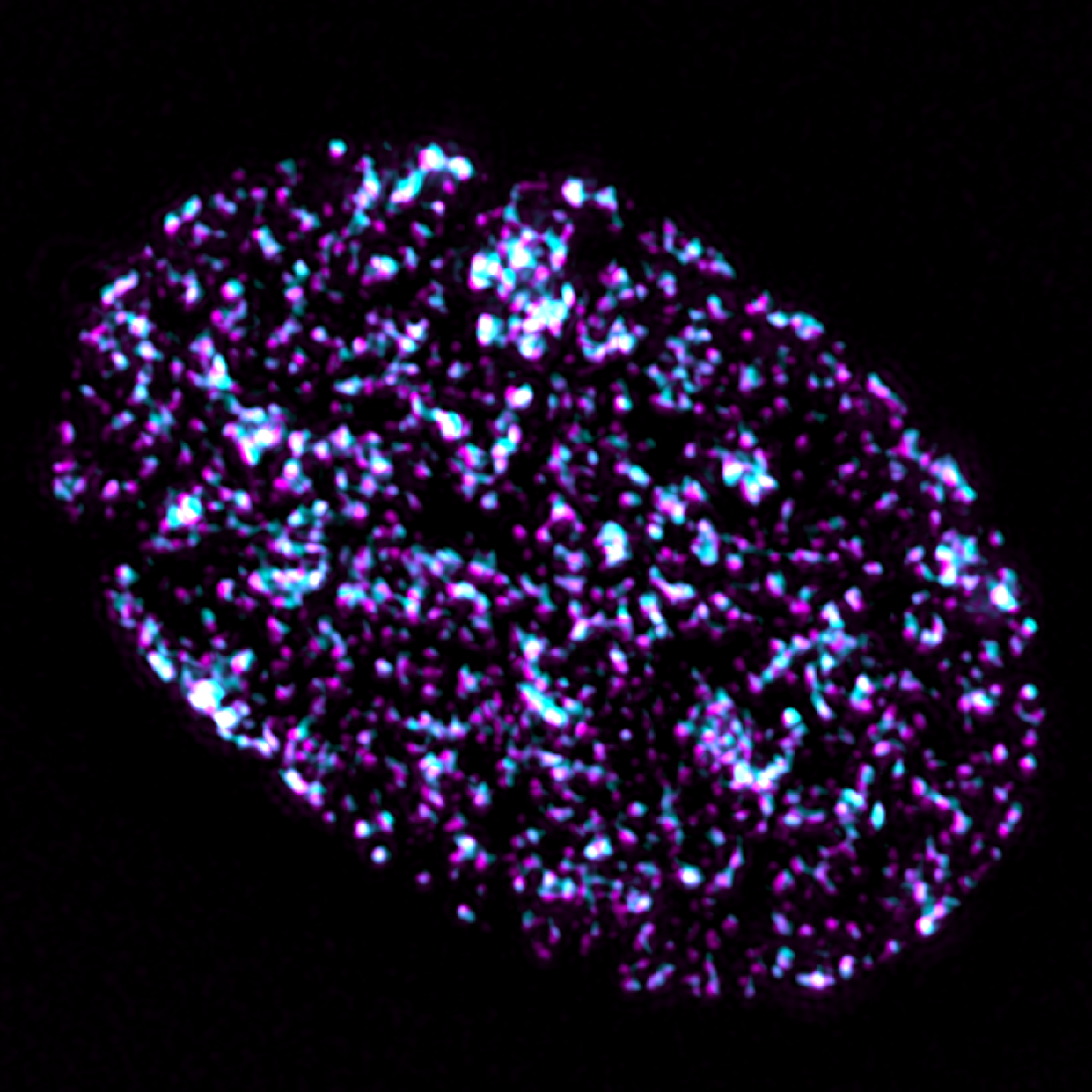

Supplement: Supplementary file 10 — Source data Fig. 7 [file 44318_2025_574_MOESM10_ESM.zip › Figure 7/7B/8h/Image 15_max.png]

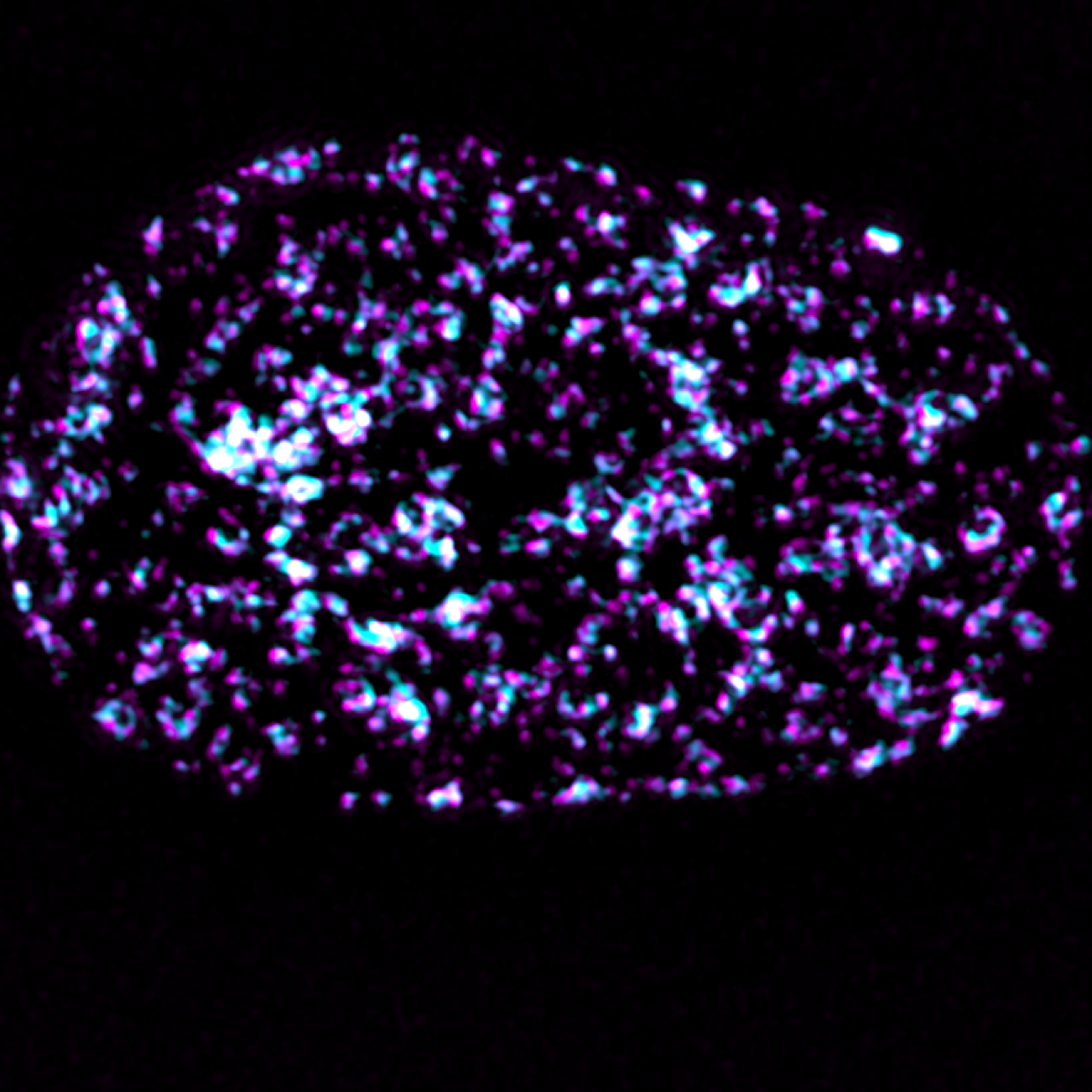

Supplement: Supplementary file 10 — Source data Fig. 7 [file 44318_2025_574_MOESM10_ESM.zip › Figure 7/7B/8h/Image 20_max.png]

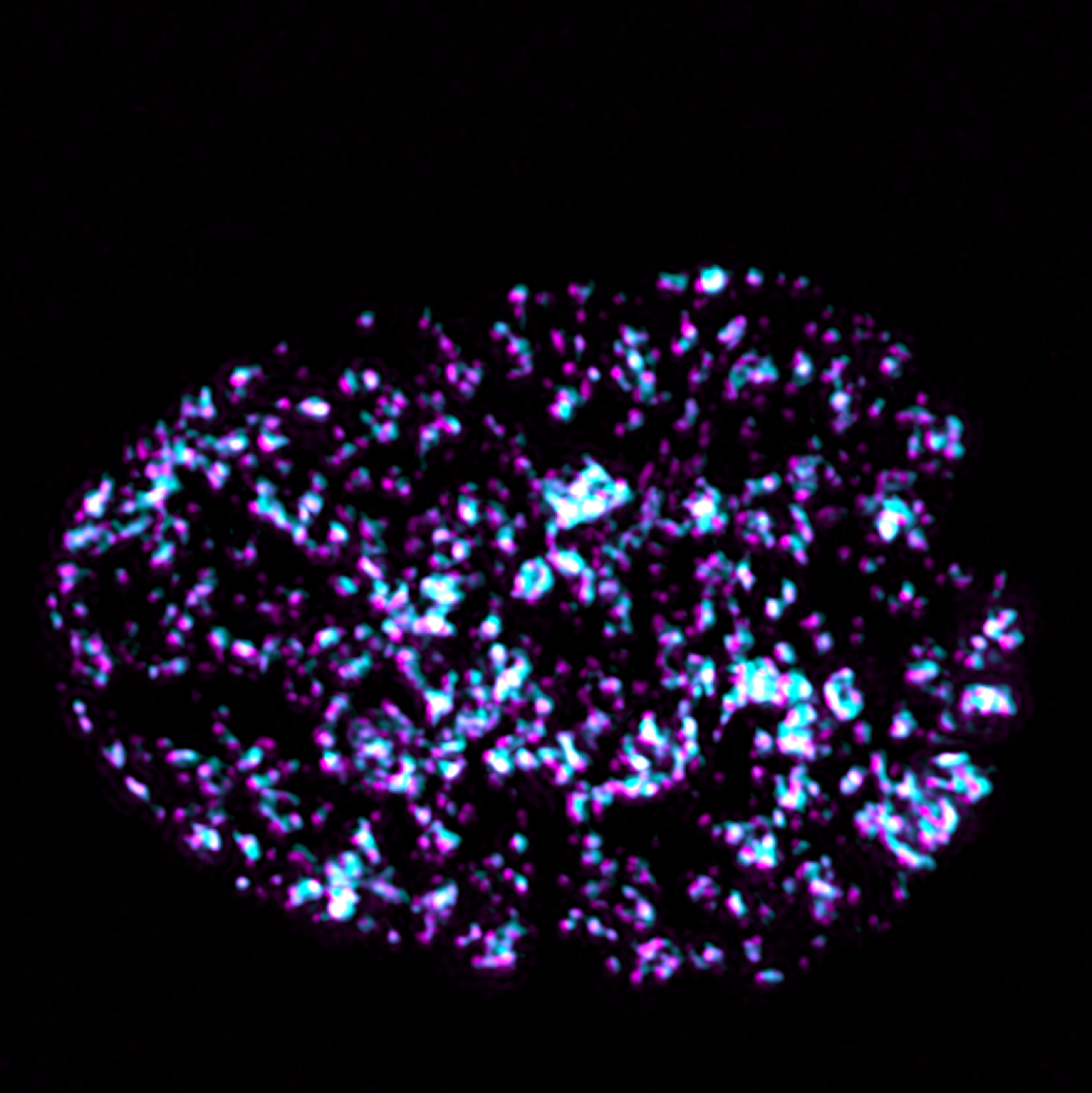

Supplement: Supplementary file 10 — Source data Fig. 7 [file 44318_2025_574_MOESM10_ESM.zip › Figure 7/7B/8h/Image 32_max.png]

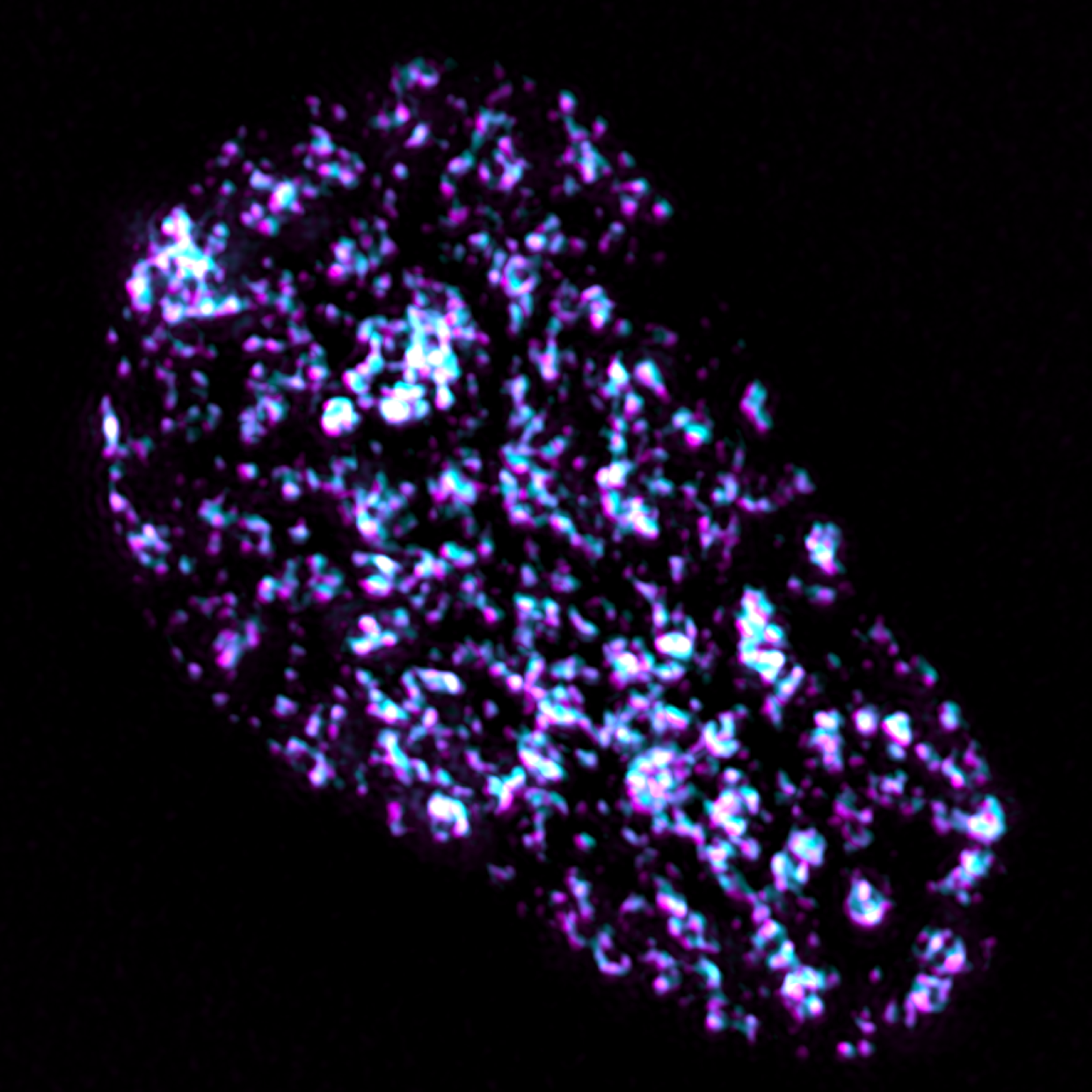

Supplement: Supplementary file 10 — Source data Fig. 7 [file 44318_2025_574_MOESM10_ESM.zip › Figure 7/7B/8h/Image 38_max.png]

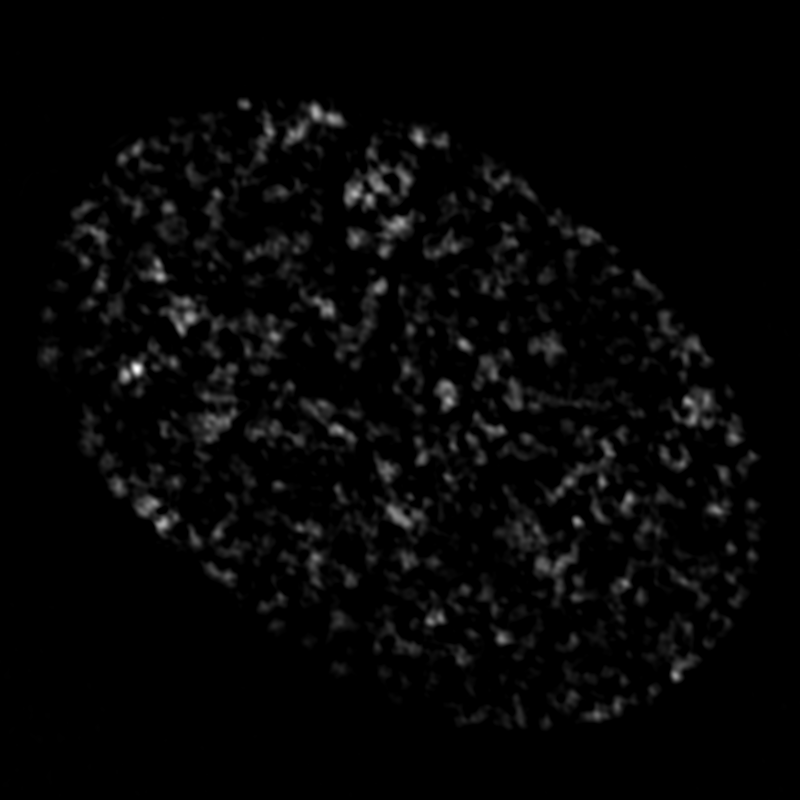

Supplement: Supplementary file 10 — Source data Fig. 7 [file 44318_2025_574_MOESM10_ESM.zip › Figure 7/7B/8h/Image 15_single_slice.tif]

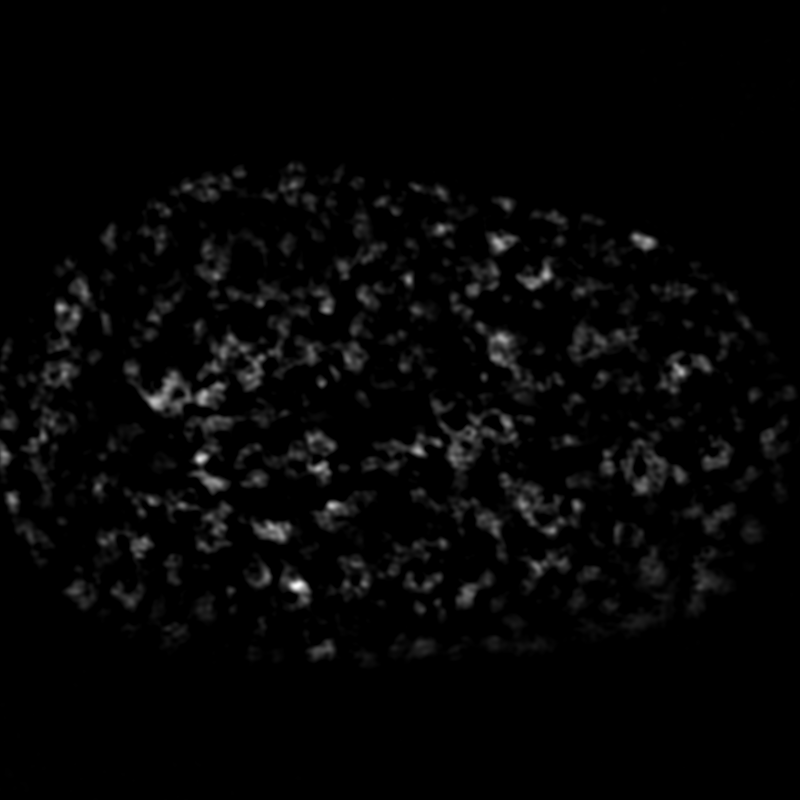

Supplement: Supplementary file 10 — Source data Fig. 7 [file 44318_2025_574_MOESM10_ESM.zip › Figure 7/7B/8h/Image 20_single_slice.tif]

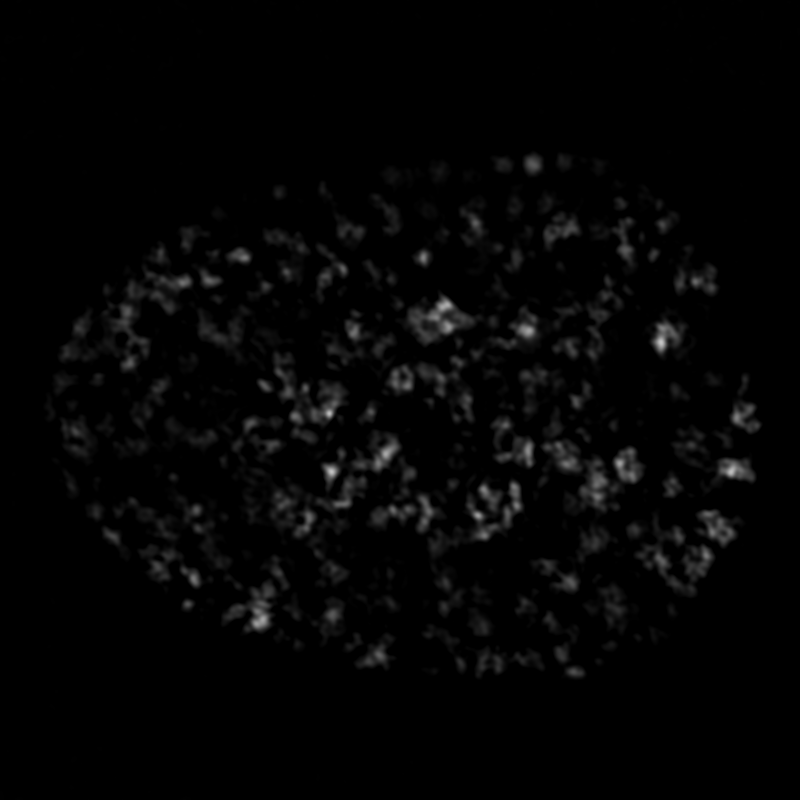

Supplement: Supplementary file 10 — Source data Fig. 7 [file 44318_2025_574_MOESM10_ESM.zip › Figure 7/7B/8h/Image 32_single_slice.tif]

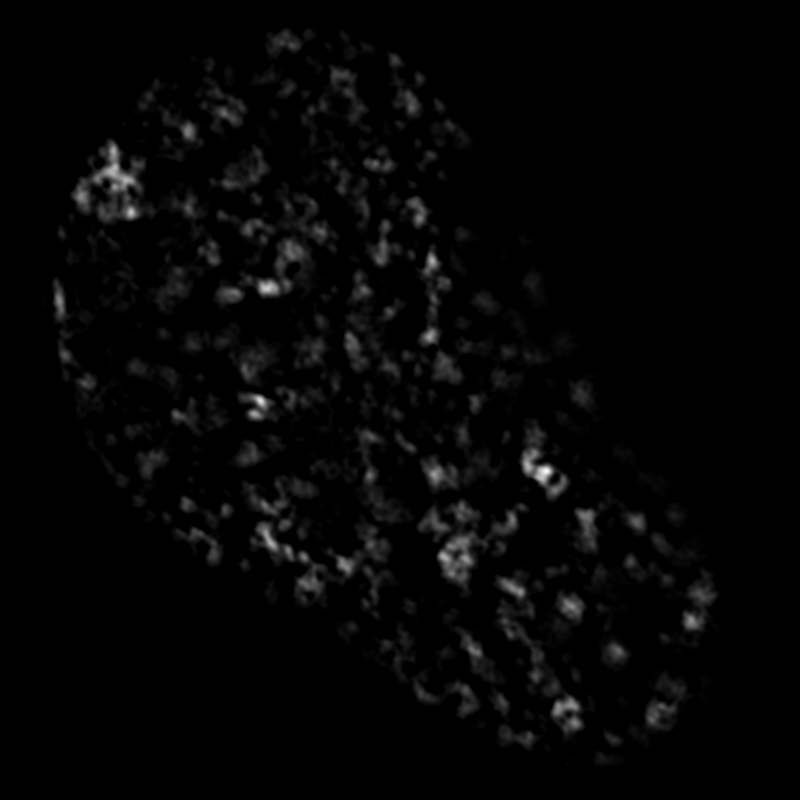

Supplement: Supplementary file 10 — Source data Fig. 7 [file 44318_2025_574_MOESM10_ESM.zip › Figure 7/7B/8h/Image 38_single_slice.tif]
