# Supplementary material for: Spatial mapping of DNA synthesis reveals dynamics and geometry of human replication nanostructures
Source: EMBO J. 2025 Oct 7;44(23):7263–94. doi: 10.1038/s44318-025-00574-2 (PMC12669658; doi:10.1038/s44318-025-00574-2)
Supplement: Supplementary file 12 — Expanded View Figures [file 44318_2025_574_MOESM12_ESM.pdf]

Expanded View Figures

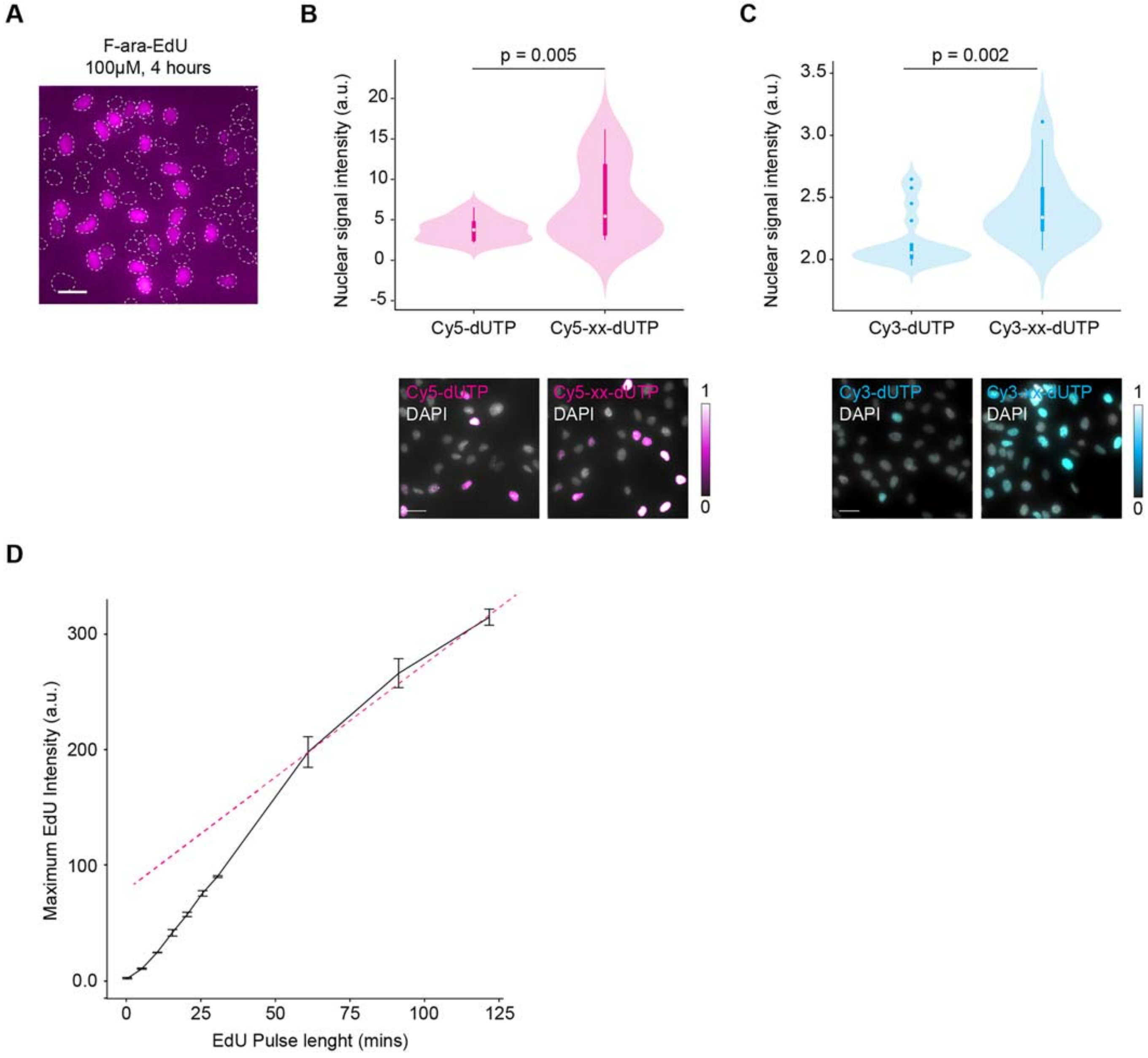

Figure EV1. Additional characterisation of F-ara-EdU, fluorescent dUTP linker variants and EdU pulses.

(**A**) Representative widefield fluorescence image after 4 h, 100 μM F-ara-EdU. Nuclei are annotated with a white dashed line; scale bar: 20 μm. (**B**) Violin/box plot showing the S phase nuclear signal intensities (a.u.) of Cy5-dUTP with and without an aminoallyl linker molecule (xx) of three independent experiments. Representative images are shown below, scale bar: 20 μm. For each box, the inner line indicates median and box limits show 25th and 75th percentiles. Whiskers extend to edge values within 1.5 times the interquartile range between 25th and 75th percentiles from the box limits. Dots represent values beyond whisker range. (**C**) Violin/box plot showing the S phase nuclear signal intensities (a.u.) of Cy3-dUTP with and without an aminoallyl linker molecule (xx) of three independent experiments. Representative images are shown below, scale bar: 20 μm. For each box, the inner line indicates median and box limits show 25th and 75th percentiles. Whiskers extend to edge values within 1.5 times the interquartile range between 25th and 75th percentiles from the box limits. Dots represent values beyond whisker range. (**D**) Line plot (black line) showing the average 95th percentile EdU intensity upon different EdU pulse durations (0, 5, 10, 15, 20, 25, 30, 90, 120 min). Cyan line highlights the linear trend of pulses ≥ 60 min, similar to the observation by Pereira et al Oncotarget 2017. Three replicates per condition; error bars represent SEM; At least 15,000 cells per conditions were analysed.

© The Author(s)

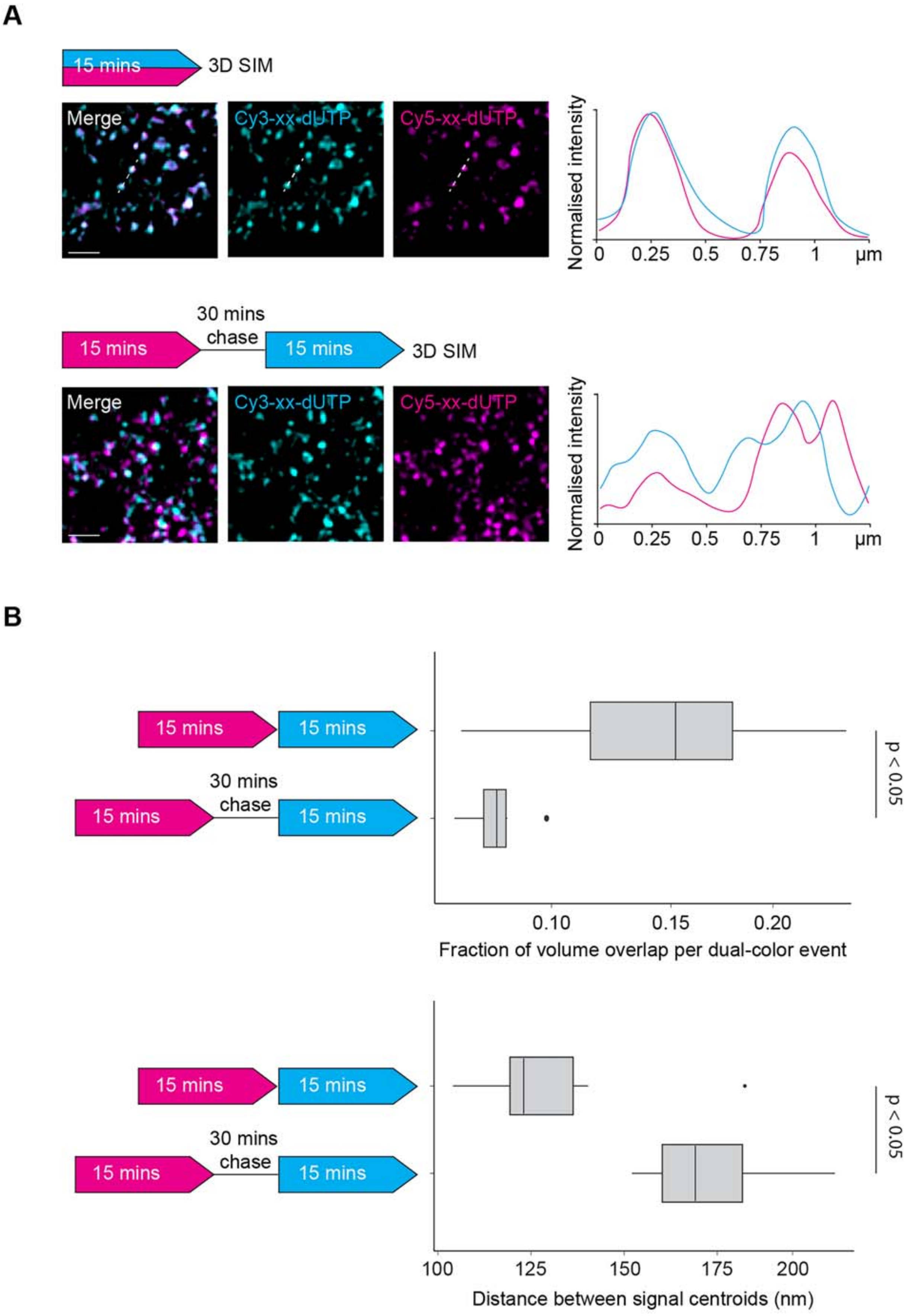

A
15 mins
3D SIM
Merge
Cy3-xx-dUTP
Cy5-xx-dUTP
Normalised intensity
0
0.25
0.5
0.75
1
µm
30 mins chase
15 mins
15 mins
3D SIM
Merge
Cy3-xx-dUTP
Cy5-xx-dUTP
Normalised intensity
0
0.25
0.5
0.75
1
µm
B
15 mins
15 mins
30 mins chase
15 mins
15 mins
p < 0.05
0.10
0.15
0.20
Fraction of volume overlap per dual-color event
15 mins
15 mins
30 mins chase
15 mins
15 mins
p < 0.05
100
125
150
175
200
Distance between signal centroids (nm)

 © The Author(s)

Figure EV2. Pulse-chase studies verifying replication foci dynamics detected by 3D-SPARK.

(**A**) Representative 2D maximum projections (3D-SIM) of a nuclear region after a simultaneous pulse of Cy5-xx-dUTP and Cy3-xx-dUTP at a 1:1 ratio (upper panel) or after a 15-min pulse of Cy3-xx-dUTP, followed by a 30-min chase period and a final Cy5-xx-dUTP pulse. For each condition single wavelength and merged images are shown; scale bar = 1 µm. The line graph shows the normalised signal intensity profile across the depicted white dashed line. (**B**) Box plot reporting the fraction of volume overlap per dual-colour event (upper panel) and the distance between signal centroids (lower panel), following the EdU (magenta) and Cy3-xx-dUTP (blue) pulsing schemes indicated on the left: two consecutive pulses ($n = 6$ cells) or two pulses divided by a 30-min chase ($n = 7$ cells). For each box, the inner line indicates median and box limits show 25th and 75th percentiles. Whiskers extend to edge values within 1.5 times the interquartile range between 25th and 75th percentiles from the box limits. Dots represent values beyond whisker range. *P* values were calculated with Mann–Whitney *U*-test and adjusted for multiple testing using Bonferroni correction.

© The Author(s)

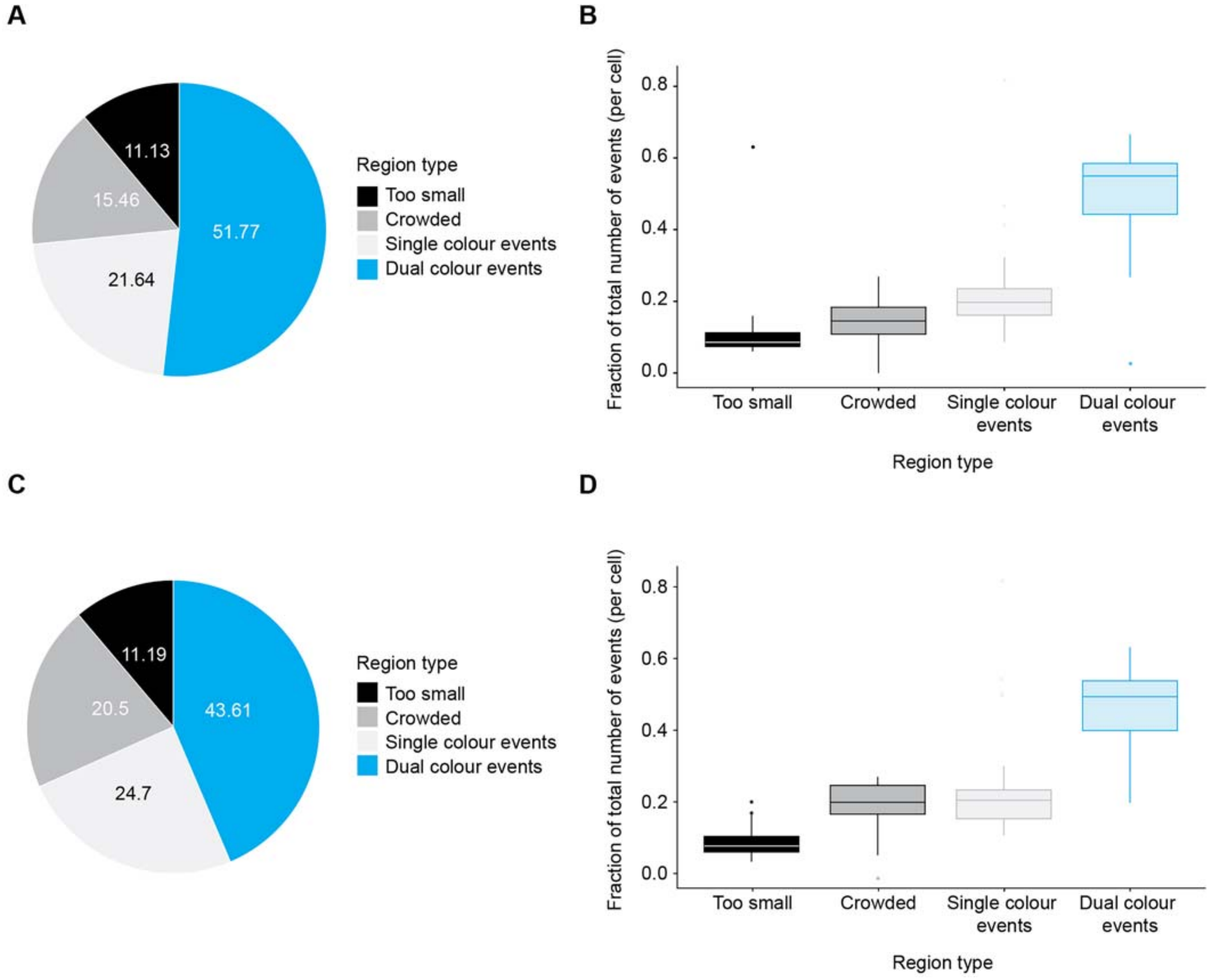

Figure EV3. Classification and relative proportions of fluorescent regions defined in image analysis.

(**A**) Pie chart showing the percentage of different regions defined during image analysis of untreated early-S phase RPE1 cells ($n = 28$ cells). Regions that are defined as too small, *crowded* or single colour events, are removed from final analysis. Dual colour events, are used in the replication dynamics analysis. (**B**) Box plot showing the fraction of total number of events per RPE1 cell of different regions defined during image analysis. For each box, the inner line indicates median and box limits show 25th and 75th percentiles. Whiskers extend to edge values within 1.5 times the interquartile range between 25th and 75th percentiles from the box limits. Dots represent values beyond whisker range. (**C**) Pie chart showing the percentage of different regions defined during image analysis of untreated early-S phase RPE FRT/TR cells ($n = 17$ cells) (Chiang et al, 2016). Regions that are defined as too small, crowded or single colour events, are removed from final analysis. Dual colour events, are used in the replication dynamics analysis. (**D**) Box plot showing total number of dual colour events per cell in early-S phase RPE1 and RPE FRT/TR cells selected for replication dynamics analysis. For each box, the inner line indicates median and box limits show 25th and 75th percentiles. Whiskers extend to edge values within 1.5 times the interquartile range between 25th and 75th percentiles from the box limits. Dots represent values beyond whisker range.

© The Author(s)

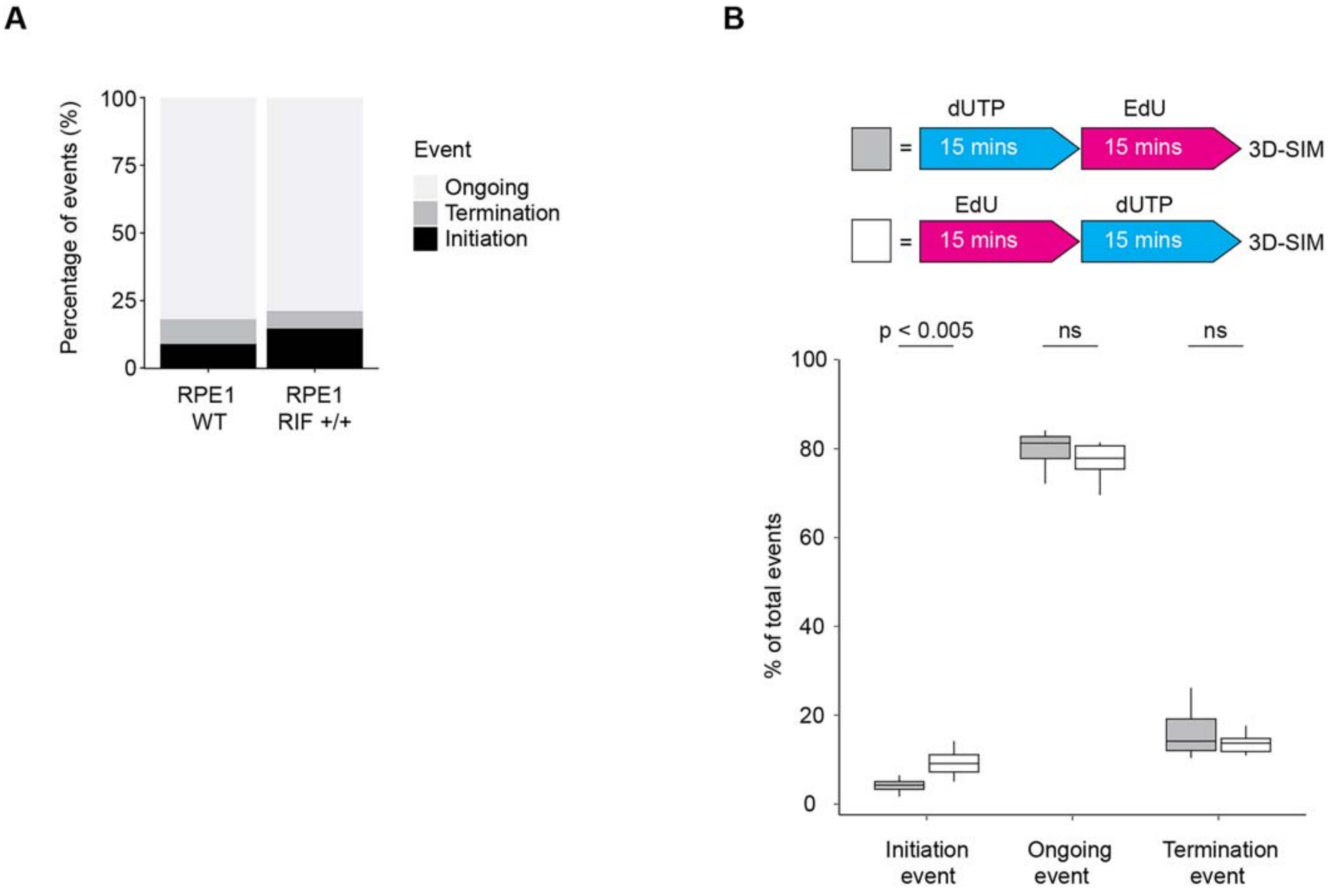

Figure EV4. The proportion of different types of dual colour events.

(**A**) Stacked bar graph shows relative percentage of ongoing, initiation and termination events in early-S phase RPE1 ($n = 10{,}390$ total number of dual colour events) and RPE FRT/TR cells ($n = 4957$ total number of dual colour events). (**B**) Box plot showing the percentage of event (initiation, ongoing, termination) on the total events, following the classic pulse scheme (EdU followed by dUTP, $n = 6$ cells) or the reversed one (dUTP followed by EdU, $n = 9$ cells). For each box, the inner line indicates median and box limits show 25th and 75th percentiles. Whiskers extend to edge values within 1.5 times the interquartile range between 25th and 75th percentiles from the box limits. Dots represent values beyond whisker range. P values were calculated with Mann–Whitney *U*-test and adjusted for multiple testing using Bonferroni correction.

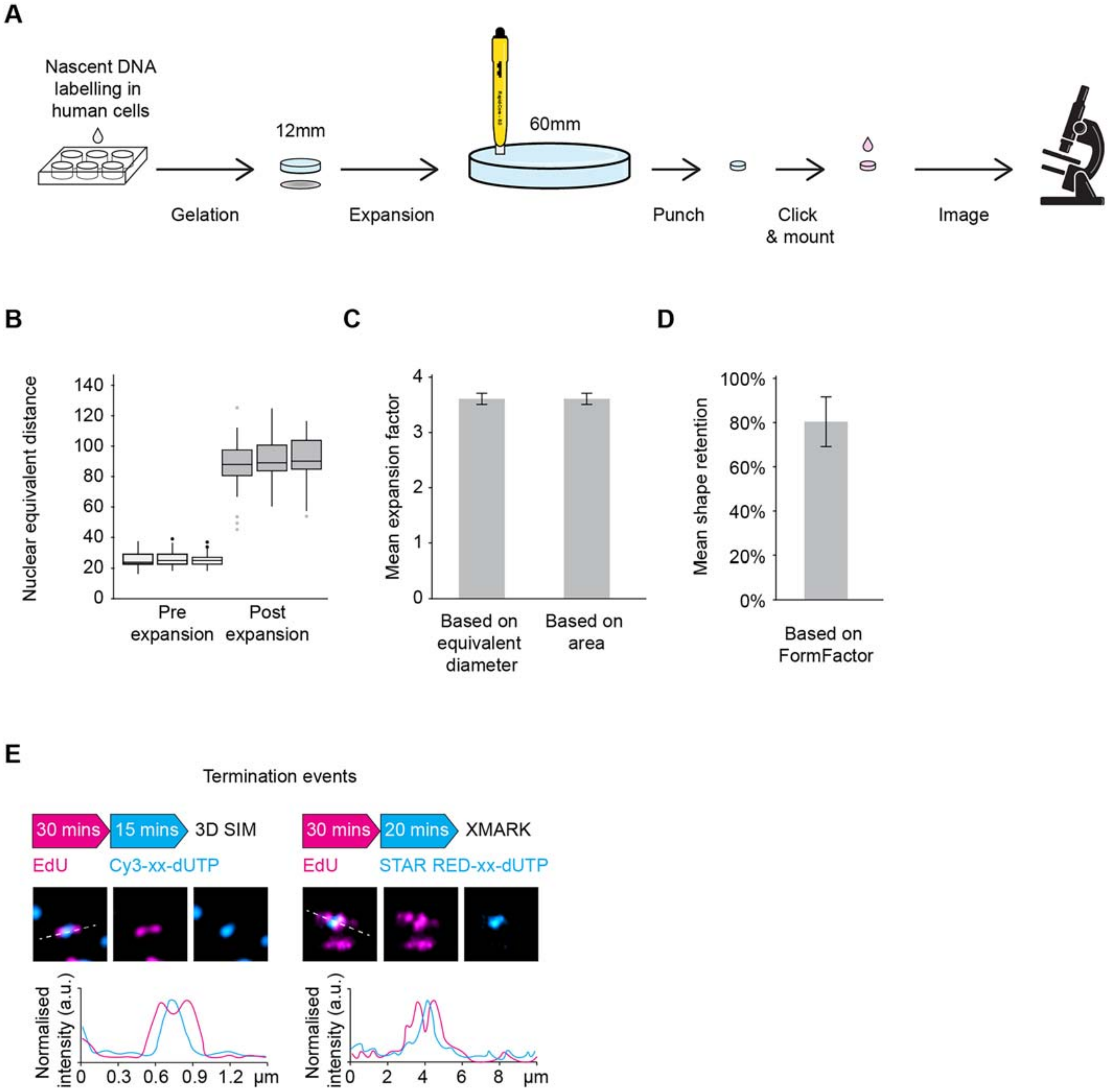

Figure EV5. General outline and validation of XMARK protocol.

(**A**) Illustration of the XMARK procedure. Cells adhered to coverslips undergo nascent DNA pulse labelling and are fixed. Gels are then formed on the coverslip, embedding the fixed cells within the gel. The formed gel is expanded, and a small section is cut, using a tissue biopsy punch tool. The cut section undergoes click chemistry and the expanded gel is mounted for confocal imaging. (**B**) Box plot showing the nuclear equivalent diameter of DAPI-stained single nuclei pre- and post-expansion determined by 20X wide-field microscopy and Cell Profiler software. Distribution of nuclear equivalent diameters of three independent experiments are shown ($n = 100$ cells per condition). For each box, the inner line indicates median and box limits show 25th and 75th percentiles. Whiskers extend to edge values within 1.5 times the interquartile range between 25th and 75th percentiles from the box limits. Dots represent values beyond whisker range. (**C**) Bar plot showing the mean expansion factor based on nuclear equivalent diametr (as depicted in **B**) and nuclear area (i.e. square root of the mean area of expanded nuclei divided by mean area of non-expanded nuclei). (**D**) Bar plot showing the mean shape retention post expansion based on FormFactor determined by 20X wide-field microscopy and Cell Profiler software. FormFactors pre- and post-expansion is calculated as $4*\pi*\text{Area}/\text{Perimeter}^2$. (**E**) Representative 2D maximum projection images of selected examples of termination events from a 3D-SIM and XMARK image shown in Fig. 4B, C, respectively. The line graphs show the normalised signal intensity profile (a.u.) across the termination events, illustrated by the white dashed line in the merged image.

 © The Author(s)

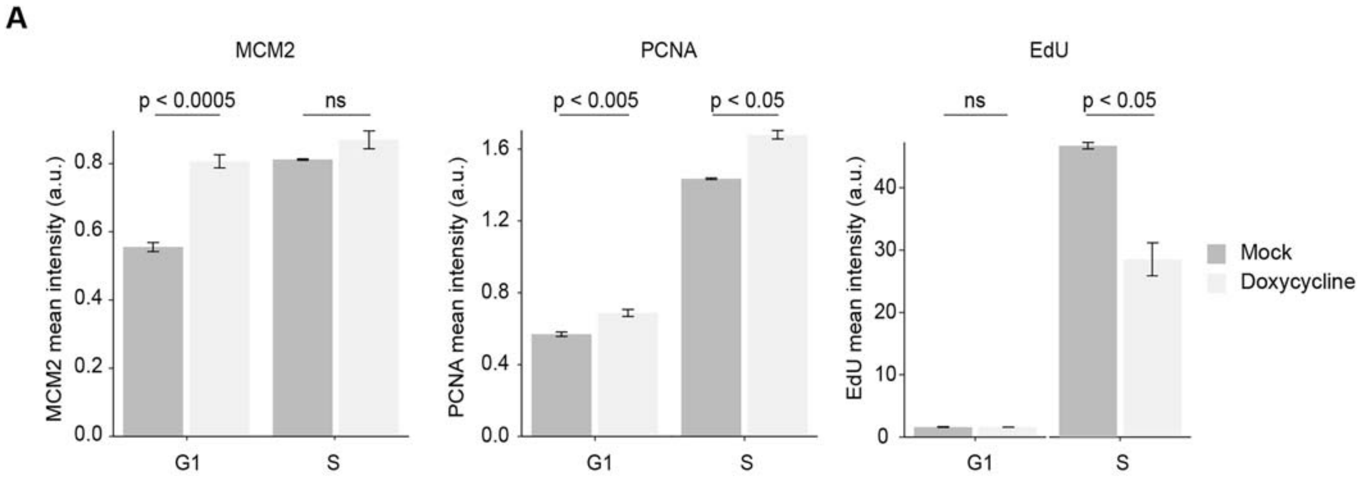

Figure EV6. Quantification of origin licensing and DNA synthesis in HBEC CDC6 TetON cells.

(**A**) Bar plot showing the mean nuclear integrated intensities (a.u.) of MCM2, PCNA and EdU for asynchronous cells treated with Doxycycline for 24 h and DMSO control. G1 and S population were selected based on DAPI vs EdU. Three replicates per condition, error bars represent S.E.M (for each condition, at least 10,000 cells were analysed for G1 portion and at least 2000 cells were analysed for the S portion).

© The Author(s)

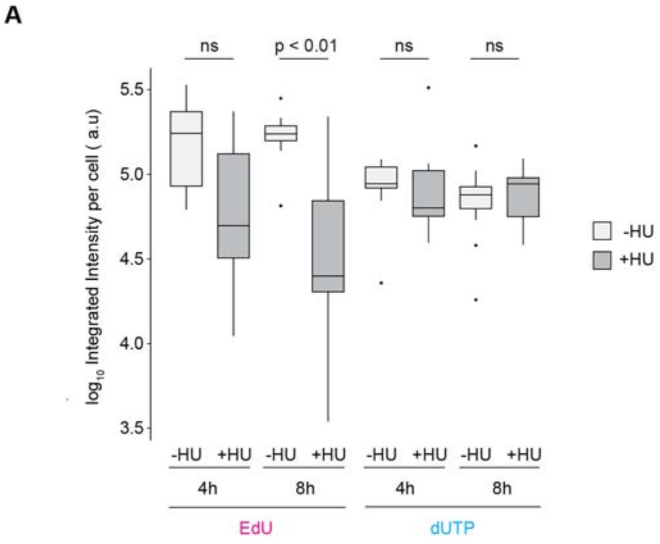

Figure EV7. Signal intensity analysis of 3D-SPARK nano foci.

(**A**) Box plot showing the nuclear integrated intensities (a.u.) of EdU and dUTP per 3D-SPARK-imaged synchronised cells with palbociclib and released for a certain amount of time prior to labelling. Number of nuclei: 10 (4 h, mock); 12 (4 h, HU); 13 (8 h, mock); 12 (8 h, HU). For each box, the inner line indicates median and box limits show 25th and 75th percentiles. Whiskers extend to edge values within 1.5 times the interquartile range between 25th and 75th percentiles from the box limits. Dots represent values beyond whisker range. P values were calculated with Mann–Whitney *U*-test and adjusted for multiple testing using Bonferroni correction. *P*: 4.8e-4 (EdU, 8 h, mock vs HU).

 © The Author(s)
